# Exploration of Nitrotyrosine-Containing Proteins and Peptides by Antibody-Based Enrichment Strategies

**DOI:** 10.1016/j.mcpro.2024.100733

**Published:** 2024-02-10

**Authors:** Firdous A. Bhat, Kiran K. Mangalaparthi, Husheng Ding, Anu Jain, Joel-Sean Hsu, Jane A. Peterson, Roman M. Zenka, Dong-Gi Mun, Richard K. Kandasamy, Akhilesh Pandey

**Affiliations:** 1 Department of Laboratory Medicine and Pathology, Mayo Clinic, Rochester, Minnesota, USA; 2 Department of Biochemistry and Molecular Biology, Mayo Clinic, Rochester, Minnesota, USA; 3 Proteomics Core, Mayo Clinic, Rochester, Minnesota, USA; 4 Manipal Academy of Higher Education, Manipal, Karnataka, India; 5 Center for Individualized Medicine, Mayo Clinic, Rochester, Minnesota, USA

**Keywords:** post-translational modifications, nitrotyrosine, oxidative stress, peroxynitrite, protein kinase, immunoaffinity

## Abstract

Nitrotyrosine, or 3-nitrotyrosine, is an oxidative post-translational modification induced by reactive nitrogen species. Although nitrotyrosine is considered a marker of oxidative stress and has been associated with inflammation, neurodegeneration, cardiovascular disease, and cancer, identification of nitrotyrosine-modified proteins remains challenging owing to its low stoichiometric levels in biological samples. To facilitate a comprehensive analysis of proteins and peptides containing nitrotyrosine, we optimized an immunoprecipitation-based enrichment workflow using a cell line model. The identification of proteins and peptides containing nitrotyrosine residues was carried out after peroxynitrite treatment of cell lysates, which generated modified nitrotyrosine residues on susceptible sites on proteins. We evaluated the efficacy of enriching nitrotyrosine-modified proteins and peptides by employing four different commercially available monoclonal antibodies directed against nitrotyrosine. LC-MS/MS analysis resulted in the identification of 1377 and 1624 nitrotyrosine-containing peptides from protein- and peptide-based enrichment experiments, respectively. Although the yield of nitrotyrosine-containing peptides was higher in experiments where peptides rather than proteins were enriched, we found a substantial proportion (37–65%) of identified nitrotyrosine-containing peptides contained nitrotyrosine at the N-terminus. However, in protein-based immunoprecipitation <9% of nitrotyrosine-containing peptides had nitrotyrosine modification at the N-terminus of the peptide. Overall, our study resulted in the identification of 2603 nitrotyrosine-containing peptides of which >2000 have not previously been reported. We synthesized 101 novel nitrotyrosine-containing peptides identified in our analysis and analyzed them by LC-MS/MS to validate our findings. We have confirmed the validity of 70% of these peptides, as they demonstrated a similarity score exceeding 0.7 when compared to peptides identified through experimental methods. Finally, we also validated the presence of nitrotyrosine modification on PKM and EF2 proteins in peroxynitrite-treated samples by immunoblot analysis. The large catalog presented in this study along with the workflow should facilitate the investigation of nitrotyrosine as an oxidative modification in a variety of settings in greater detail.

## Highlights

• Evaluated four monoclonal antibodies for enriching nitrotyrosine proteins/peptides.
• Optimized immunoprecipitation workflow to analyze nitrotyrosine modification.
• Identified over 2600 nitrotyrosine-containing peptides using LC-MS/MS analysis.
• Validated a subset using synthetic nitrotyrosine peptides and immunoblot analysis.

## Graphical Abstract

**Figure:**
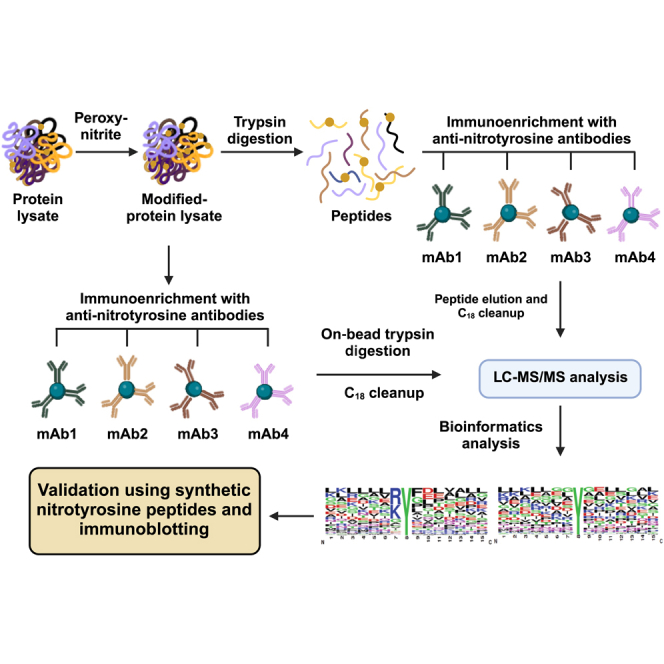

## In Brief

We systematically evaluated the efficacy of four monoclonal antibodies for enriching nitrotyrosine-containing proteins and peptides from a peroxynitrite-treated cell lysate and identified over 2600 nitrotyrosine peptides by LC-MS/MS analysis. We further evaluated 101 nitrotyrosine-containing peptides that were identified by generating synthetic peptides for comparison with experimentally observed MS/MS spectra. In addition to the large catalog of nitrotyrosine-containing proteins and peptides, this study provides valuable insights into nitrotyrosine modifications laying the groundwork for future functional studies.

Post-translational modifications (PTMs) of proteins play significant roles in altering the structure and dynamics of protein function and cellular localization (1). More than 300 PTMs have been described although only a few have been studied extensively (2). Nitration of tyrosine residues is an irreversible PTM generated from the reaction of reactive nitrogen species with proteins during oxidative stress conditions (3). Biochemically, reactive nitrogen species including peroxynitrite (ONOO^−^) and nitrogen dioxide, formed by the reaction of superoxide radicals with nitric oxide (NO) induce the formation of 3-nitrotyrosine modification (4, 5). Since tyrosine nitration is a result of redox reactions, increased levels of nitrotyrosine are considered a marker for oxidative/nitrosative stress (3, 6, 7). In addition, levels of protein tyrosine nitration have been implicated in the pathogenesis of several diseases including cancer, neurodegenerative disorders, and age-related pathologies (3, 8). For instance, nitration of tyrosine residues in tau protein at positions 18, 29, and 197 is correlated with the progression of Alzheimer’s disease and other neurodegenerative disorders (3). Nitration on tyrosine residues could also disrupt the phosphorylation status of proteins affecting downstream signaling networks (9, 10). Experimentally validated nitrated sites have been reported to be well conserved across mammals, implicating tyrosine nitration to be a non-random process regulating specific molecular processes (11, 12). Thus, understanding tyrosine nitration modification at a global level and identifying nitrated proteins should help shed light on potentially novel molecular processes that are dysregulated in disease pathology.

Mass spectrometry has become an indispensable analytical tool for in-depth proteomic analysis, especially post-translational modifications. However, because mass spectrometry-based analysis in most workflows is biased toward highly abundant species, identification of post-translationally modified peptides is challenging due to their low stoichiometric levels in cells and tissues. Thus, enrichment of such modified peptides either using immunoaffinity or chemical approaches is necessitated for any global analysis of PTMs. Unlike other PTMs such as phosphorylation, glycosylation, and ubiquitination, enrichment methods for unbiased identification of nitrated peptides are limited. One of the approaches to studying nitrated peptides involves the chemical derivatization of nitrated residue to sulfhydryl groups followed by enrichment using thiopropyl sepharose beads (13). Immunoaffinity-based methods have also been used to study nitrotyrosine modification, and recently, a study showed enrichment of nitrotyrosine-containing peptides using a monoclonal antibody specific to nitrotyrosine followed by LC-MS/MS analysis and reported identification of 1881 nitrated sites from whole cell lysates (14). However, because there are several commercially available antibodies that could be employed to enrich nitrotyrosine peptides, we performed a systematic evaluation using four different commercially available antibodies for protein as well as peptide-level enrichment analysis.

In this study, we developed workflows for protein and peptide-level enrichment for cataloging tyrosine nitrated peptides using multiple commercially available monoclonal antibodies specific for tyrosine nitrated residue resulting in the identification of >2600 tyrosine nitrated sites from a multiple myeloma cell line model. We further validated over 70 novel tyrosine nitrated peptides using synthetic peptide standards, whose MS/MS spectra showed a similarity score of more than 0.7 when compared with experimentally identified peptides. Our study provides insights into unique and shared specificities of commercially available antibodies and how it could impact the biological interpretation of global profiling datasets.

## Experimental Procedures

### Experimental Design and Statistical Rationale

Our study focused on examining proteins and peptides modified with nitrotyrosine, employing four different anti-nitrotyrosine antibodies. For this purpose, cell lysates from the MM.1S cell line was treated with peroxynitrite to induce nitration of tyrosine residues. Immunoprecipitation techniques were then utilized at both the protein and peptide levels. To account for variability across various stages of sample preparation and LC-MS/MS measurement, the immunoprecipitation experiments were performed with each antibody in triplicate. All nitrotyrosine-containing peptides identified were reported. For reporting the gene ontology and pathway analysis of nitrotyrosine-modified proteins, a cutoff of adjusted *p* value (FDR with Benjamini-Hochberg method) of 0.05 was implemented.

### Cell Culture, Lysis of Cells, and Preparation of Samples

The human multiple myeloma MM.1S cell line was cultured in RPMI-1640 media (Gibco #11875–093) supplemented with 10% fetal bovine serum (Gibco #10437–028) and maintained at 37°C with 5% CO_2_. Cell counts and cell viability were assessed routinely using Countess II (Invitrogen) with trypan blue staining. The cells were pelleted at 600*g* for 5 min and washed three times with phosphate buffer saline (PBS). Cell pellets were resuspended in modified radioimmunoprecipitation assay lysis buffer (mRIPA) containing 50 mM Tris-HCl pH 7.4, 1% NP-40, 0.25% sodium deoxycholate and 150 mM NaCl with protease and phosphatase inhibitors (Pierce # 88669) followed by three cycles of sonication. Cell lysates were then centrifuged at 12,000*g* for 10 min at 4 ˚C. Supernatants containing proteins were transferred to fresh tubes and protein estimation was done using bicinchoninic acid (BCA) protein assays (Pierce BCA Protein Assay Kit #23225). 40 mg of protein lysate was treated with 20 mM peroxynitrite (Sigma-Aldrich #20–107) for 20 min on ice. Since peroxynitrite makes lysate alkaline, the pH was adjusted with 1M HCl to 8 after this incubation step. To confirm the modification of tyrosine residues to nitrotyrosine due to peroxynitrite exposure, a western blotting experiment was performed. For this, 20 μg protein from untreated and peroxynitrite-treated samples was resolved on SDS-PAGE gel, transferred to nitrocellulose membrane, and probed with monoclonal antinitrotyrosine antibody (Abcam # ab110282).

### Affinity Enrichment of Nitrotyrosine-Modified Proteins

To enrich nitrotyrosine-containing proteins from peroxynitrite-treated samples, four monoclonal antibodies specific for nitrotyrosine from different vendors were selected, including Abcam (#ab110282), Novus Biologicals (#NB110–96877), Invitrogen (#LF-MA0225), and Santa Cruz Biotechnology (#SC32757). All the immunoenrichment experiments pertaining to each antibody were carried out in triplicate. In each experiment, 3 mg of peroxynitrite-treated lysates were incubated with 5 μg anti-nitrotyrosine antibody at 4 °C for 5 h with gentle rotation. Protein G agarose beads (Pierce # 20397) were first washed with cold 1X PBS four times and resuspended in mRIPA buffer and then added to the samples. After overnight incubation, the samples were centrifuged at 1,000*g* for 2 min. The beads were further washed with mRIPA buffer twice followed by three washes with 1X PBS. Proteins bound to beads were then subjected to on-bead trypsin digestion.

### On-Bead Trypsin Digestion of Antibody-Captured Proteins

Urea buffer (2 M urea in 100 mM TEAB, 10 mM dithiothreitol (DTT)) was added to the beads and incubated at 37 °C for 1 hour with gentle agitation. Alkylation was performed in the dark for 30 min using 40 mM iodoacetamide. To digest the proteins, 1 μg of mass spectrometry-grade trypsin (Pierce #90057) was added to each sample and incubated at 37 °C overnight. Samples were centrifuged at 1000*g* for 2 min and the supernatants were carefully transferred to fresh tubes. Peptide samples were acidified using 1% trifluoroacetic acid (TFA). Peptides were cleaned using in-house packed C_18_ stage tips (Empore C_18_ SPE Disks #98–0604–0217–3) and dried under vacuum in speed vac. The dried samples were stored at −80 °C until mass spectrometry analysis.

### Affinity Enrichment of Nitrotyrosine-Modified Peptides

For peptide-based experiments, peroxynitrite-treated protein lysates were first digested into peptides and then subjected to immunoprecipitation experiments. For this, MM.1S cell line was lysed in urea lysis buffer (8M urea in 100 mM TEAB with protease and phosphatase inhibitor) and sonicated on ice. Cell lysates were centrifuged at 12,000*g* for 10 min at 4 ˚C, and protein estimation was performed using BCA protein assay. 40 mg of proteins were treated with 20 mM peroxynitrite for 20 min on ice. Subsequently, pH of the samples was adjusted to 8 with 1M HCl. Proteins were subjected to reduction and alkylation with 10 mM DTT and 20 mM IAA, respectively. Samples were diluted with 100 mM TEAB to <2 M urea concentration for efficient trypsin digestion. Trypsin protease in the ratio of 1:20 (protease: protein) was added and incubated at 37 °C overnight. Samples were acidified with 1% TFA. Peptides were cleaned with Sep-Pak C_18_ columns and eluate was lyophilized overnight. Lyophilized peptides were resuspended in immunoaffinity buffer (PTMScan, IAP Buffer, Cell Signaling #9993), sonicated for proper solubilization, and centrifuged at 10,000*g* for 5 min. Supernatants containing the peptides were collected and incubated with the same monoclonal antibodies that were used for enriching nitrotyrosine modified proteins. Similar to protein-based immunoprecipitation experiments, 5 μg of each antibody was added to 3 mg peptide and incubated for 5 h at 4 °C. Protein G agarose beads were then added and incubated overnight at 4°C with gentle rotation. The samples were centrifuged at 1,000*g* for 2 min and washed with IAP buffer twice followed by three washes with MS-grade water. To elute the peptides bound to beads, 50 μl of 0.15% TFA was added to the beads and incubated at room temperature for 10 min with gentle mixing. Samples were centrifuged at 1,000*g* for 2 min and supernatants containing peptides were collected. The elution step was repeated twice and peptides were cleaned using C_18_ stage tips. Desalted peptides were dried under vacuum in speed vac and stored at −80 °C until further analysis.

### LC-MS/MS Analysis and Database Searching

All peptide samples were analyzed on an Orbitrap Eclipse Tribrid mass spectrometer (Thermo Scientific) interfaced with a Dionex RSLC3000 liquid chromatography system (Thermo Scientific). Peptides were suspended in solvent A (0.1% formic acid in water) and loaded on a trap column (PepMap C_18_ 2 cm × 100 μm, 100 Å) and resolved on a separation column (PepMap 50 cm × 75 μm, C_18_ 1.9 μm, 100 Å, Thermo Scientific) at a flow rate of 300 nl/min using a gradient of 5 to 32% solvent B (0.1% formic acid in acetonitrile) for 120 min in a total run time of 150 min. Mass spectrometer was operated in data-dependent mode with a cycle time of 2 s. Survey MS scan was acquired in Orbitrap mass analyzer with 120,000 resolution, 2.5 × 10^6^ AGC target value, and 50 ms of maximum ion injection time. Precursor ions with charge states 2 to 7 were isolated using an isolation width of 1.2 m/z for MS/MS and fragmented with 30% higher-energy collisional dissociation (HCD) energy. Fragmented ions were analyzed in orbitrap mass analyzer with a resolution of 30,000. Automatic gain control (AGC) target value and maximum ion injection time for MS/MS scans were set as 2 × 10^5^ ions and 54 ms, respectively. Dynamic exclusion was enabled for 30 s. Raw mass spectrometry data were analyzed using SEQUEST search engine against UniProt human database (20,541 entries, released in February 2021) in Proteome Discoverer 2.5 software. Trypsin was specified as an enzyme for in silico digestion with a maximum missed cleavage of 2. Precursor ion tolerance of 10 ppm and fragment ion tolerance of 0.05 Da were used for database searching. Oxidation on methionine (+15.995 Da), nitration on tyrosine (+44.985 Da), and acetylation on protein N-termini (+42.011 Da) were set as dynamic modifications; carbamidomethylation of cysteine (+57.021 Da) was set as a static modification. False discovery rate was set as 1% both at peptide and protein levels.

### Gene Ontology and Pathway Analysis of Enriched Proteins

Proteins identified with nitrotyrosine modification were further characterized for their subcellular localization, molecular function, and pathway networks using database packages in R including “GO_Molecular_Function_2023”, “GO_Cellular_component_2023” and “KEGG_2021_Human” (15). We used “enrichR” database package available at https://cran.rproject.org/web/packages/enrichR/index.html for this analysis. An FDR-adjusted *p*-value <0.01 was used to determine significant enrichment. Protein hits for the terms enriched in each category were then compared for the peptides identified in the enriched pool by the four antibodies at peptide and protein level enrichment.

### Spectral Correlation Between Synthetic and Experimentally Identified Nitrotyrosine Peptides

To confirm the identification of nitrotyrosine peptides in our study, we selected around 110 nitrotyrosine-containing peptides identified in pull-down experiments. The peptides were synthesized using standard fluorenylmethoxycarbonyl protecting group (Fmoc) chemistry on a MultiPep RSi (CEM Corp.) multiple peptide synthesizer retrofitted with a 384-well filter plate. Peptides were synthesized by incorporating Fmoc-3-nitro-L-Tyrosine (Chem-Impex International) at positions identified in experimental data. The peptides were cleaved for 2.5 h at room temperature using a cleavage cocktail of trifluoroacetic acid, water, triisopropyl silane, and 3,6-Dioxa-1,8-octanedithiol (92.5/2.5/2.5/2.5 v/v/v/v). Peptide solutions were transferred to 96-deep well plates, and then precipitated and washed with cold methyl tert-butyl ether (MTBE). Peptide pellets were dried overnight in a hood, then dissolved in 20% acetonitrile. Peptides were pooled in equimolar concentration, dried, and resuspended in 0.1% formic acid. MS/MS spectra of synthetic peptides were acquired on Orbitrap Eclipse mass spectrometer using the same acquisition parameters that were used for experimental samples.

MS/MS spectra from these synthetic peptides were overlaid with the corresponding MS/MS spectra obtained from experimental samples to calculate the cosine spectral similarity score using an R script. The spectral similarity analysis was performed using the R package “OrgMassSpecR” available at https://cran.r-project.org/web/packages/OrgMassSpecR/index.html. For the purposes of the comparison, only peaks over 2% of the base peak were considered. A tolerance of 0.01 Da was used for matching peaks. The spectral similarity score between aligned vectors of spectral peak intensities u and v were calculated using the following equation:

$$\cos\;\theta =\frac{\sum_{i=1}^{n}u_{i}\;.\;v_{i}}{\sqrt{\sum_{i=1}^{n}u_{i}^{2}}\;.\;\sqrt{\sum_{i=1}^{n}v_{i}^{2}}}$$

### Western Blotting of Nitrotyrosine Modification on PKM and EF2 Proteins

MM.1S cell line was cultured in RPMI 1640 media. The cells were pelleted down and washed with PBS three times. Cells were lysed in mRIPA buffer containing protease and phosphatase inhibitors and protein estimation was done using BCA assay. Protein lysate was either left untreated or treated with peroxynitrite using the same procedure as mentioned above. Protein lysates from treated and untreated samples were subjected to immunoprecipitation using PKM2-specific monoclonal antibody (Proteintech #60268-1-Ig) or EF-2 specific monoclonal antibody (Santa Cruz Biotechnology #sc-166415). For immunoprecipitation, 2 μg of antibody was used to enrich proteins from 1 mg of lysates. Enriched proteins were resolved by SDS-PAGE and assayed by western blotting using an anti-nitrotyrosine antibody (Abcam # ab110282). Further, the blots were reprobed with corresponding anti-protein antibodies.

## Results and Discussion

Immunoaffinity-based approaches, employing antibodies raised against specific modifications to enrich corresponding post-translationally modified peptides or proteins have become popular for studying different PTMs. Although antibodies specifically directed against nitrotyrosine are available, there are no reports describing the evaluation of these antibodies to enrich nitrotyrosine-modified proteins or peptides. To address this, we employed protein-based and peptide-based immuno-enrichment approaches to explore the identification of nitrotyrosine-modified proteins and peptides.

### Immunoaffinity Capture of Proteins and Peptides Containing Nitrotyrosine Modifications

**Fig. 1:**
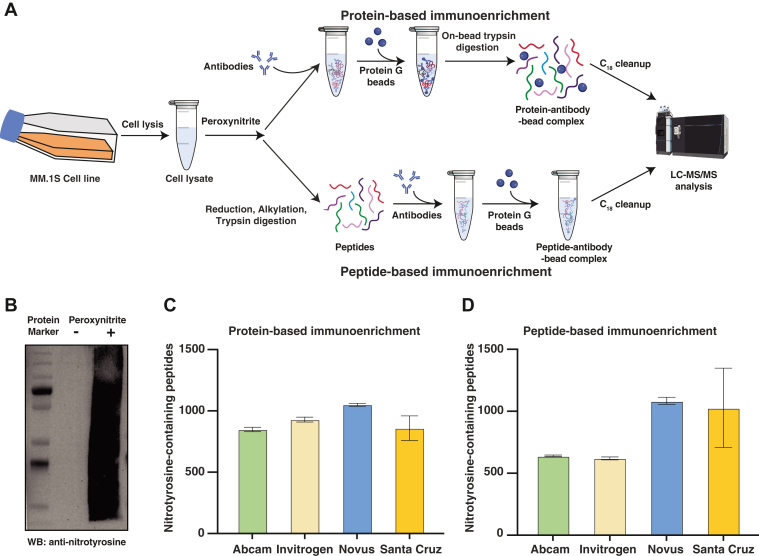
**Nitrotyrosine-containing proteins and peptides enriched using antibodies.***A*, a schematic of the workflow employed for enriching nitrotyrosine-modified proteins and peptides. Protein lysates from MM.1S cell line were treated with peroxynitrite followed by enrichment of nitrotyrosine-modified proteins using four different monoclonal antibodies (in triplicate). Protein G beads were used to capture the nitrotyrosine-modified proteins that were digested using on-bead trypsin digestion protocol as indicated. Tryptic peptides were cleaned and analyzed by LC-MS/MS. Alternatively, for peptide-based immunoprecipitation of peroxynitrite-treated lysates, proteins were first digested into peptides followed by immunocapture of nitrotyrosine-containing peptides using the four antibodies prior to clean up and LC-MS/MS (in triplicate). *B*, Western blot showing the effect of peroxynitrite treatment. Anti-nitrotyrosine antibody (Abcam) was used to confirm the nitrotyrosine-modified proteins in untreated and peroxynitrite-treated lysates. The left lane depicts protein molecular weight markers and the lanes marked “-“and “+” refer to untreated or peroxynitrite-treated cell lysates. *C*, histograms showing the number of nitrotyrosine-containing peptides identified from immunoaffinity experiments carried at the protein level using four different antibodies. *D*, number of nitrotyrosine-containing peptides identified from corresponding peptide-based immunoaffinity experiments conducted at the peptide level.

To perform this systematic comparison, we induced the formation of nitrated proteins by treating a myeloma cell line, MM.1S, with peroxynitrite. We performed protein- and peptide-level enrichment with four different nitrotyrosine-specific antibodies in triplicate followed by mass spectrometric analysis (Fig. 1*A*). For protein-based immunoaffinity experiments, nitrotyrosine-containing proteins were enriched first and subsequently digested into peptides whereas for peptide-based immunoaffinity experiments, proteins were first digested into peptides followed by antibody-based enrichment. We confirmed the presence of nitrated proteins after peroxynitrite treatment by western blotting with an anti-nitrotyrosine antibody (Fig. 1*B*). Mass spectrometric analysis of protein-based enrichment resulted in the identification of a total of 1377 nitrotyrosine-containing peptides (supplemental Table S1*A*). These nitrotyrosine-containing peptides were derived from 571 proteins. Similarly, peptide-based enrichment led to the identification of a total of 1624 nitrotyrosine-containing peptides (derived from 977 proteins) (supplemental Table S1*B*). Antibody from Novus provided the most reproducible and higher number of nitrotyrosine-containing peptides compared to other antibodies (Fig. 1, *C* and *D*) (supplemental Fig. S1).

The largest number of nitrotyrosine-containing peptides reported thus far is 1559 from a peroxynitrite-treated lung cancer cell line in which the anti-nitrotyrosine antibody from Santa Cruz was used to enrich modified peptides (14). Besides immunoaffinity methods, chemical derivatization techniques like solid-phase active ester reagent (SPEAR) and aromatic nitration site identification (ANSID) have also been employed to capture nitrotyrosine-containing peptides (16, 17). However, it should be noted that these methods tend to yield fewer nitrotyrosine-containing peptides compared to immunoaffinity enrichment approaches. In contrast, our study resulted in the identification of a total of 2603 nitrotyrosine-containing peptides (derived from 1223 proteins) of which >2000 have not been reported previously (supplemental Table S1*C*).

### Functional Analysis of Nitrotyrosine Containing Proteins

**Fig. 2:**
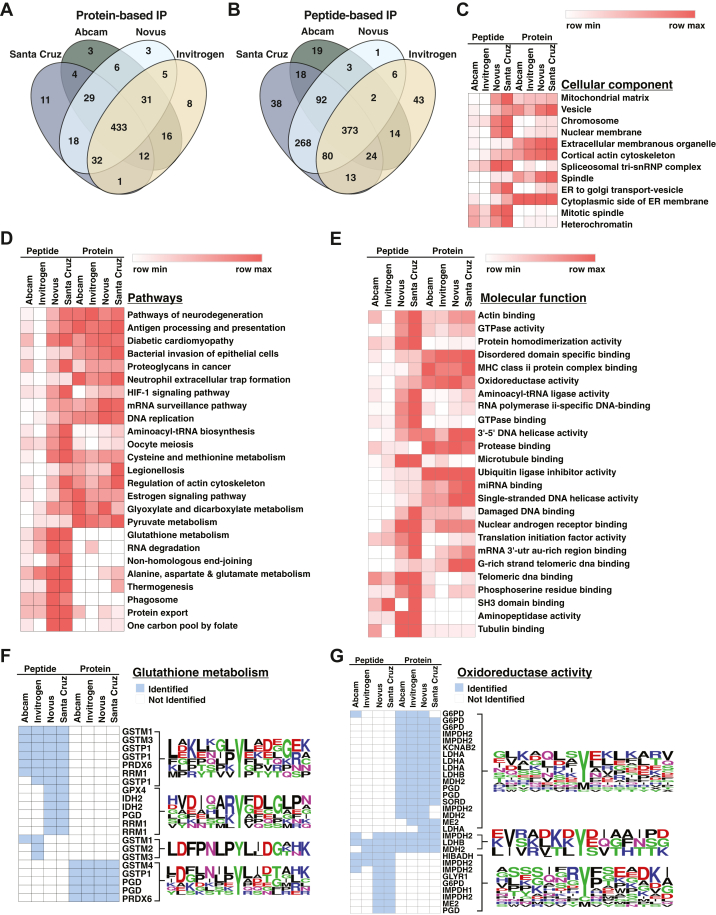
**Distinct profiles of protein and peptide level enrichment strategies.***A*, Venn diagram showing overlap of nitrotyrosine-modified proteins from protein enrichment. *B*, Venn diagram showing overlap of nitrotyrosine-modified proteins from peptide enrichment. *C*, a heatmap of cellular component analysis across the two immunoaffinity approaches using four different antibodies. *D*, a heatmap of KEGG pathway analysis across the two immunoaffinity approaches using four different antibodies. *E*, a heatmap of molecular function analysis across the two immunoaffinity approaches using four different antibodies. *F*, enrichment of nitrotyrosine peptides derived from proteins involved in glutathione metabolism. Antibodies recognize and capture a unique set of peptides when used for immunoprecipitation at protein and peptide levels. In the upper section of the figure, nitrotyrosine peptides that were enriched at the peptide level each displaying unique motif sequences. In contrast, proteins identified through protein-based immunoaffinity experiments showcase different motif sequences, as illustrated in the lower section of the figure. Additionally, it is worth noting that certain peptides were specifically identified by either one or two antibodies. *G*, Nitrotyrosine-containing peptides from proteins with oxidoreductase activity identified across experiments. In the top part of the figure, you see nitrotyrosine peptides enriched at the protein level, each with its unique pattern. At the peptide level, Santa Cruz and Novus antibodies captured some peptides uniquely as shown in the bottom part of the figure. Some peptides were enriched with any antibody irrespective of the immunoprecipitation method used as depicted in the middle part of the figure.

We performed a comparative analysis of proteins modified with nitrotyrosine among protein-based immunoprecipitation experiments. Of the 571 proteins modified with nitrotyrosine that were enriched, 433 were common to all four antibodies, indicating that the major proportion of nitrotyrosine-containing proteins were enriched by every antibody (Fig. 2*A*). In contrast, peptide-based immunoprecipitation experiments led to only 373 (out of 977) nitrotyrosine-containing proteins that were in common across all four antibodies (Fig. 2*B*). This prompted us to investigate the functional profile of nitrotyrosine-containing peptides enriched by each antibody by the two methods. Gene ontology and pathway analysis of the enriched proteins showed common biological features enriched across experiments in addition to uniquely identified biological features associated with either antibody and/or mode of immunoaffinity enrichment (supplemental Table S2). Cellular component analysis showed that the majority of nitrotyrosine-containing proteins localized to the mitochondrial matrix, vesicles, and nuclear membrane (Fig. 2*C*). Similarly, pathway analysis mapped nitrotyrosine-containing proteins to neurodegeneration, antigen processing and presentation, and HIF-1 signaling, among others (Fig. 2*D*). Nitration of proteins has been associated with neurodegenerative disorders including Alzheimer’s disease, amyotrophic lateral sclerosis, and Parkinson’s disease (18, 19).

Immunoaffinity enrichment at the peptide level resulted in the enrichment of more nitrotyrosine-modified proteins belonging to pathways including aminoacyl-tRNA biosynthesis, HIF-1 signaling, glutathione, and amino acid metabolism as compared to protein-based enrichment. Santa Cruz and Novus antibodies selectively enriched nitrotyrosine-modified peptides from proteins associated with protein export and one-carbon metabolism *via* folate. Likewise, both immunoenrichment methods effectively captured proteins involved in DNA replication, pyruvate metabolism, and antigen processing/presentation. However, the protein-based enrichment approach yielded a comparatively greater number of nitrotyrosine-modified peptides associated with these pathways. Molecular class analysis revealed that protein-based experiments were particularly proficient at enriching proteins linked to the MHC Class II protein complex and specific disordered domains, mainly related to antigen processing and presentation pathways (Fig. 2*E*). Additionally, proteins exhibiting oxidoreductase activity were more efficiently enriched through protein-based immunoprecipitation experiments. Cellular component analysis revealed most of these proteins to be localized in the mitochondrial matrix.

We observed that the profile of proteins belonging to certain pathways depended on the particular antibody used. For example, the enrichment of proteins related to glutathione metabolism varied depending on the antibody and the immunoprecipitation method used and was based on the sequence surrounding the nitrotyrosine residues (Fig. 2*F*). Glutathione is one of the essential antioxidants, playing important roles in aging and the pathogenesis of many diseases including, Alzheimer's disease, Parkinson's disease, and cancer (20). Nitration of proteins involved in glutathione metabolism could impact its antioxidant activity. Similarly, proteins annotated as oxidoreductases were enriched specifically based on motifs recognized by antibodies (Fig. 2*G*). This functional annotation of enriched pathways is important as it highlights the ability of each antibody, in conjunction with the immunoprecipitation method, to enrich somewhat distinct subsets of pathways. This analysis is essential for selecting the best approach when studying the impact of tyrosine nitration on pathways or protein classes.

### Motif Analysis Reveals Presence of Small Non-polar Amino Acids Around Tyrosine

**Fig. 3:**
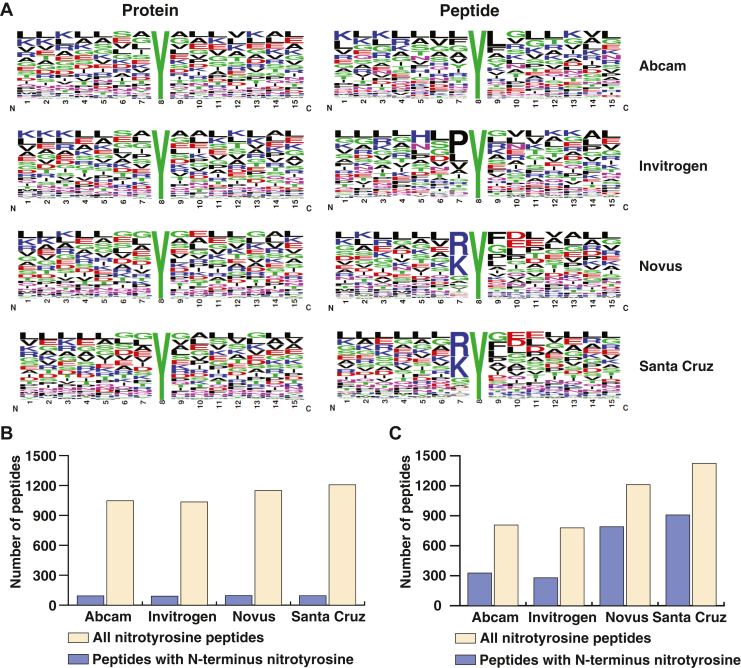
**Unique motifs in sequences surrounding nitrotyrosine from protein and peptide level enrichment strategies.***A*, sequence logo analysis of nitrotyrosine-containing peptides from protein- and peptide-based experiments with different antibodies as indicated. *B*, the number of peptides containing nitrotyrosine at the N-terminus is shown along with all nitrotyrosine-containing peptides identified from protein-based immunoaffinity experiments and (*C*) peptide-based immunoaffinity experiments.

Amino acid sequences surrounding tyrosine residues modified by nitration reaction were evaluated through motif analysis. In all scenarios, it was observed that small aliphatic non-polar amino acids formed a major core around the nitrotyrosine residues. Earlier reports also suggested the presence of hydrophobic amino acids including glycine, leucine, proline, alanine, and glutamate around tyrosine residue determining its susceptibility to getting nitrated (21, 22). In peptide-based experiments, Santa Cruz and Novus antibodies were more efficient at capturing peptides with nitrotyrosine at the N-terminus than at any other position within the peptide. This was reflected in the sequence motif analysis where nitrotyrosine residue was preceded by lysine or arginine amino acids (Fig. 3*A* right panel). This pattern of enriching peptides with N-terminal nitrotyrosine was observed earlier where peptide-based immunoaffinity enrichment was performed solely using Santa Cruz anti-nitrotyrosine antibody (14). In protein-based experiments, the proportion of N-terminus nitrotyrosine ranged from 8% to 9% across all antibodies (Fig. 3*B*). However, the percentage of N-terminus nitrotyrosine ranged from 35% to 65% in peptide-based experiments (Fig. 3*C*). This phenomenon of enriching peptides with N-terminus nitrotyrosine in peptide-based enrichment could be because of its easy access compared to nitrotyrosine present at any other position in the peptide sequence or because of some unique feature of the immunogen that was used to generate these antibodies. This analysis shows that both protein and peptide-based immunoaffinity approaches should be considered together to ensure complete enrichment of all nitrotyrosine proteins and peptides.

### Validation of Nitrotyrosine Peptides Using Synthetic Peptides

**Fig. 4:**
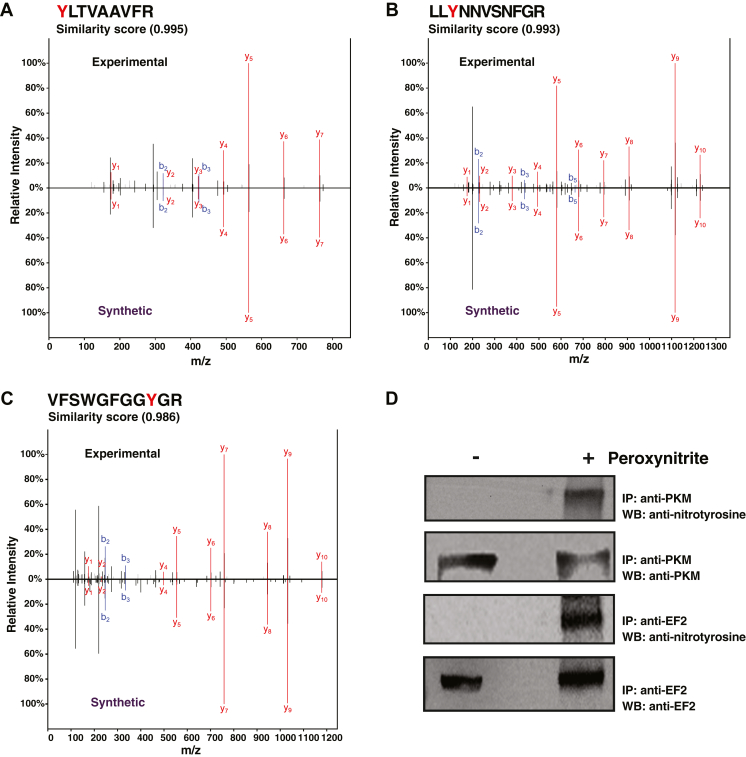
**Validation of nitrotyrosine-containing peptides using synthetic peptides and immunoblotting**. MS/MS spectra from experimental data were overlayed with the MS/MS spectra of synthetic peptides. Representative MS/MS spectra are shown for (*A*) YLTVAAVFR (*TUBB*) (*B*) LLYNNVSNFGR (*CLTC*) (*C*) VFSWGFGGYGR (*RCC2*). Top panel in each figure is from experimental data and the bottom panel from the corresponding synthetic peptide. The similarity score between the two spectra is indicated (*D*) Validation of nitrotyrosine-containing proteins using western blotting. Immunoprecipitation was performed using anti-PKM and anti-EF2 antibodies from peroxynitrite-treated and untreated MM.1S cell lysate. Immunoprecipitated sample was resolved on SDS-PAGE gel and analyzed by western blotting. Immunoblotting was first done using an anti-nitrotyrosine antibody and then reprobing was done using corresponding anti-protein antibodies (anti-PKM and anti-EF2).

To evaluate the reliability of our approach, we decided to perform validation of the identified nitrotyrosine peptides, especially as a majority of them have not been previously identified. To accomplish this, we selected 103 novel nitrotyrosine peptide sequences from the experimental data for generating synthetic peptides. These synthetic nitrotyrosine peptides were analyzed by LC-MS/MS using the same data acquisition settings as used for experimental samples. MS/MS spectra of synthetic nitrotyrosine peptides were compared to corresponding spectra obtained from enrichment experiments. Spectral similarity showed ∼70% peptides with a cosine similarity score of >0.7 (supplemental Fig. S2). Mirror images obtained after overlaying MS/MS spectra of three representative nitrotyrosine peptides from experiments and synthetic peptides along with cosine similarity score are shown in Figure 4, *A*–*C*. MS2 fragment ions (y and b) that showed matches between experimental and synthetic peptides are annotated (supplemental Fig. S3).

To further evaluate the efficiency of immunoaffinity approaches in enriching nitrotyrosine peptides, another layer of validation experiment was performed on two selected candidates. Pyruvate kinase (PKM) and elongation factor (EF2) proteins were among the topmost nitrated proteins identified in the current study. Interestingly, most of the tyrosine residues modified due to nitration after peroxynitrite treatment in PKM and EF2 have been reported earlier as phosphorylation sites (23, 24, 25). Immunoenrichment experiments were carried out in both treated and untreated MM.1S cell lysates using anti-PKM and anti-EF2 antibodies. Immunoblotting of enriched samples with anti-nitrotyrosine antibodies shows the bands corresponding to PKM and EF2 proteins in their respective blots only in treated lysates (Fig. 4*D*). Further, reprobing with anti-PKM and anti-EF2 antibodies showed protein bands corresponding to respective proteins in both treated and untreated samples. These results confirm the applicability of immunoaffinity enrichment strategies in identifying nitrotyrosine-modified proteins and peptides.

## Conclusion

The immunoenrichment experiments demonstrated in this study resulted in the identification of >2600 nitrotyrosine peptides representing the largest catalog of nitrotyrosine peptides identified to date. A large proportion of the nitrotyrosine sites in the catalog are novel and we also synthesized 101 among these novel nitrotyrosine-containing peptides to validate our findings. When we compared the spectra of these peptides to those identified in experimental samples, 71 (∼70%) of them exhibited a similarity score exceeding 0.7. Although our study showed that peptide-based immunoaffinity approach enriches more nitrotyrosine-modified peptides compared to protein-based immunoaffinity enrichment, we recommend considering both protein and peptide-based immunoaffinity approaches to enable an unbiased in-depth analysis of the nitrotyrosine-modified proteome. Our study highlights the scope of relatively unexplored nitrotyrosine modification on a global scale underscoring the need for future investigations aimed at comprehensively elucidating its role in pathological mechanisms.

## Data Availability

The mass spectrometry proteomics data have been deposited to the ProteomeXchange Consortium *via* the PRIDE (26) partner repository with the dataset identifier PXD048204".

## Supplemental Data

This article contains supplemental data.

## Conflict of interest

All the authors declare that they have no conflict of interest with the contents of this article.
