# Supplementary material for: Exploration of Nitrotyrosine-Containing Proteins and Peptides by Antibody-Based Enrichment Strategies
Source: Mol Cell Proteomics. 2024 Feb 10;23(3):100733. doi: 10.1016/j.mcpro.2024.100733 (PMC10950883; doi:10.1016/j.mcpro.2024.100733)
Supplement: Supplementary Figures [file mmc1.pdf]

Supplementary Figure 1

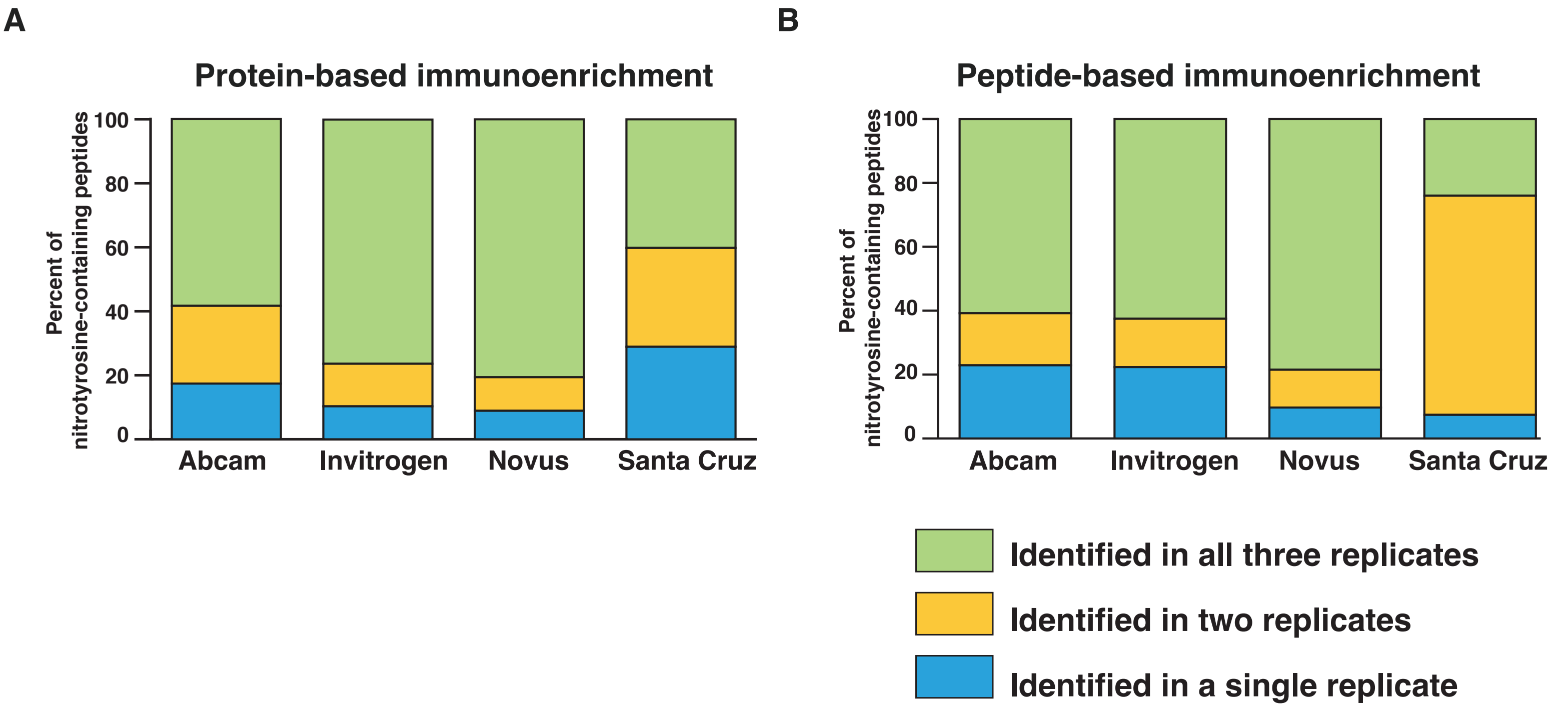

Supplementary Figure 2

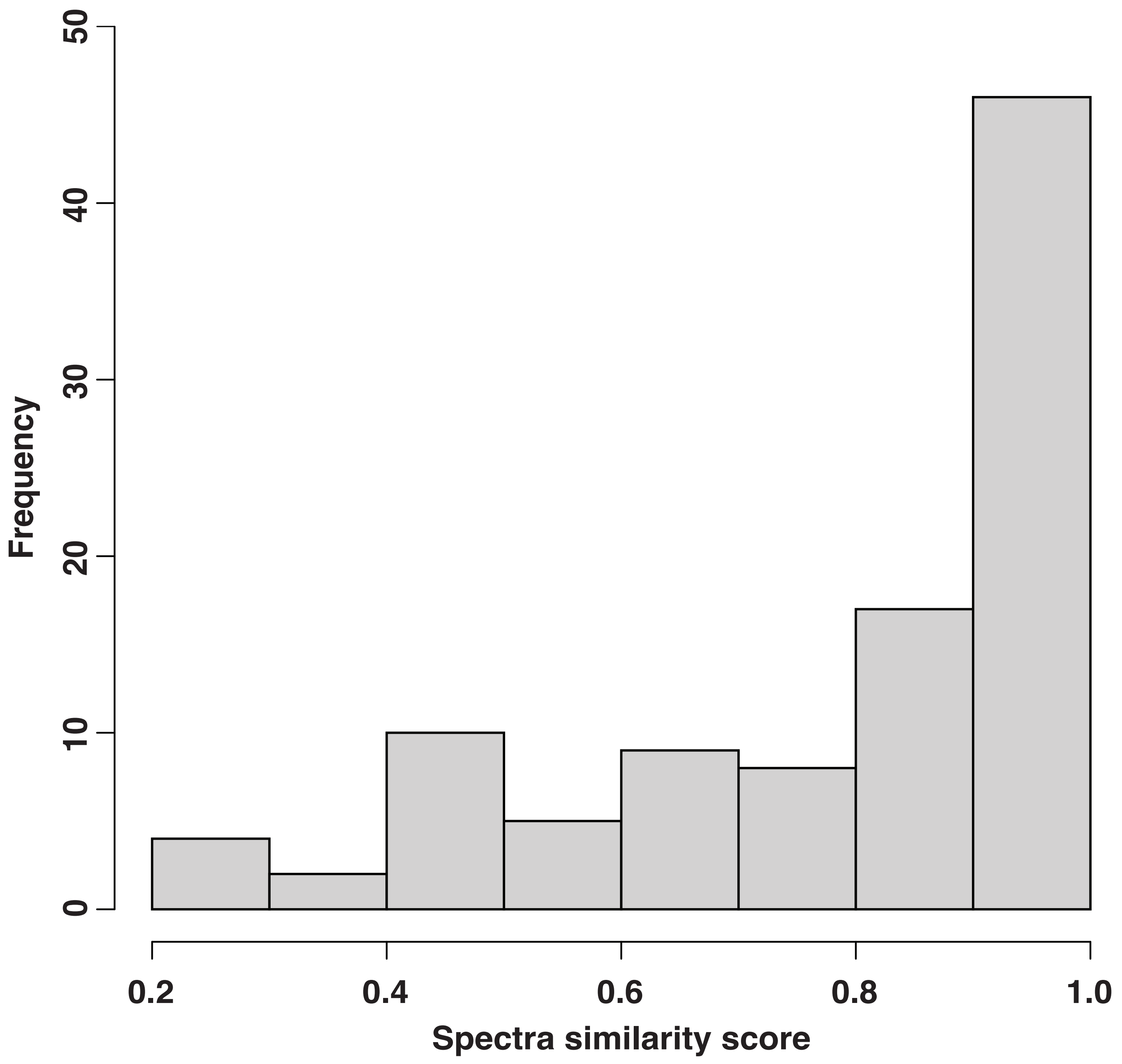

Supplementary Figure 3

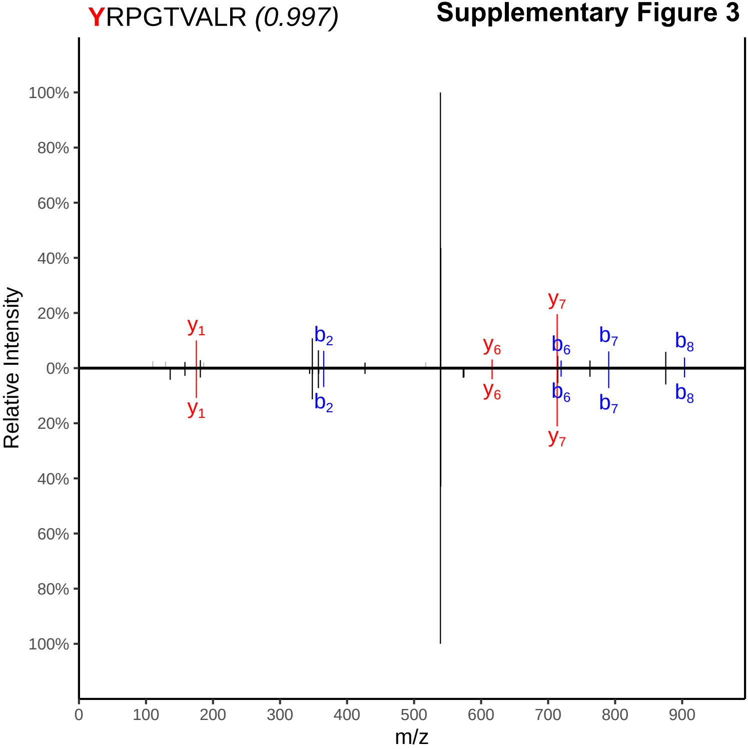

YLTVAAVFR *(0.995)*

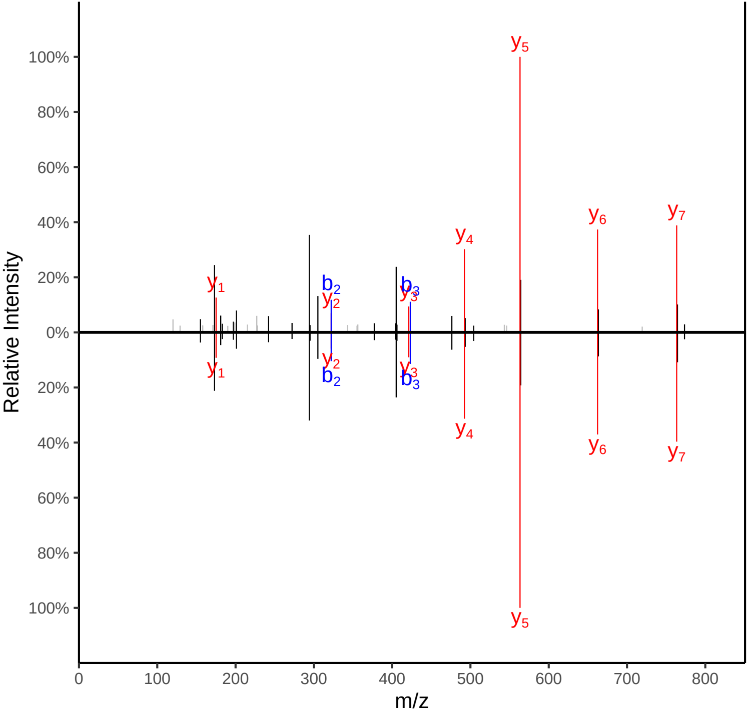

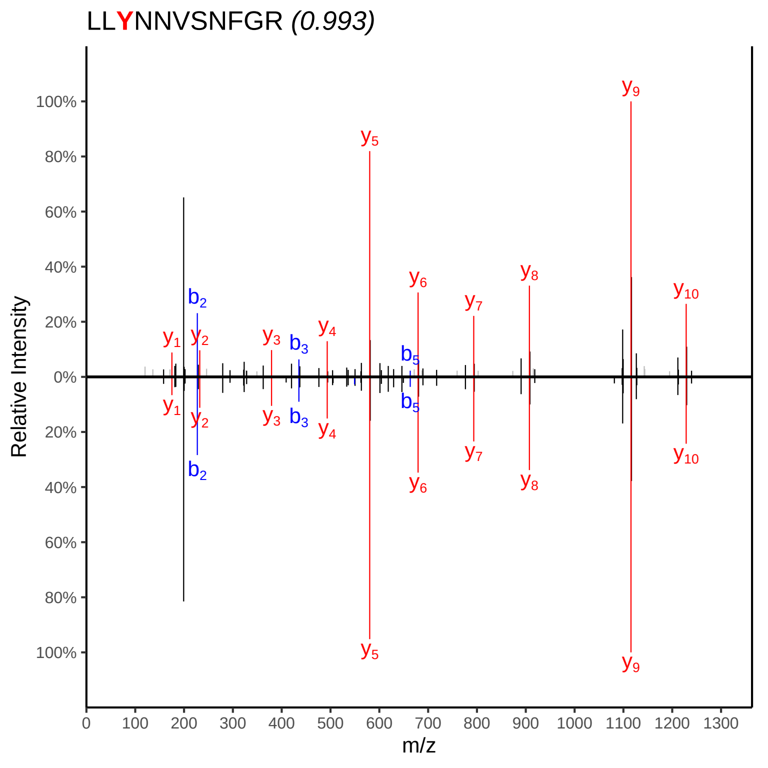

LLYNNVSNFGR (0.993)
Relative Intensity
m/z
y1
y2
y3
y4
y5
y6
y7
y8
y9
y10
b2
b3
b5

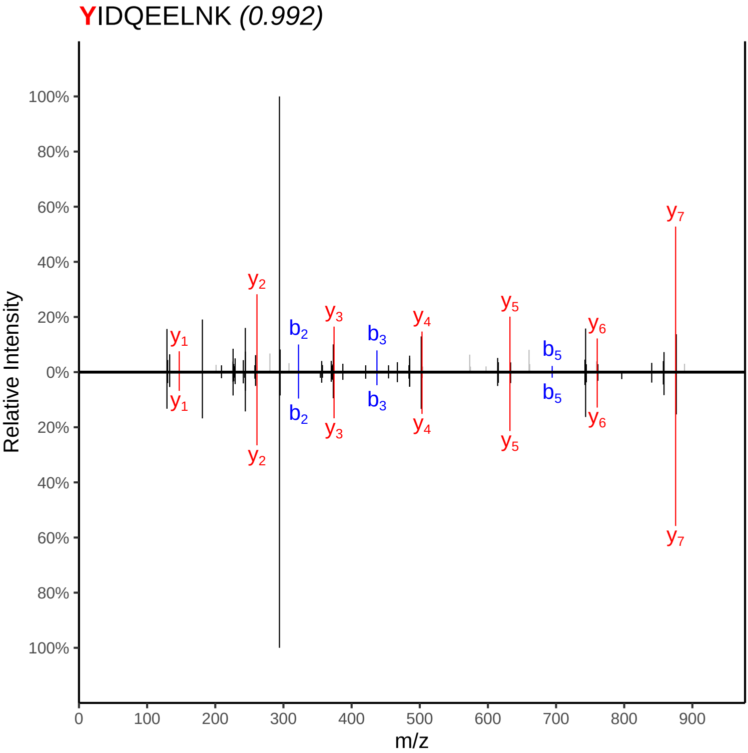

YIDQEELNK (0.992)
Relative Intensity
100%
80%
60%
40%
20%
0%
20%
40%
60%
80%
100%
m/z
0
100
200
300
400
500
600
700
800
900
y1
y2
b2
y3
b3
y4
y5
b5
y6
y7

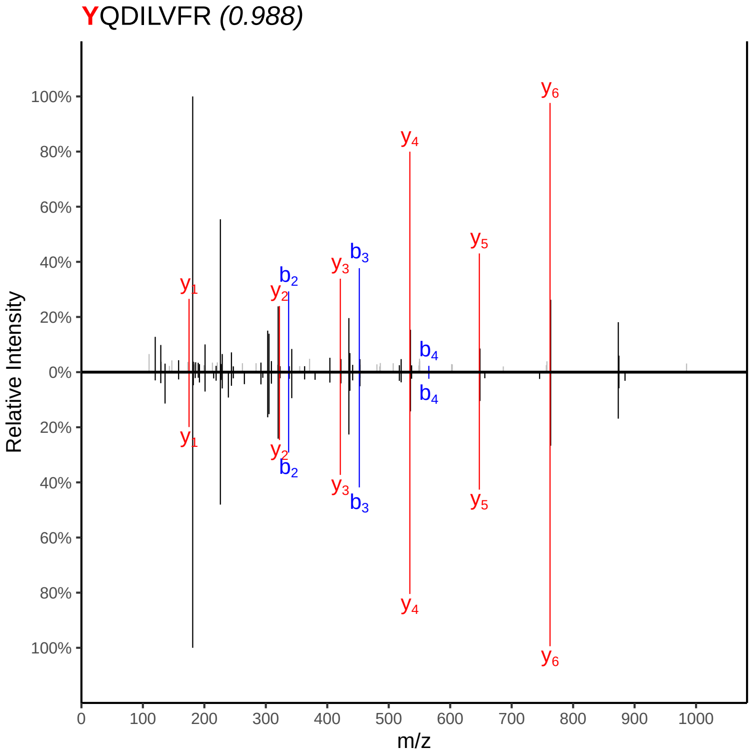
YQDILVFR (0.988)
Relative Intensity
m/z
100%
80%
60%
40%
20%
0%
20%
40%
60%
80%
100%
0
100
200
300
400
500
600
700
800
900
1000
y1
y2
b2
y3
b3
y4
b4
y5
y6
y1
y2
b2
y3
b3
y4
b4
y5
y6

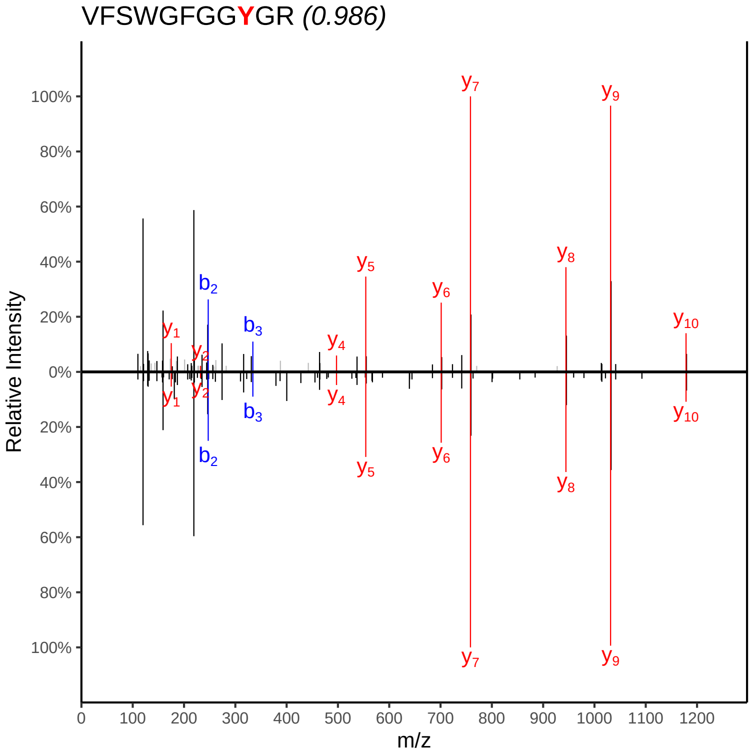

VFSWGFGGYGR (0.986)
Relative Intensity
m/z
100%
80%
60%
40%
20%
0%
20%
40%
60%
80%
100%
0
100
200
300
400
500
600
700
800
900
1000
1100
1200
y1
y2
b2
b3
y4
y5
y6
y7
y8
y9
y10

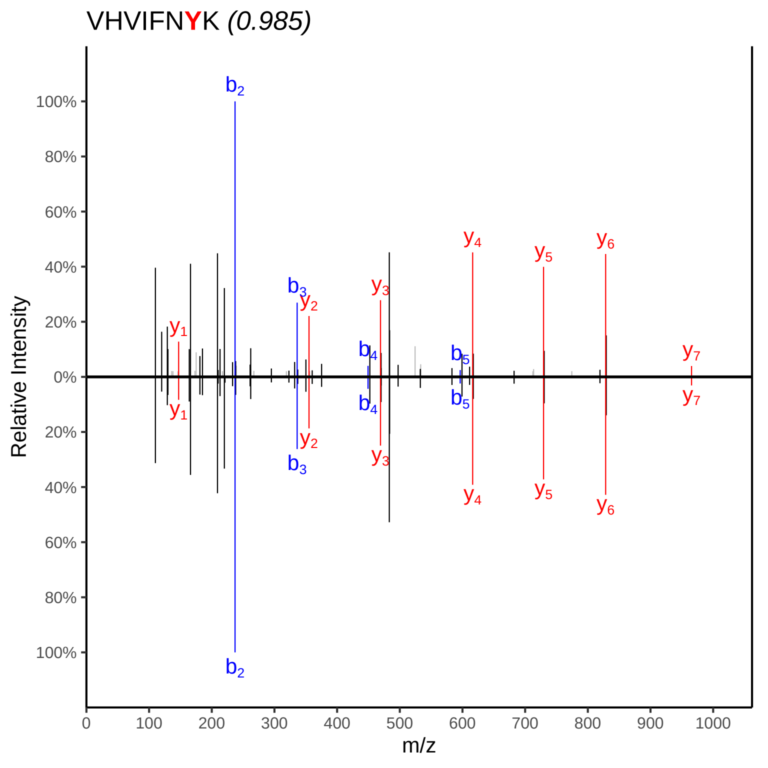
VHVIFNYK (0.985)
Relative Intensity
m/z
100%
80%
60%
40%
20%
0%
20%
40%
60%
80%
100%
0
100
200
300
400
500
600
700
800
900
1000
b2
b3
b4
b5
y1
y2
y3
y4
y5
y6
y7

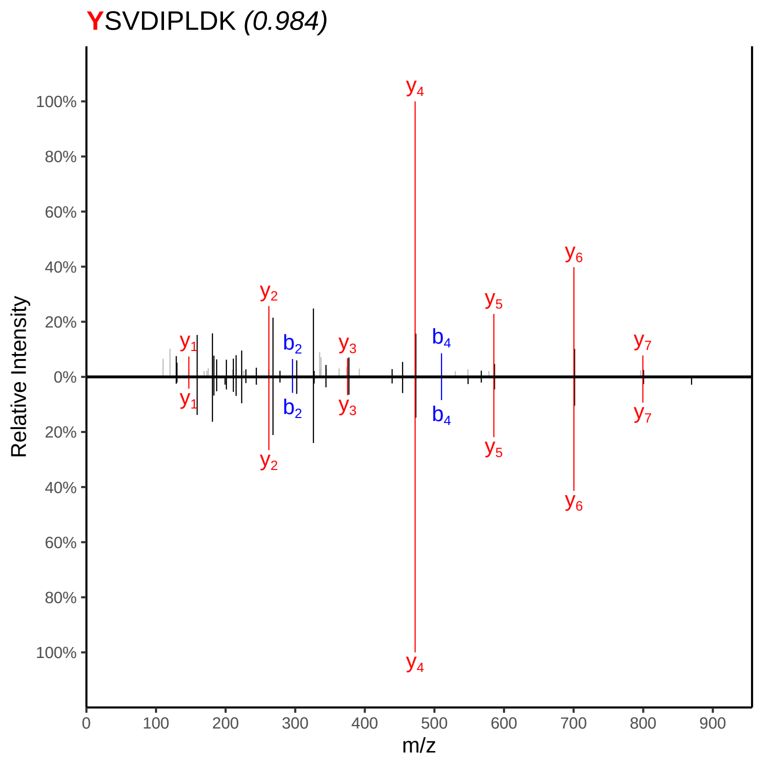

YSVDIPLDK (0.984)
Relative Intensity
m/z
y1
y2
b2
y3
y4
b4
y5
y6
y7

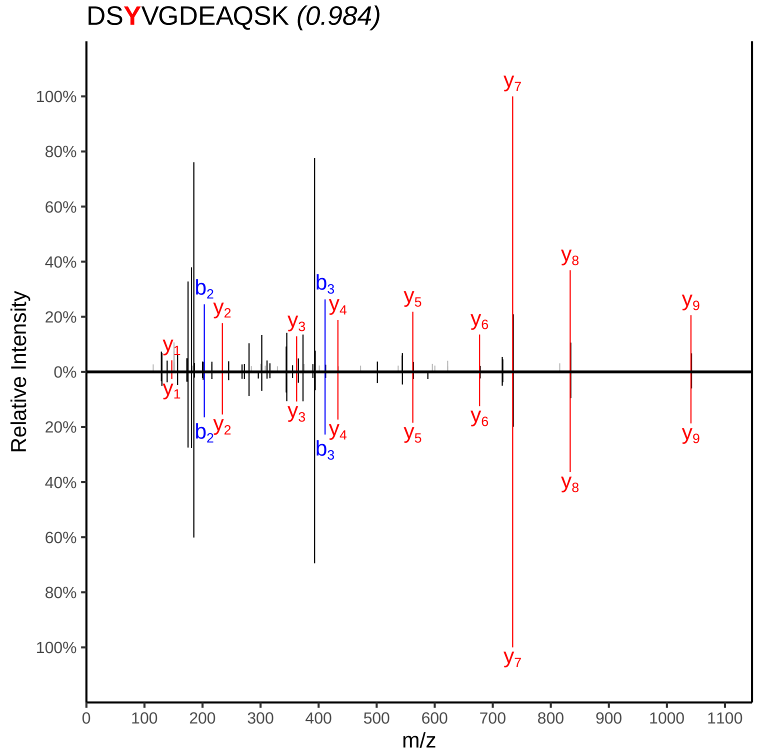

DSYVGDEAQSK (0.984)
Relative Intensity
m/z
100%
80%
60%
40%
20%
0%
20%
40%
60%
80%
100%
0
100
200
300
400
500
600
700
800
900
1000
1100
y1
b2
y2
y3
b3
y4
y5
y6
y7
y8
y9

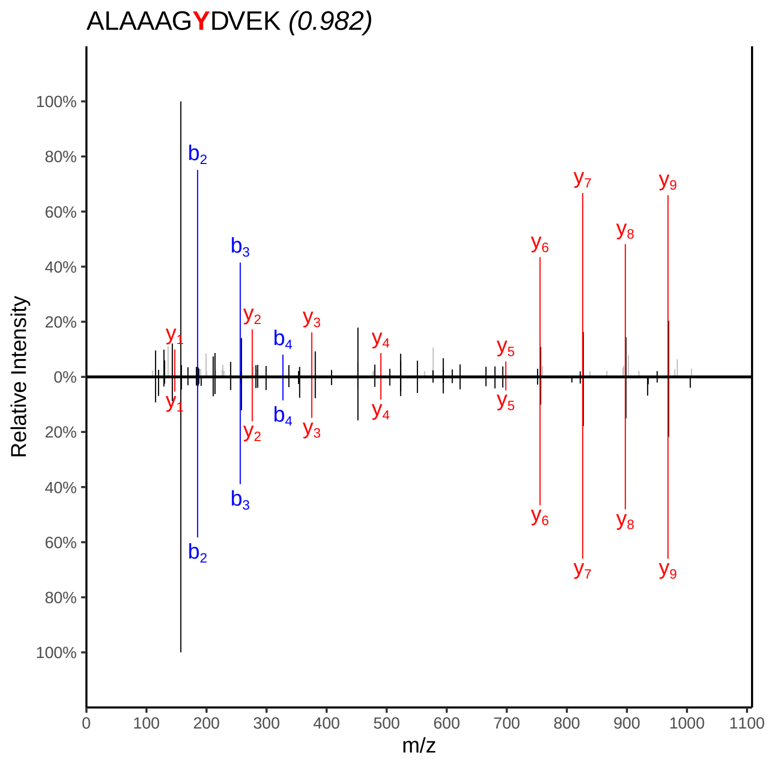
ALAAAGYDVEK (0.982)
Relative Intensity
m/z
100%
80%
60%
40%
20%
0%
20%
40%
60%
80%
100%
0
100
200
300
400
500
600
700
800
900
1000
1100
b2
b3
b4
y1
y2
y3
y4
y5
y6
y7
y8
y9

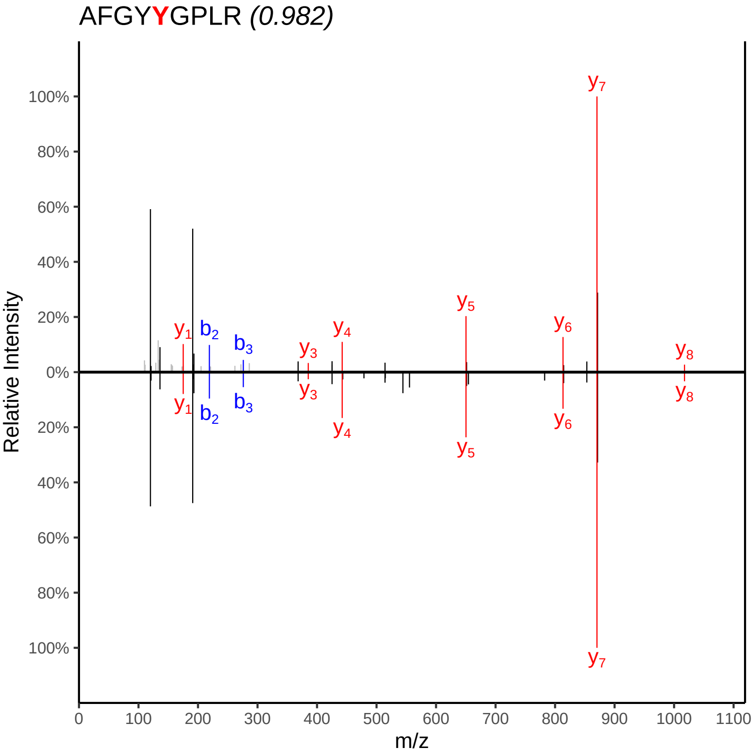

AFGYYGPLR (0.982)
Relative Intensity
m/z
100%
80%
60%
40%
20%
0%
y1
b2
b3
y3
y4
y5
y6
y7
y8
0
100
200
300
400
500
600
700
800
900
1000
1100

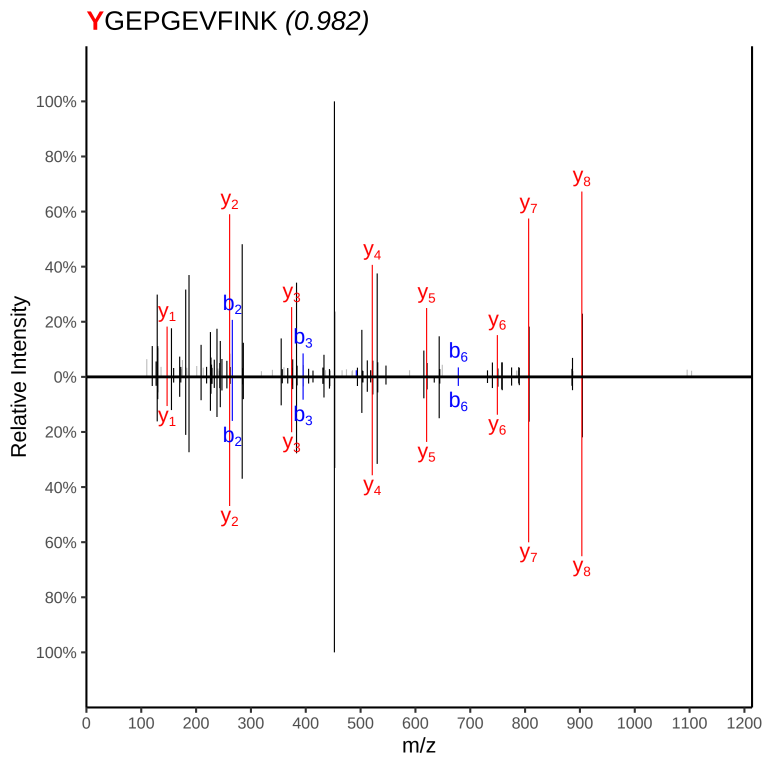

YGEPGEVFINK (0.982)
Relative Intensity
m/z
100%
80%
60%
40%
20%
0%
0
100
200
300
400
500
600
700
800
900
1000
1100
1200
y_1
b_2
y_2
y_3
b_3
y_4
y_5
b_6
y_6
y_7
y_8

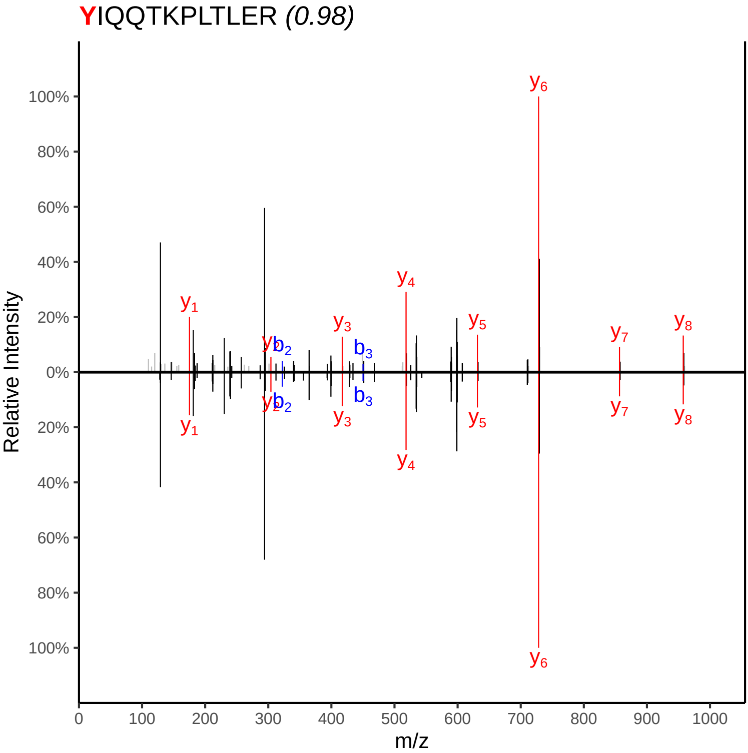

YIQQTKPLTLER (0.98)
Relative Intensity
m/z
100%
80%
60%
40%
20%
0%
0
100
200
300
400
500
600
700
800
900
1000
y1
y2
b2
y3
b3
y4
y5
y6
y7
y8

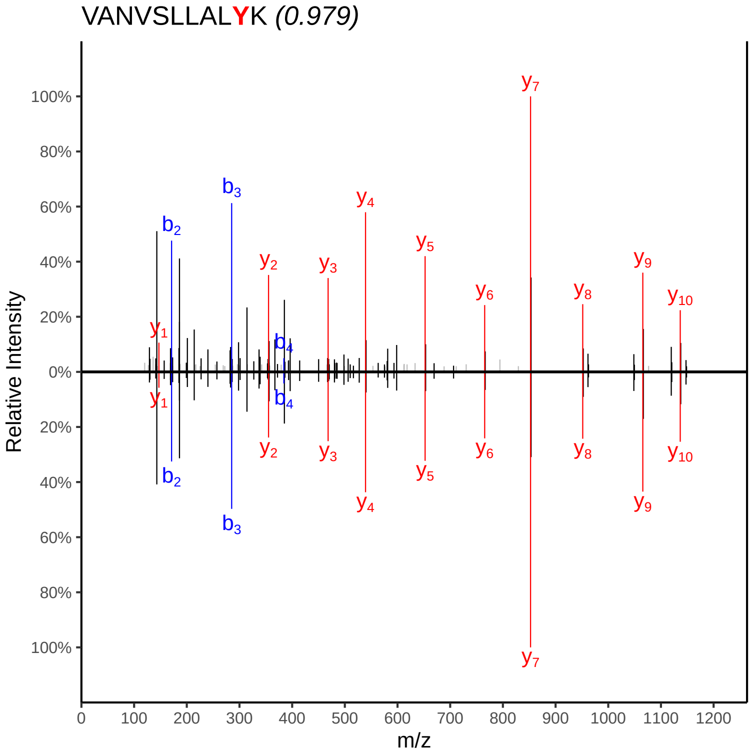
VANVSLLALYK (0.979)
y7
b3
y4
b2
y5
y2
y3
y9
y6
y8
y10
y1
b4
Relative Intensity
100%
80%
60%
40%
20%
0%
m/z
0
100
200
300
400
500
600
700
800
900
1000
1100
1200

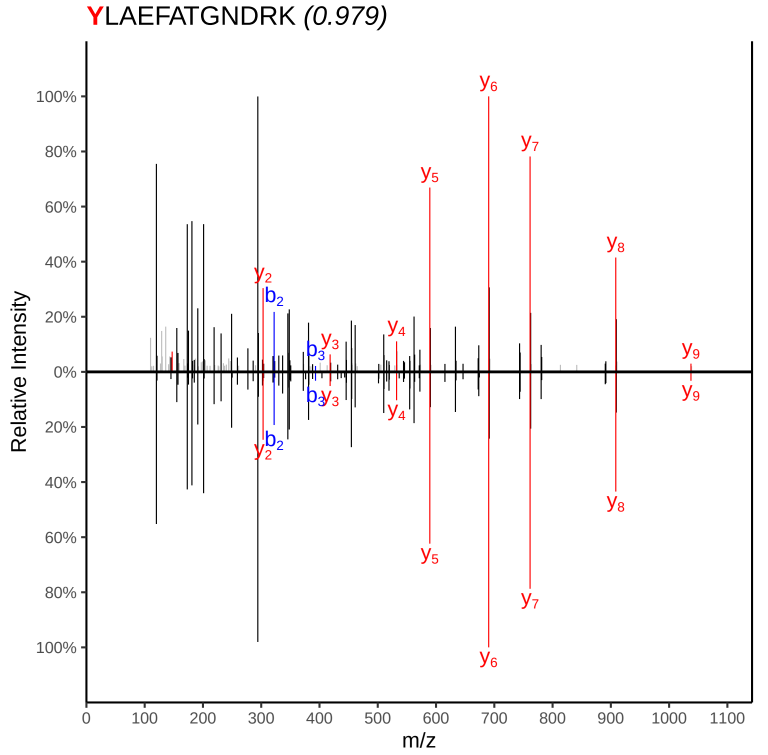
YLAEFATGNDRK (0.979)
Relative Intensity
m/z
0%
20%
40%
60%
80%
100%
0
100
200
300
400
500
600
700
800
900
1000
1100
y2
b2
b3
y3
y4
y5
y6
y7
y8
y9

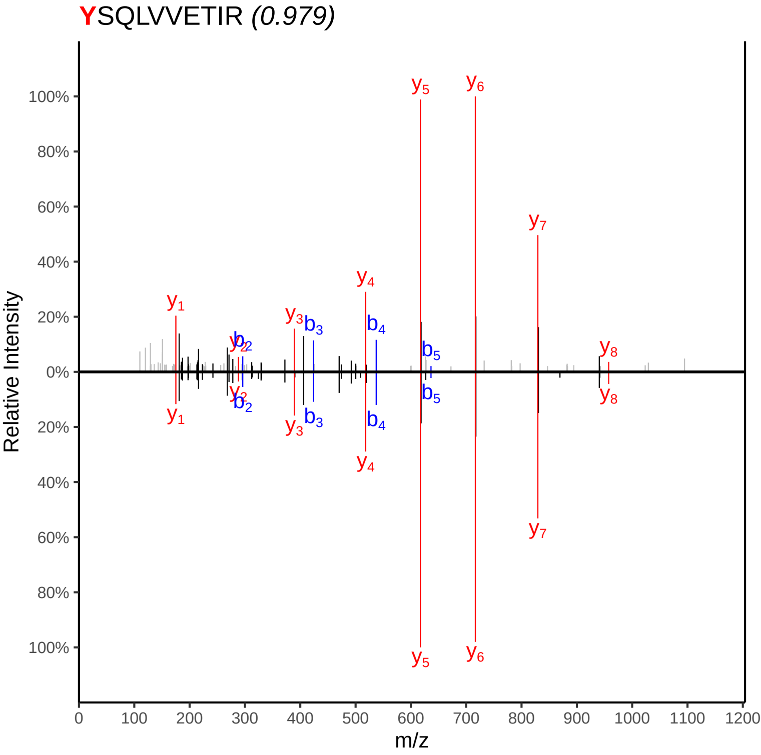

YSQLVVETIR (0.979)
Relative Intensity
m/z
y1
y2
b2
y3
b3
y4
b4
y5
b5
y6
y7
y8

GYSFTTTAER *(0.978)*

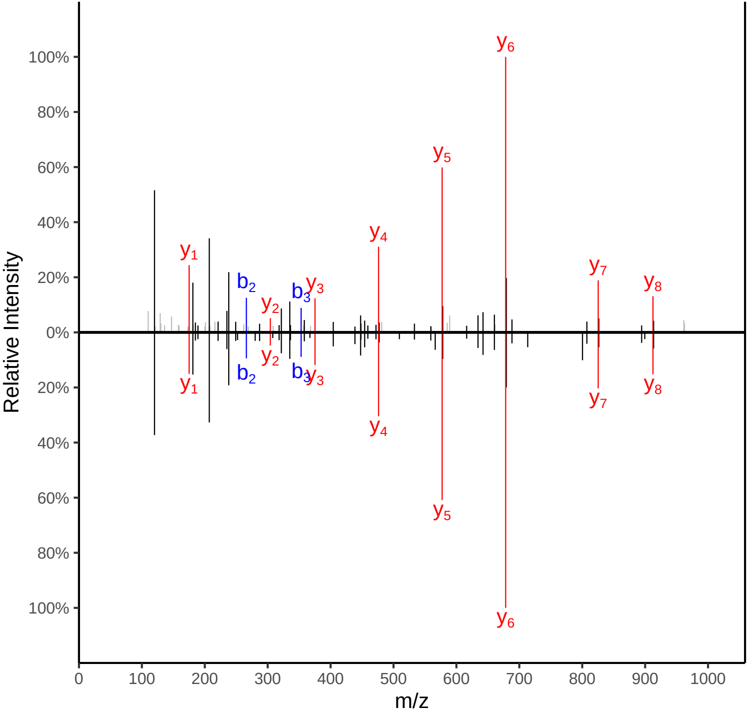

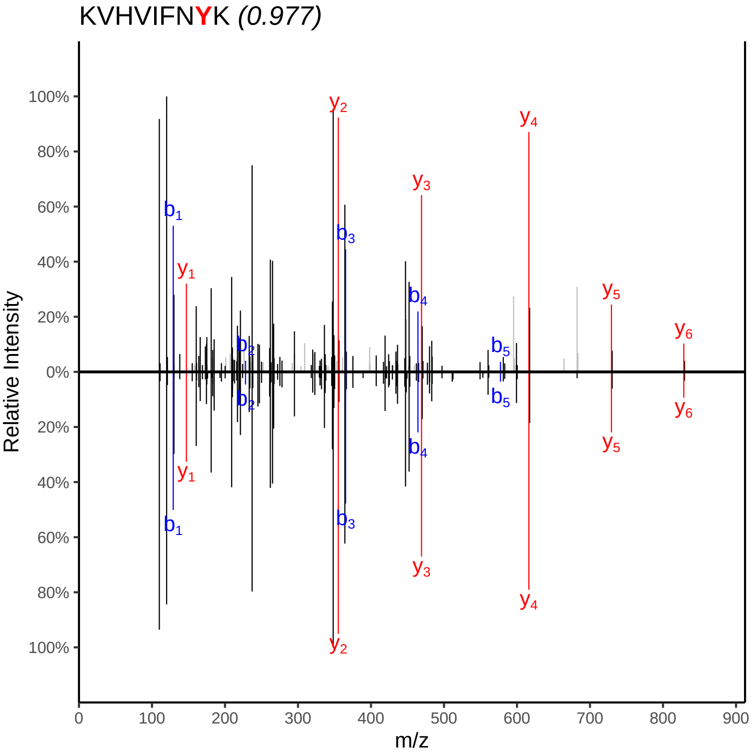
KVHVIFNYK (0.977)
Relative Intensity
m/z
100%
80%
60%
40%
20%
0%
20%
40%
60%
80%
100%
0
100
200
300
400
500
600
700
800
900
b1
y1
b2
y2
b3
y3
b4
y4
b5
y5
y6

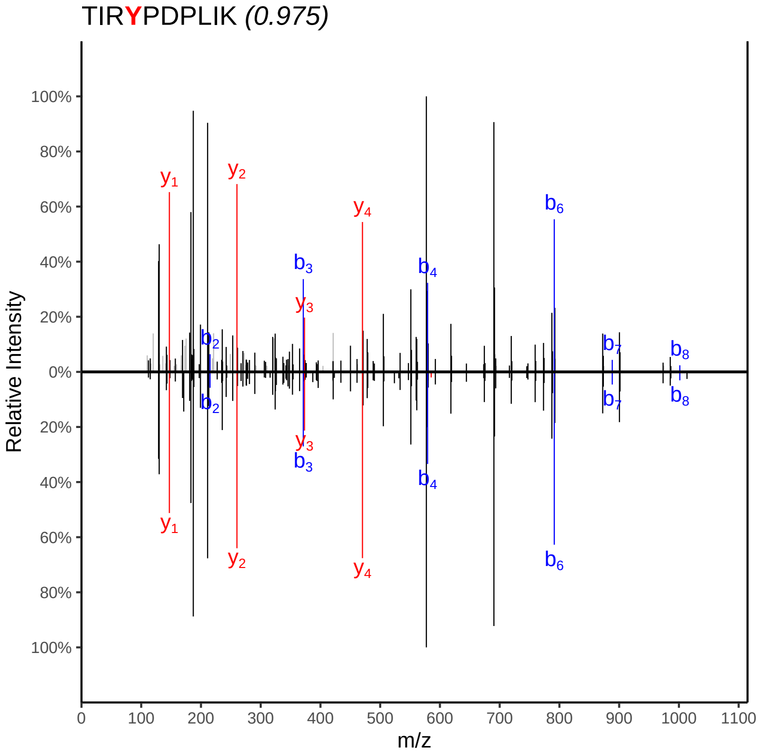

TIRYPDPLIK (0.975)
Relative Intensity
100%
80%
60%
40%
20%
0%
20%
40%
60%
80%
100%
m/z
0
100
200
300
400
500
600
700
800
900
1000
1100
y_1
y_2
y_3
y_4
b_2
b_3
b_4
b_6
b_7
b_8
y_1
y_2
y_3
y_4
b_2
b_3
b_4
b_6
b_7
b_8

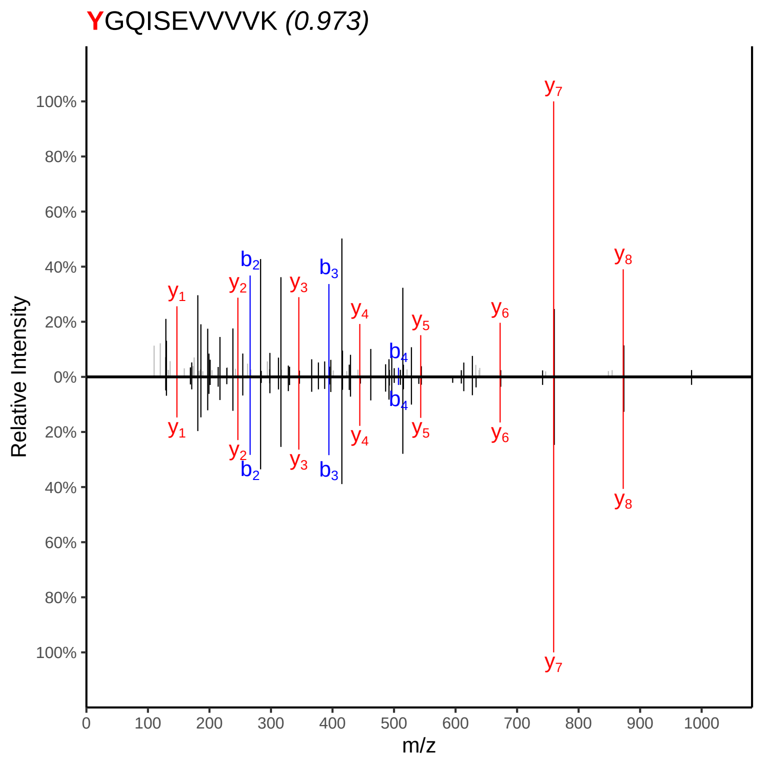

YGQISEVVVVK (0.973)
Relative Intensity
m/z
100%
80%
60%
40%
20%
0%
20%
40%
60%
80%
100%
0
100
200
300
400
500
600
700
800
900
1000
y1
y2
b2
y3
b3
y4
b4
y5
y6
y7
y8

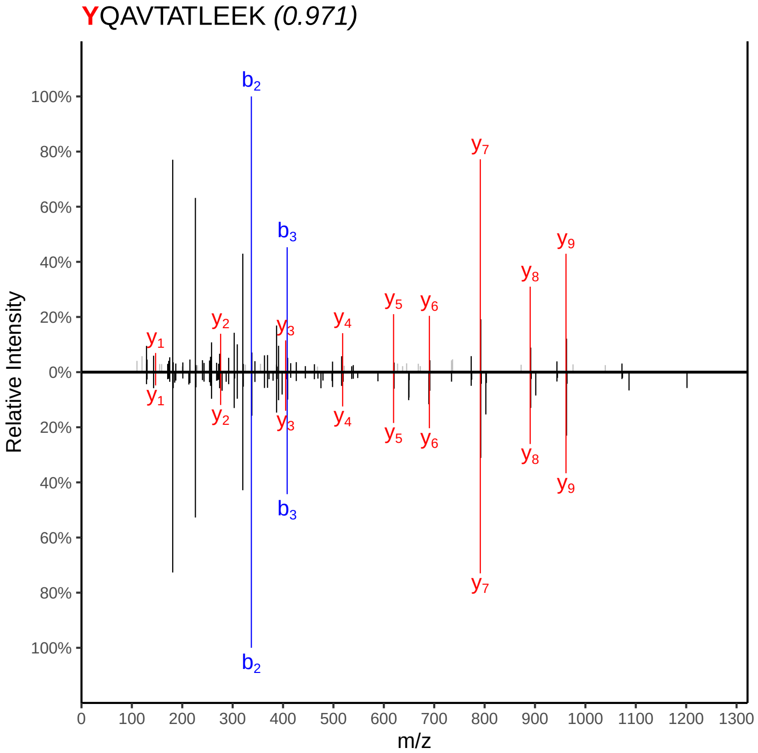
YQAVTATLEEK (0.971)
Relative Intensity
m/z
b2
b3
y1
y2
y3
y4
y5
y6
y7
y8
y9
100%
80%
60%
40%
20%
0%
0
100
200
300
400
500
600
700
800
900
1000
1100
1200
1300

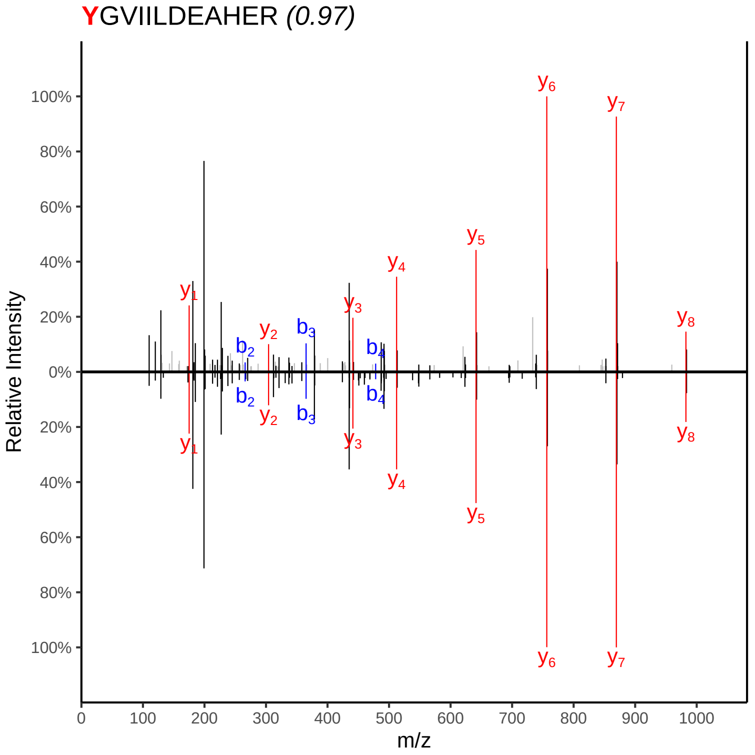

YGVIILDEAHER (0.97)
Relative Intensity
m/z
y1
y2
y3
y4
y5
y6
y7
y8
b2
b3
b4
100%
80%
60%
40%
20%
0%
0
100
200
300
400
500
600
700
800
900
1000

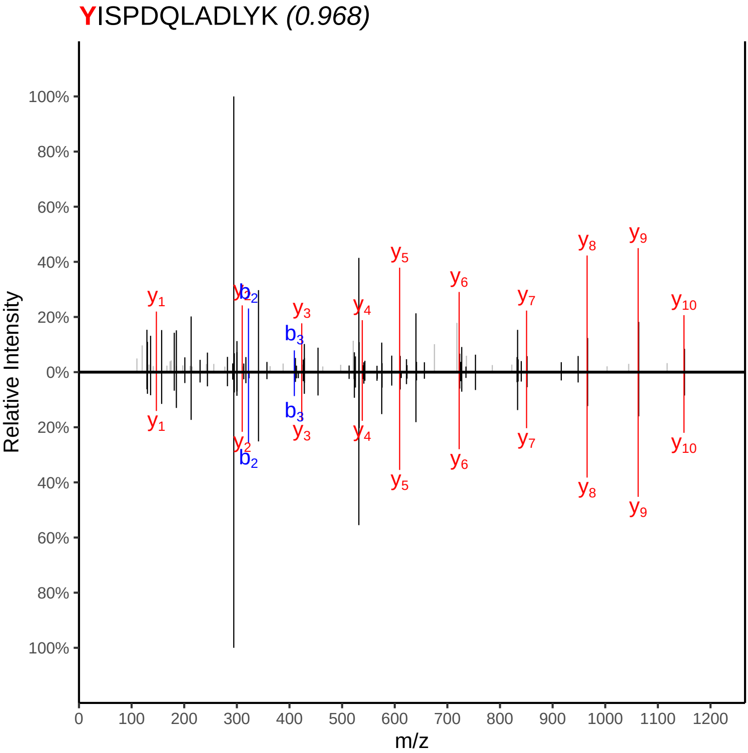
YISPDQLADLYK (0.968)
Relative Intensity
m/z
100%
80%
60%
40%
20%
0%
20%
40%
60%
80%
100%
0
100
200
300
400
500
600
700
800
900
1000
1100
1200
y1
y2
b2
b3
y3
y4
y5
y6
y7
y8
y9
y10

EHALLAYTLGVK *(0.968)*

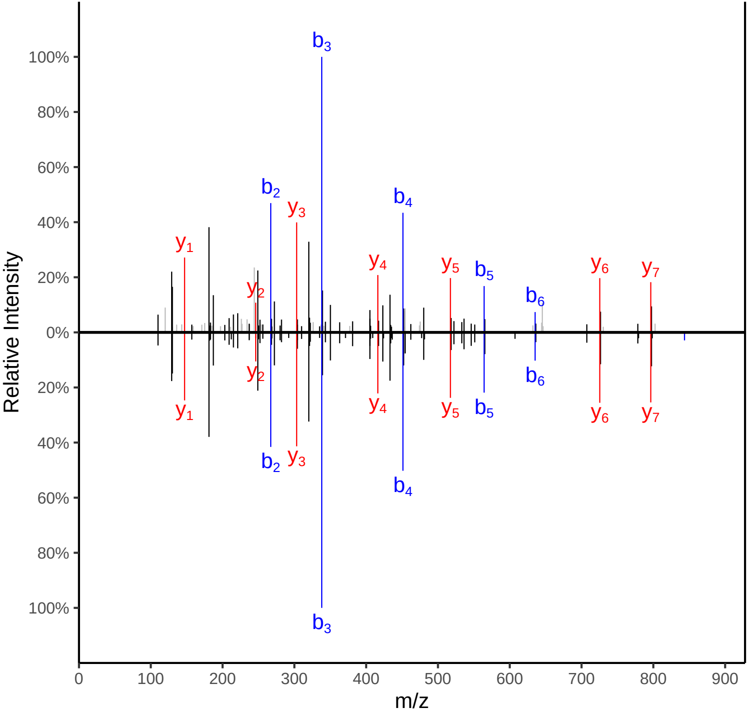

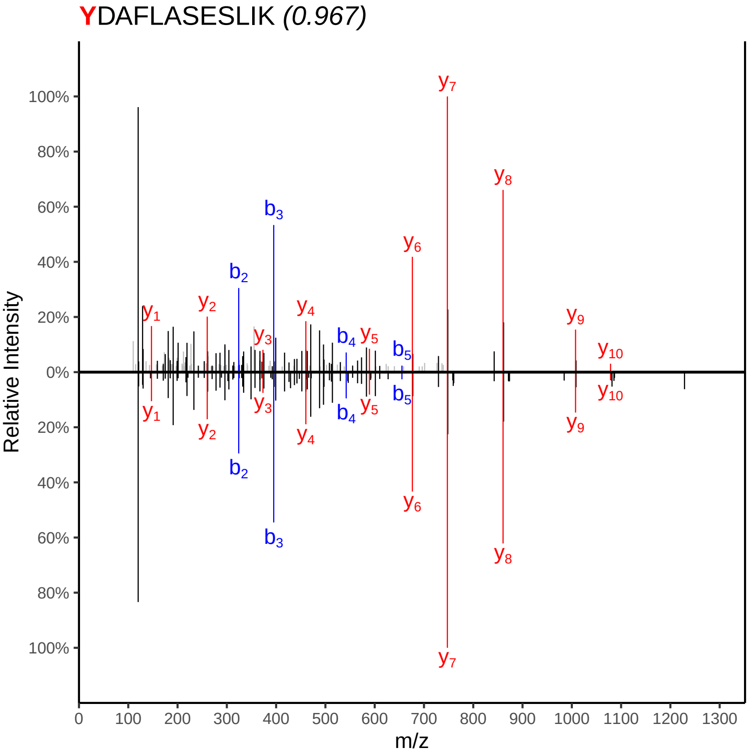

YDAFLASESLIK (0.967)
Relative Intensity
m/z
100%
80%
60%
40%
20%
0%
y1
y2
y3
y4
y5
y6
y7
y8
y9
y10
b2
b3
b4
b5
0
100
200
300
400
500
600
700
800
900
1000
1100
1200
1300

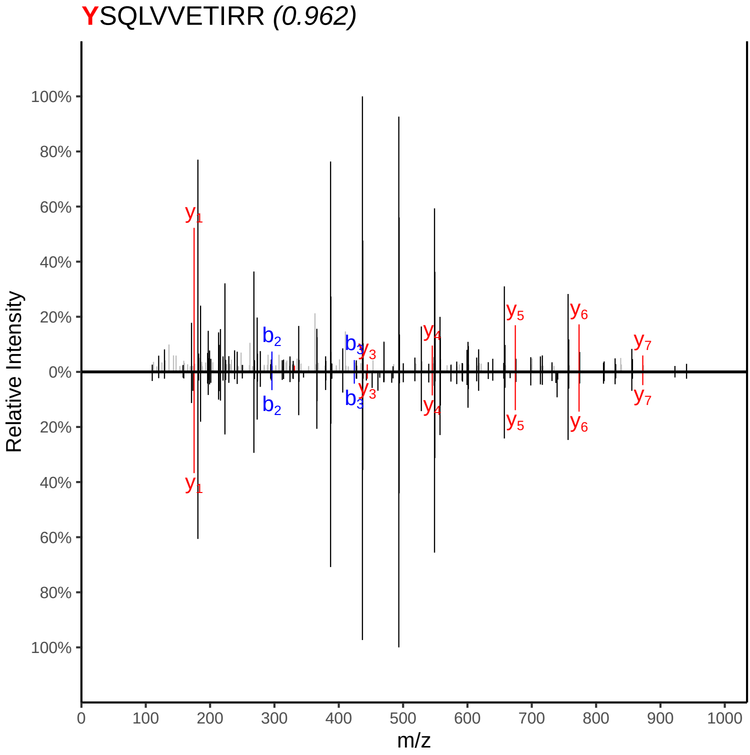
YSQLVVETIRR (0.962)
100%
80%
60%
40%
20%
0%
20%
40%
60%
80%
100%
Relative Intensity
y1
b2
b3
y3
y4
y5
y6
y7
0
100
200
300
400
500
600
700
800
900
1000
m/z

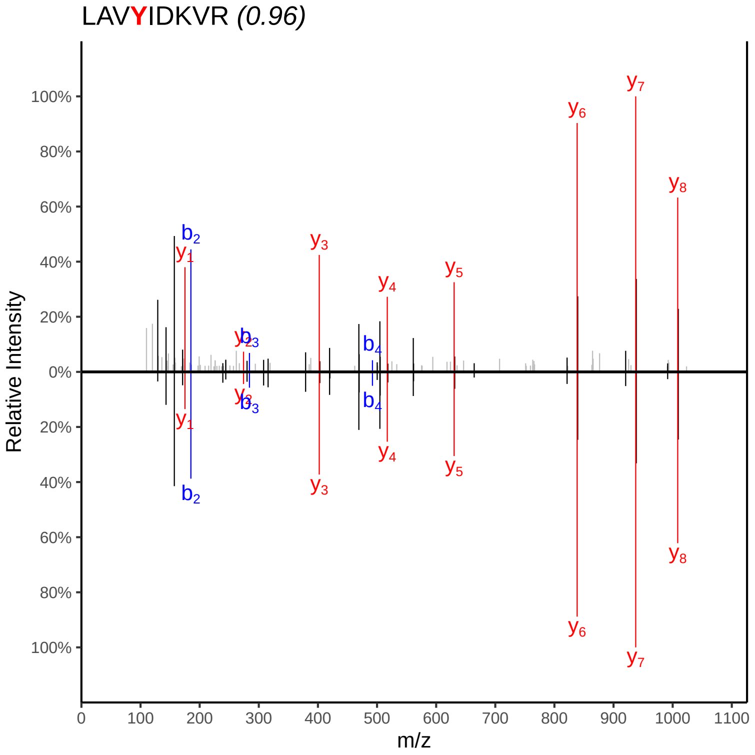

LAVYIDKVR (0.96)
Relative Intensity
m/z
y1
b2
y2
b3
y3
b4
y4
y5
y6
y7
y8

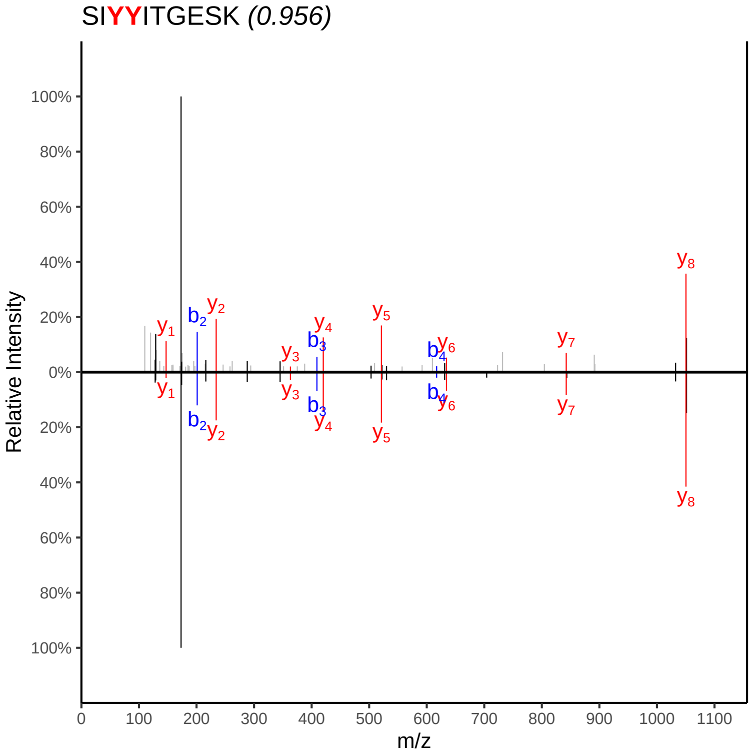

SIYYITGESK (0.956)
Relative Intensity
m/z
y1
b2
y2
y3
b3
y4
y5
b4
y6
y7
y8

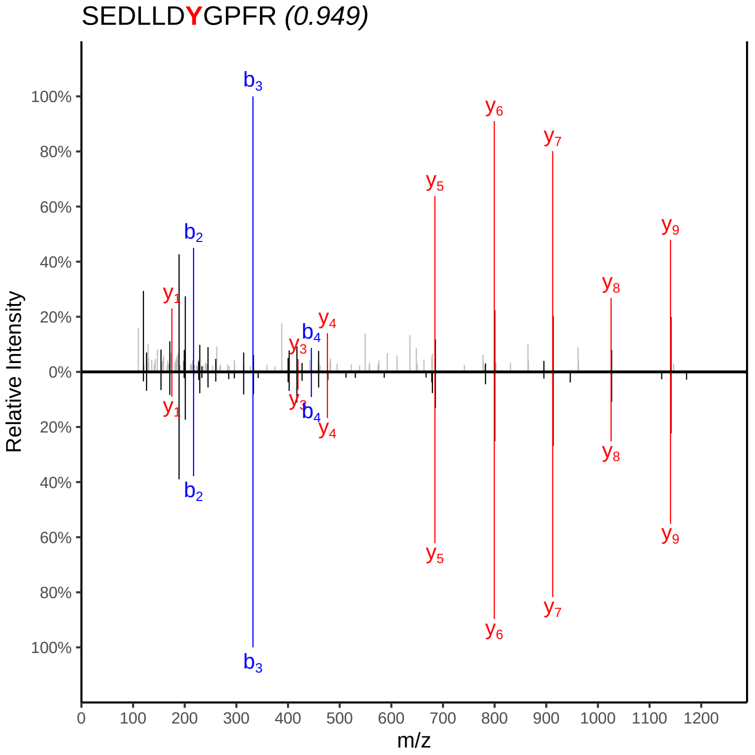

SEDLLDYGPFR (0.949)
Relative Intensity
m/z
b3
b2
b4
y1
y3
y4
y5
y6
y7
y8
y9
100%
80%
60%
40%
20%
0%
0
100
200
300
400
500
600
700
800
900
1000
1100
1200

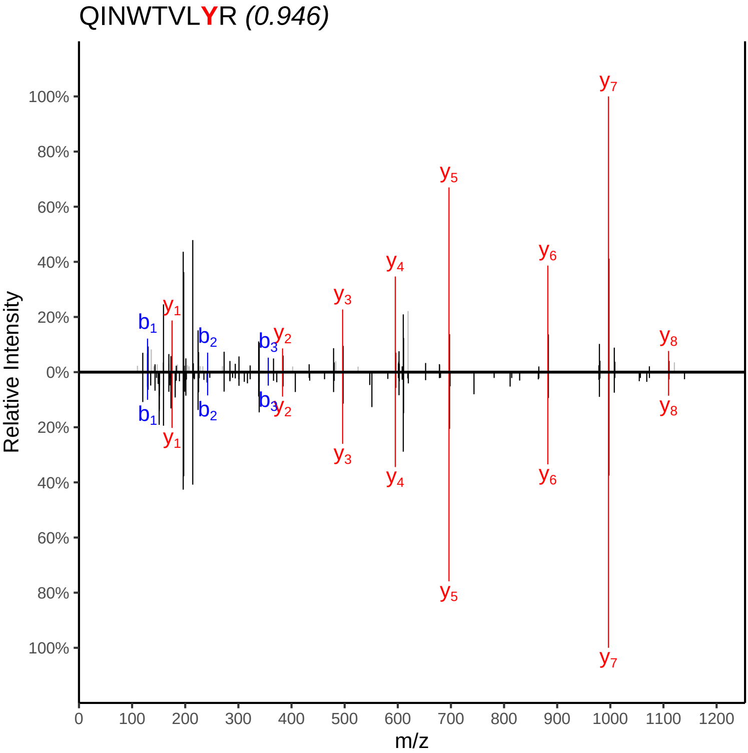
QINWTVLYR (0.946)
Relative Intensity
m/z
100%
80%
60%
40%
20%
0%
20%
40%
60%
80%
100%
0
100
200
300
400
500
600
700
800
900
1000
1100
1200
b1
b2
b3
y1
y2
y3
y4
y5
y6
y7
y8

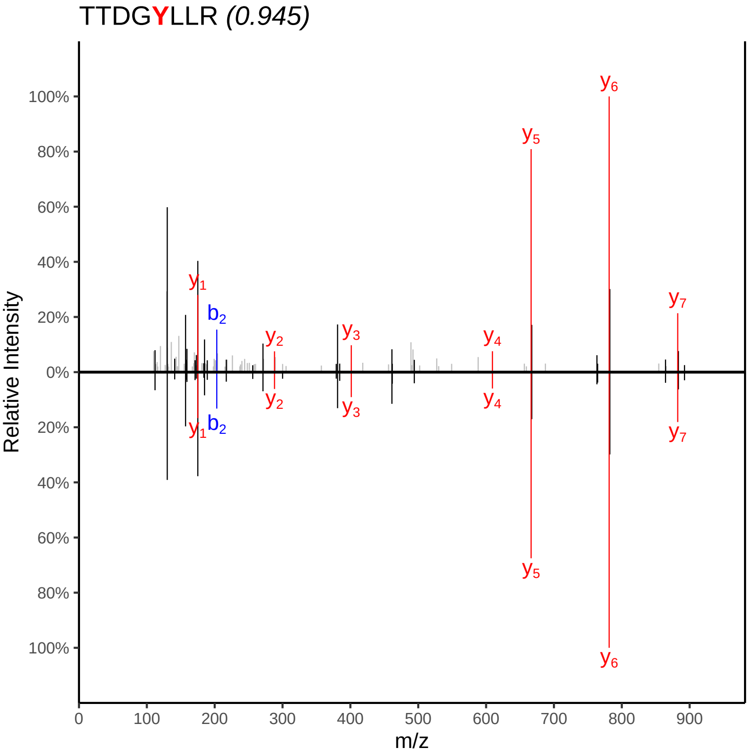
TTDGYLLR (0.945)
Relative Intensity
m/z
y1
b2
y2
y3
y4
y5
y6
y7
100%
80%
60%
40%
20%
0%
0
100
200
300
400
500
600
700
800
900

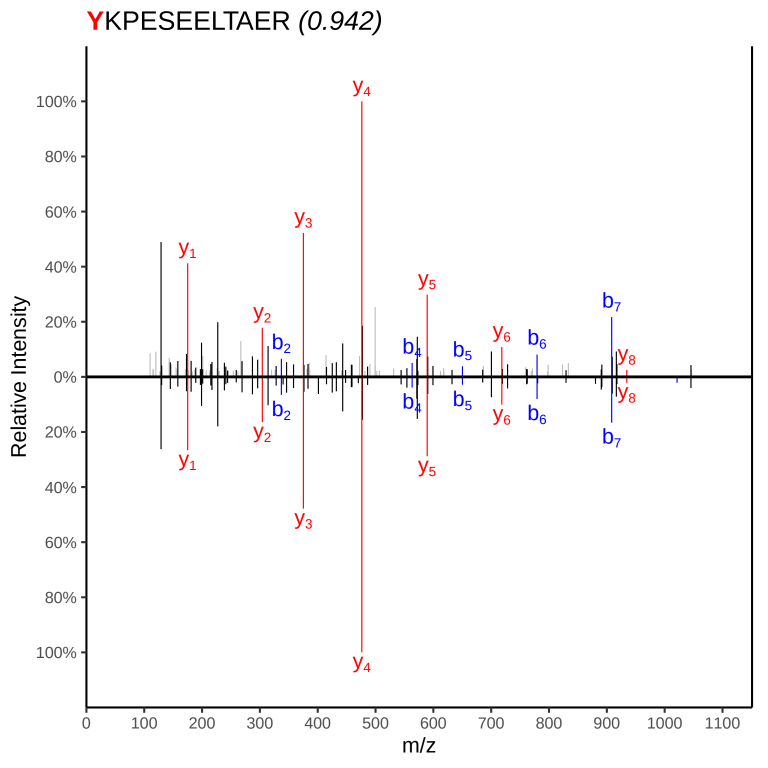

YKPESEELTAER (0.942)
Relative Intensity
m/z
100%
80%
60%
40%
20%
0%
20%
40%
60%
80%
100%
0
100
200
300
400
500
600
700
800
900
1000
1100
y4
y3
y1
y5
y2
b2
b7
b4
b5
y6
b6
y8

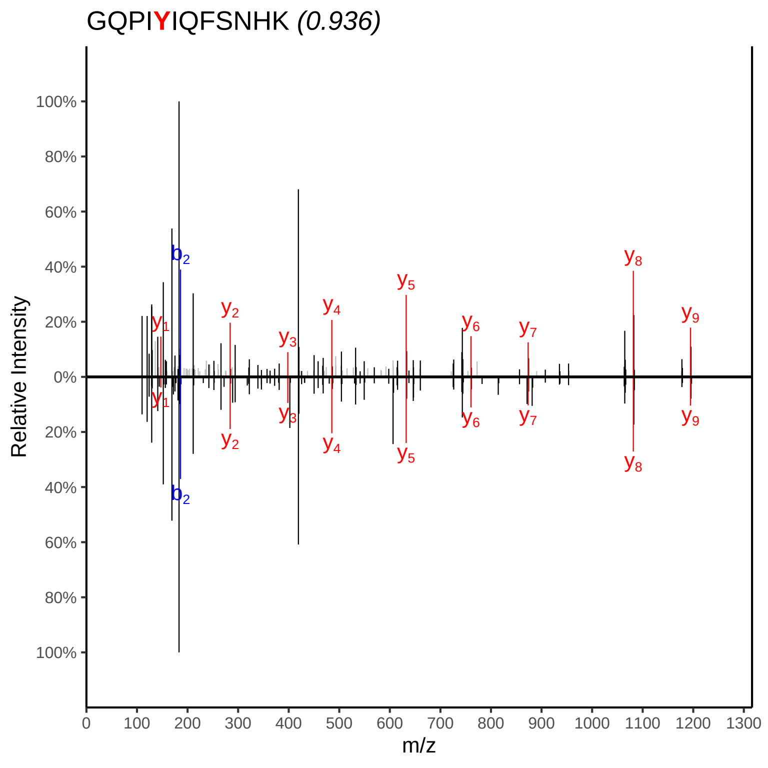
GQPIYIQFSNHK (0.936)
Relative Intensity
m/z
b2
y1
y2
y3
y4
y5
y6
y7
y8
y9

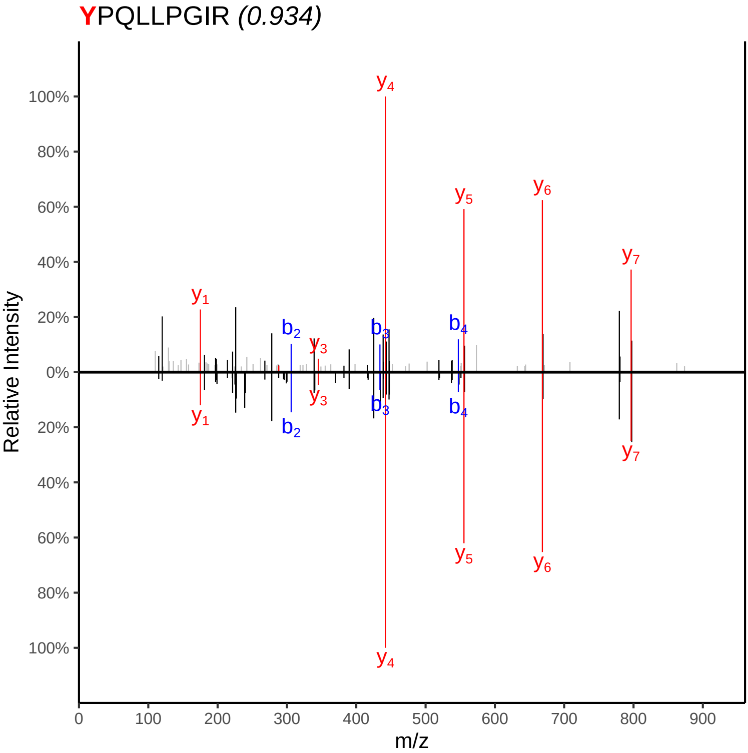
YPQLLPGIR (0.934)
Relative Intensity
m/z
y1
y3
y4
y5
y6
y7
b2
b3
b4

YYVTIIDAPGHR (0.93)

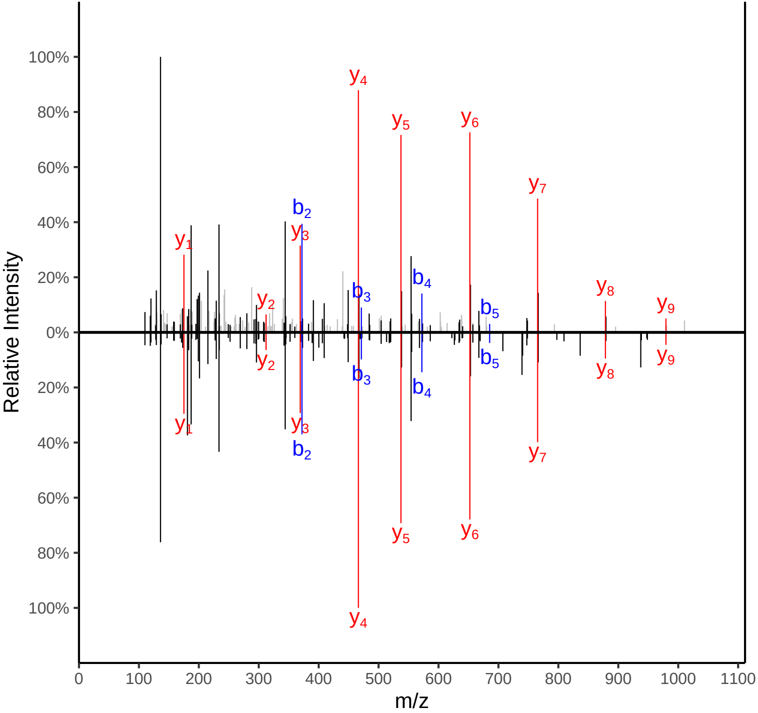

VAVVAG**Y**GDVGK *(0.927)*

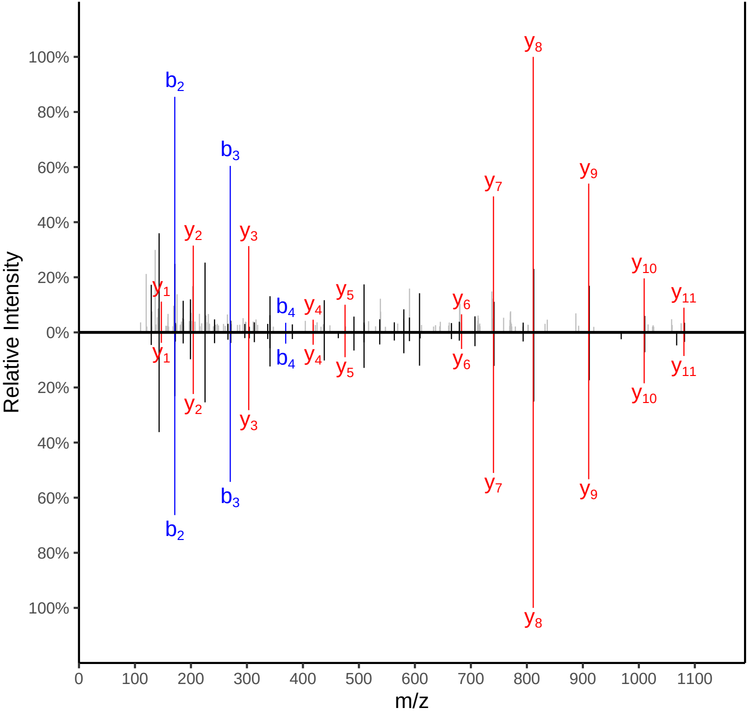

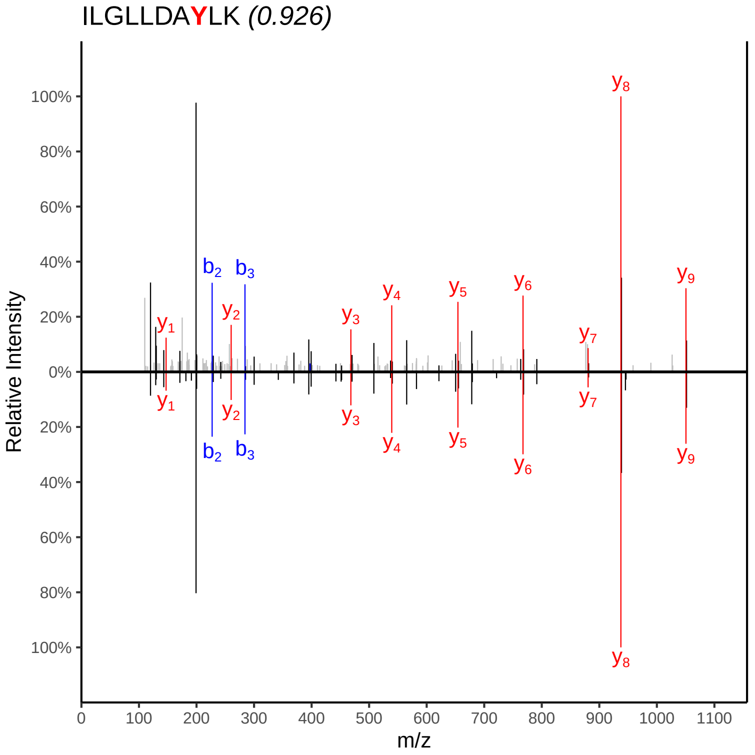

ILGLLDAYLK (0.926)
Relative Intensity
m/z
b2
b3
y1
y2
y3
y4
y5
y6
y7
y8
y9
100%
80%
60%
40%
20%
0%
0
100
200
300
400
500
600
700
800
900
1000
1100

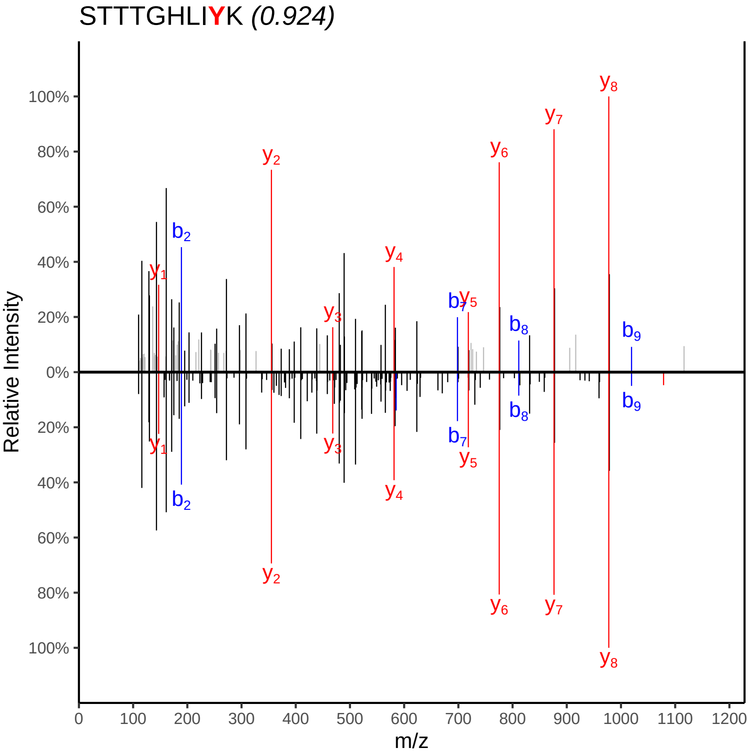

STTTGHLIYK (0.924)
Relative Intensity
m/z
100%
80%
60%
40%
20%
0%
0
100
200
300
400
500
600
700
800
900
1000
1100
1200
y1
b2
y2
y3
y4
b7
y5
y6
b8
y7
y8
b9

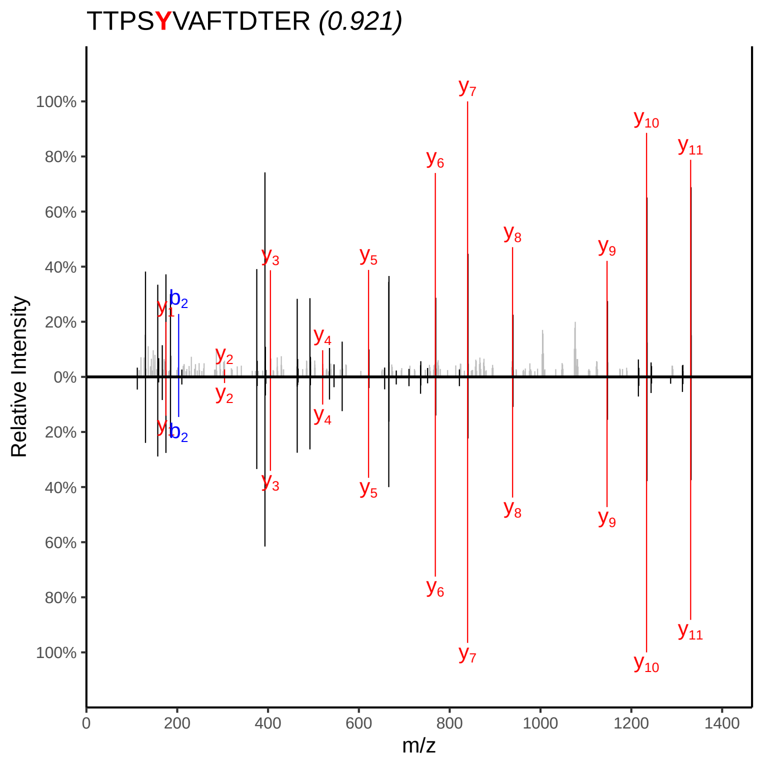
TTPSYVAFTDTER (0.921)
Relative Intensity
m/z
100%
80%
60%
40%
20%
0%
20%
40%
60%
80%
100%
0
200
400
600
800
1000
1200
1400
y1
b2
y2
y3
y4
y5
y6
y7
y8
y9
y10
y11

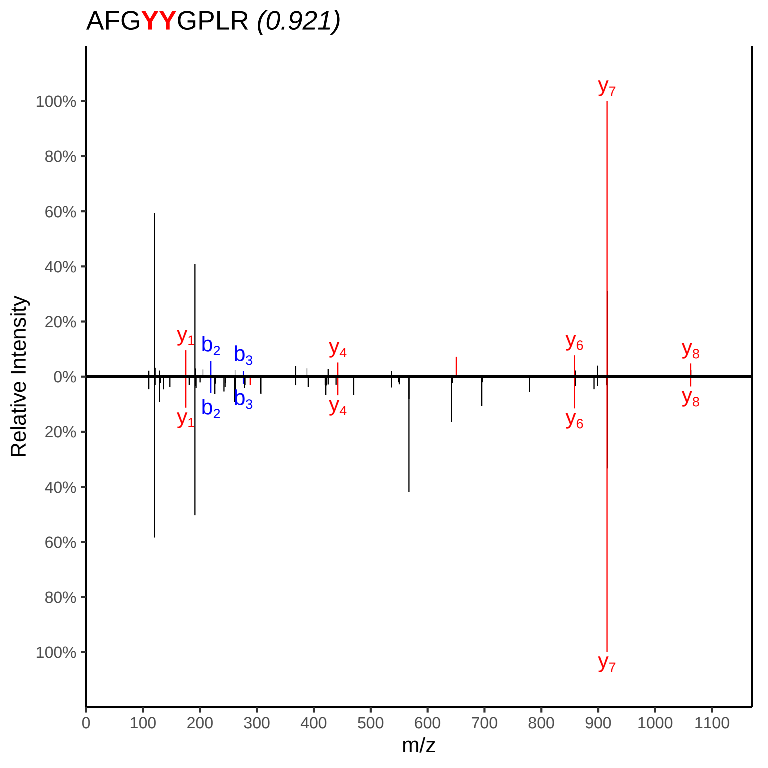

AFGYYGPLR (0.921)
Relative Intensity
m/z
100%
80%
60%
40%
20%
0%
20%
40%
60%
80%
100%
0
100
200
300
400
500
600
700
800
900
1000
1100
y1
b2
b3
y4
y6
y7
y8

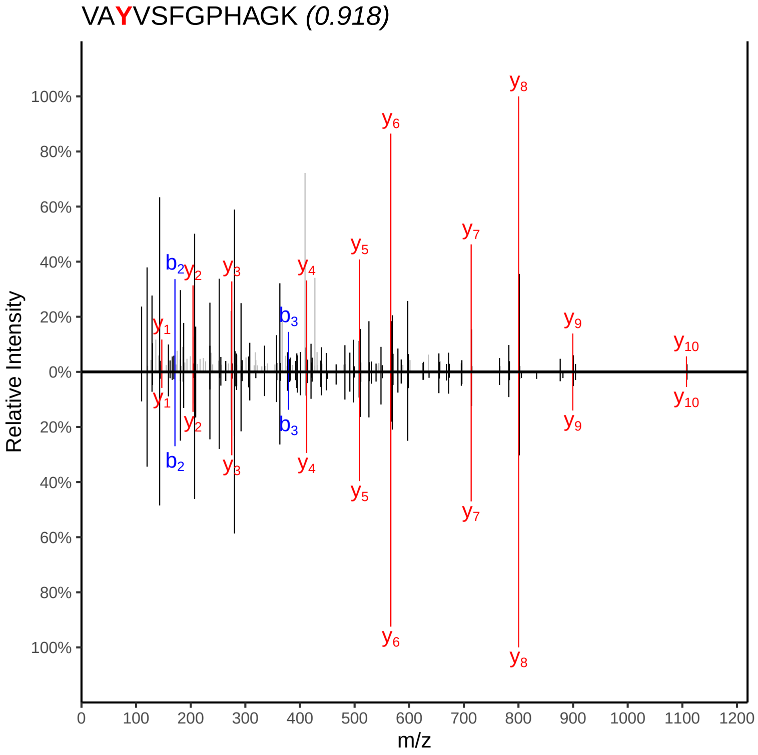

VAYVSFGPHAGK (0.918)
Relative Intensity
m/z
100%
80%
60%
40%
20%
0%
20%
40%
60%
80%
100%
0
100
200
300
400
500
600
700
800
900
1000
1100
1200
y1
b2
y2
y3
b3
y4
y5
y6
y7
y8
y9
y10

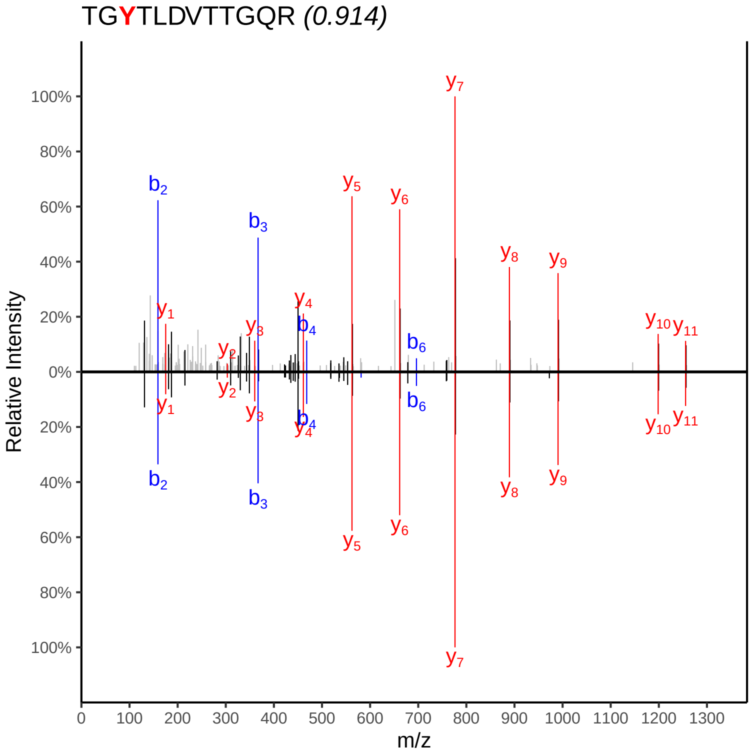
TGYTLDVTTGQR (0.914)
Relative Intensity
m/z
b2
b3
b4
b6
y1
y2
y3
y4
y5
y6
y7
y8
y9
y10
y11

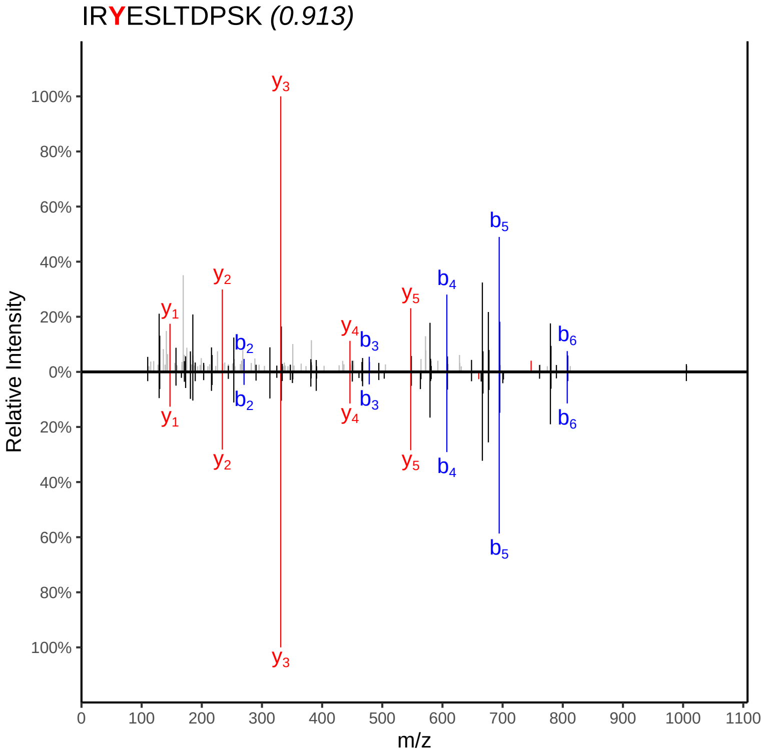
IRYESLTDPSK (0.913)
Relative Intensity
m/z
100%
80%
60%
40%
20%
0%
20%
40%
60%
80%
100%
0
100
200
300
400
500
600
700
800
900
1000
1100
y3
y2
y1
b2
y4
b3
y5
b4
b5
b6

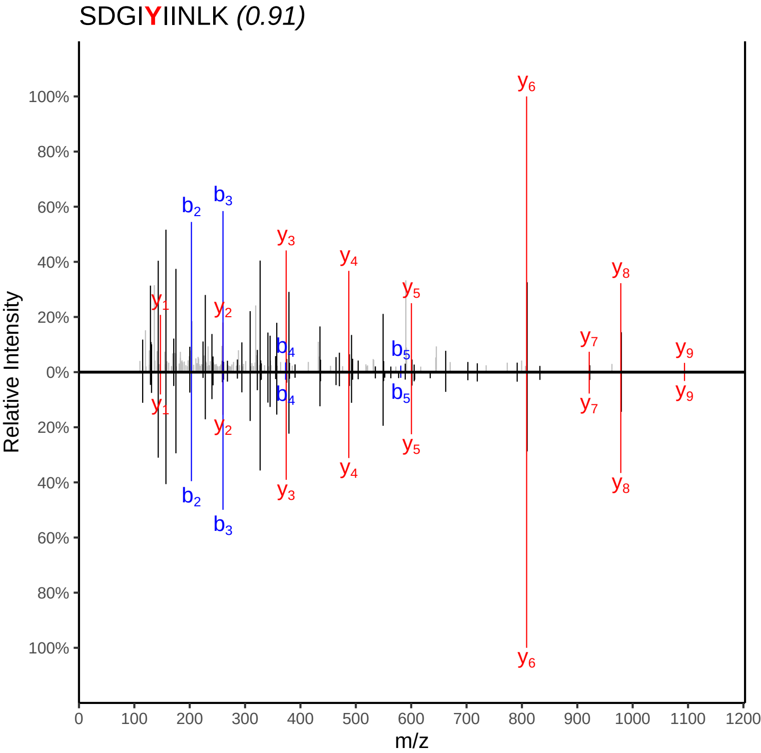
SDGIYIINLK (0.91)
Relative Intensity
m/z
100%
80%
60%
40%
20%
0%
0
100
200
300
400
500
600
700
800
900
1000
1100
1200
b2
b3
b4
b5
y1
y2
y3
y4
y5
y6
y7
y8
y9

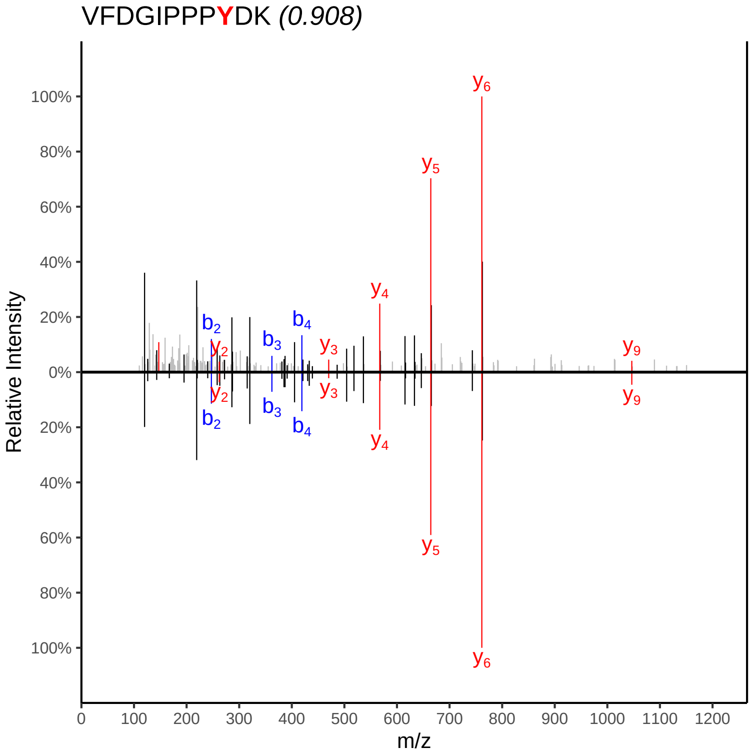

VFDGIPPPYDK (0.908)
Relative Intensity
m/z
100%
80%
60%
40%
20%
0%
20%
40%
60%
80%
100%
0
100
200
300
400
500
600
700
800
900
1000
1100
1200
y6
y5
y4
y3
y2
y9
b2
b3
b4

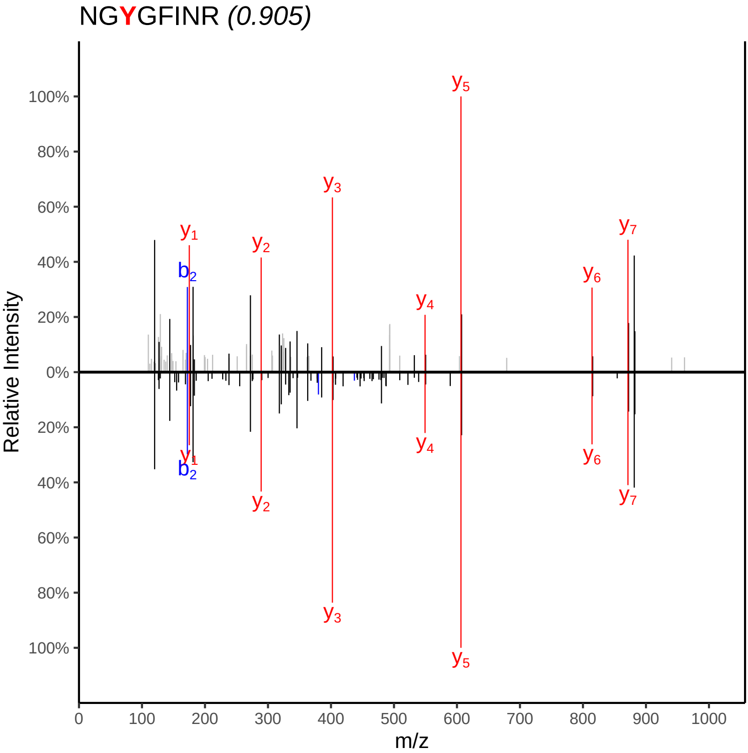

NGYGFINR (0.905)
Relative Intensity
m/z
y1
y2
y3
y4
y5
y6
y7
b2
100%
80%
60%
40%
20%
0%
0
100
200
300
400
500
600
700
800
900
1000

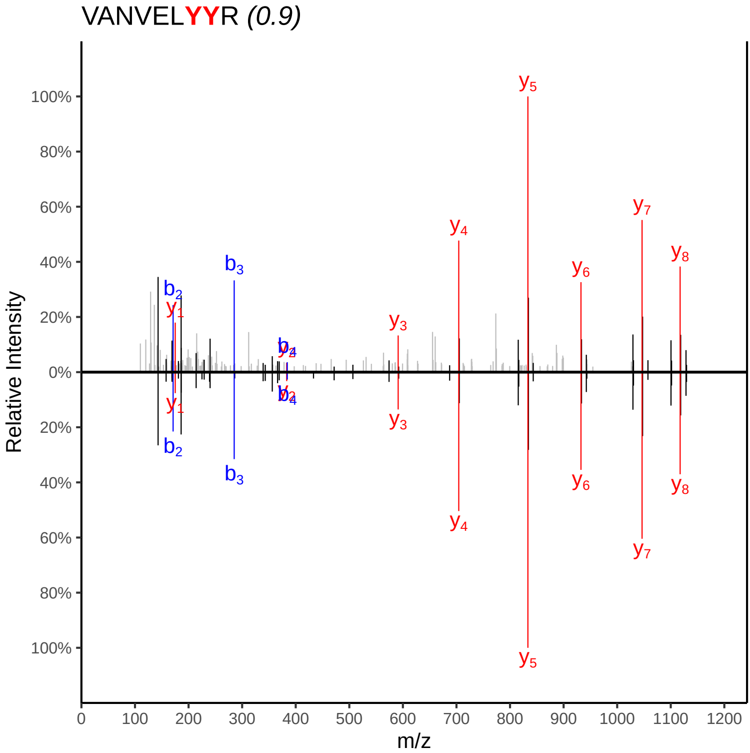

VANVELYYR (0.9)
Relative Intensity
m/z
100%
80%
60%
40%
20%
0%
0
100
200
300
400
500
600
700
800
900
1000
1100
1200
y5
y7
y4
y8
y6
b3
b2
y1
y3
b4
y2

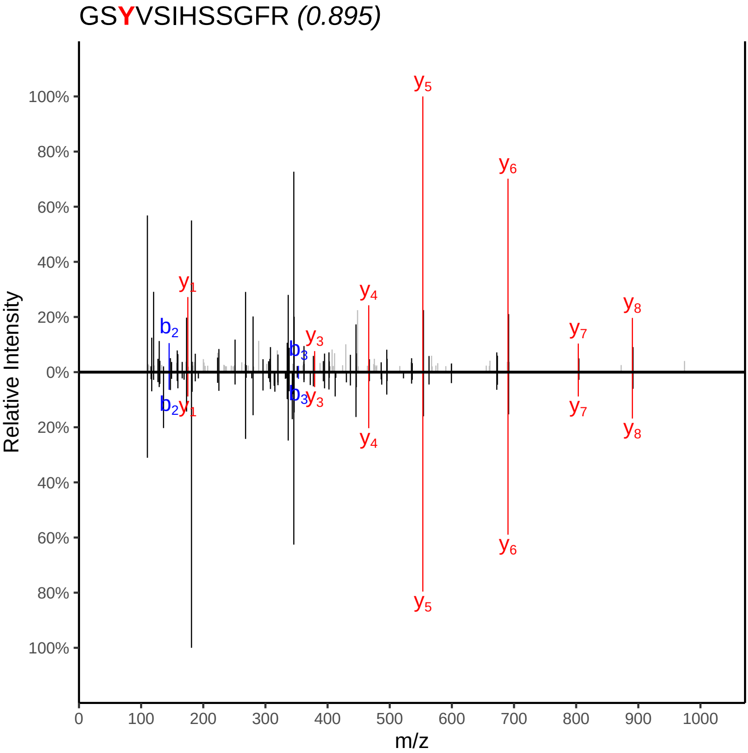

GSYVSIHSSGFR (0.895)
Relative Intensity
m/z
100%
80%
60%
40%
20%
0%
20%
40%
60%
80%
100%
0
100
200
300
400
500
600
700
800
900
1000
y5
y6
y1
y4
y8
y7
y3
b2
b3

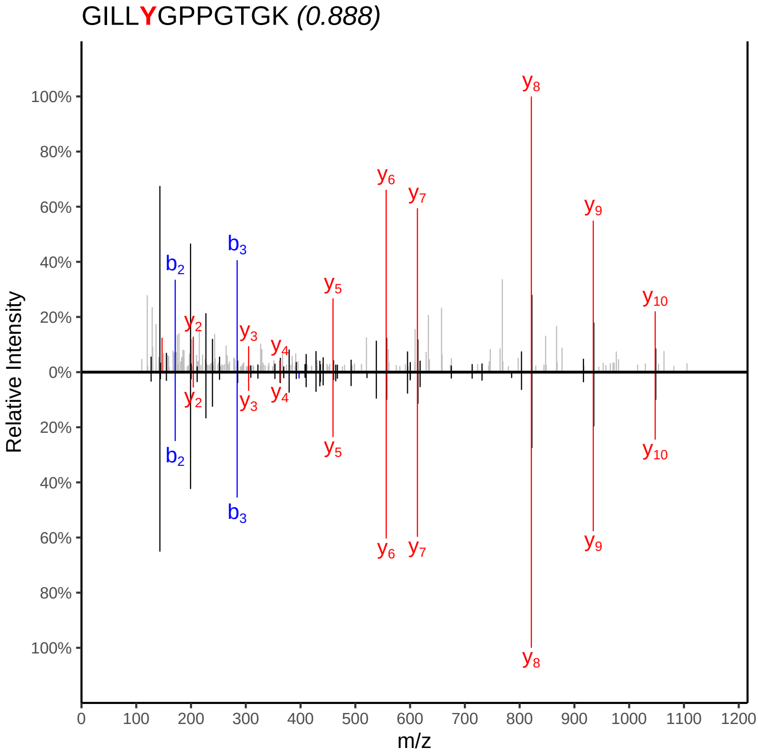

GILLYGPPGTGK (0.888)
Relative Intensity
m/z
y8
y6
y7
y9
b3
b2
y5
y10
y2
y3
y4
100%
80%
60%
40%
20%
0%
0
100
200
300
400
500
600
700
800
900
1000
1100
1200

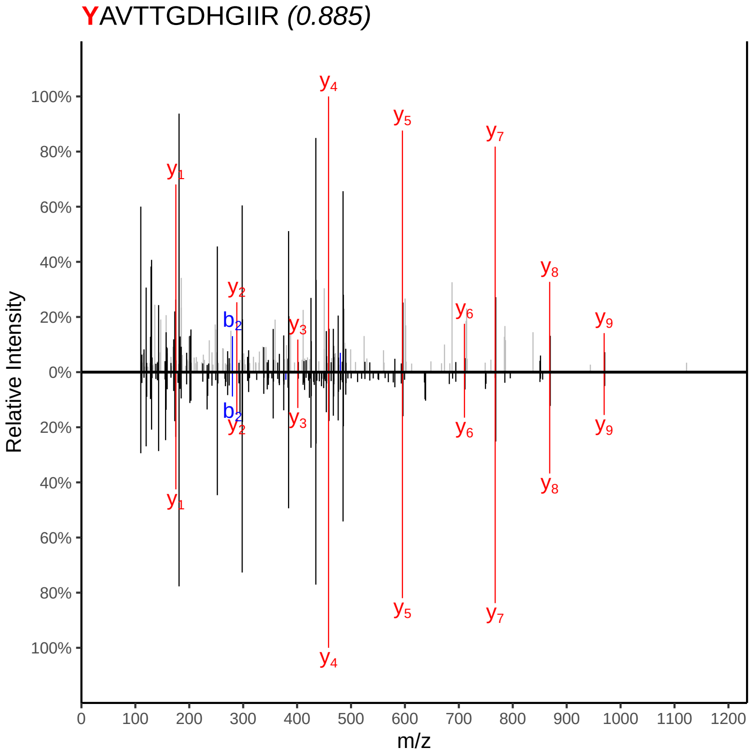
YAVTTGDHGIIR (0.885)
Relative Intensity
m/z
100%
80%
60%
40%
20%
0%
20%
40%
60%
80%
100%
0
100
200
300
400
500
600
700
800
900
1000
1100
1200
y1
y2
b2
y3
y4
y5
y6
y7
y8
y9

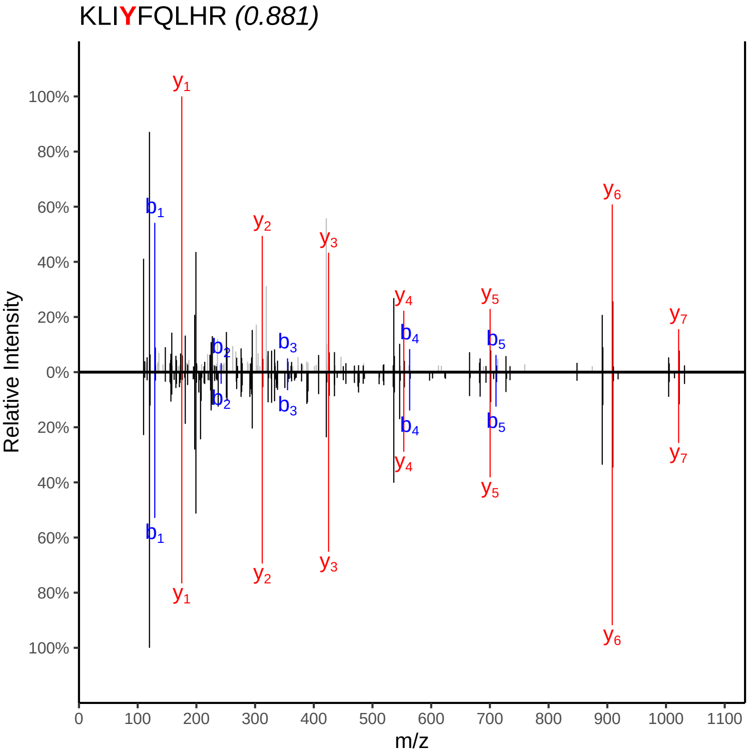

KLIYFQLHR (0.881)
Relative Intensity
m/z
100%
80%
60%
40%
20%
0%
0
100
200
300
400
500
600
700
800
900
1000
1100
y1
y2
y3
y4
y5
y6
y7
b1
b2
b3
b4
b5

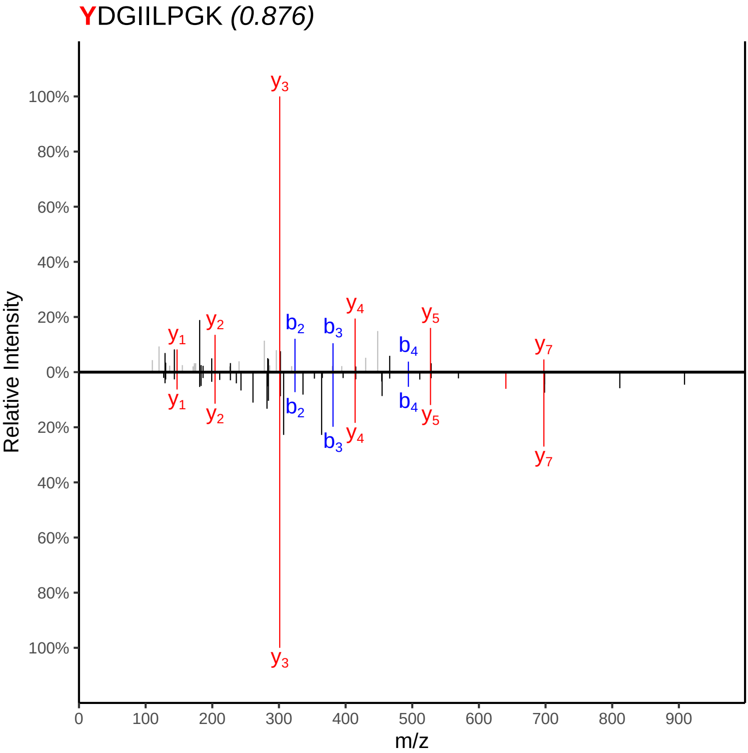

YDGIILPGK (0.876)
Relative Intensity
m/z
100%
80%
60%
40%
20%
0%
20%
40%
60%
80%
100%
0
100
200
300
400
500
600
700
800
900
y1
y2
y3
y4
y5
y7
b2
b3
b4

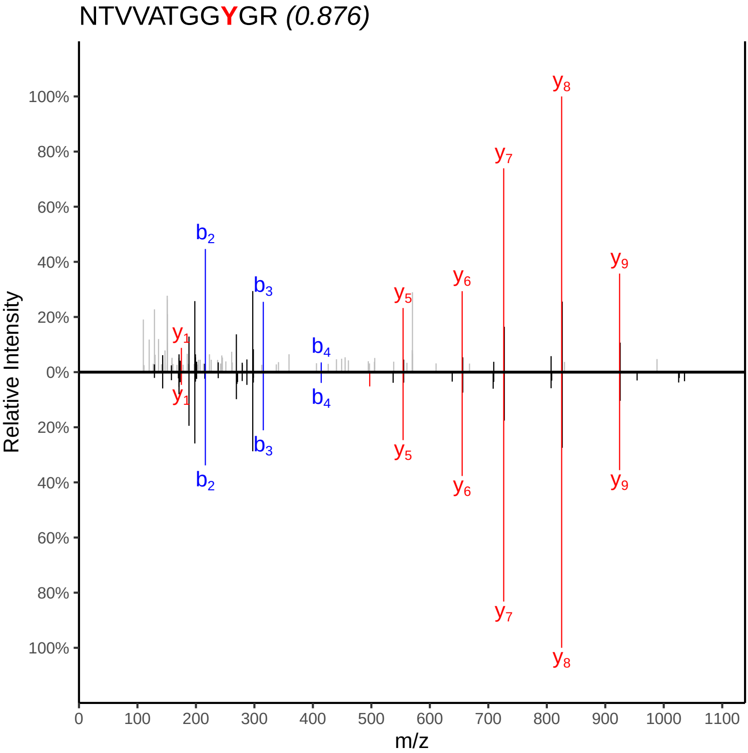
NTVVATGGYGR (0.876)
Relative Intensity
m/z
y1
b2
b3
b4
y5
y6
y7
y8
y9
100%
80%
60%
40%
20%
0%
0
100
200
300
400
500
600
700
800
900
1000
1100

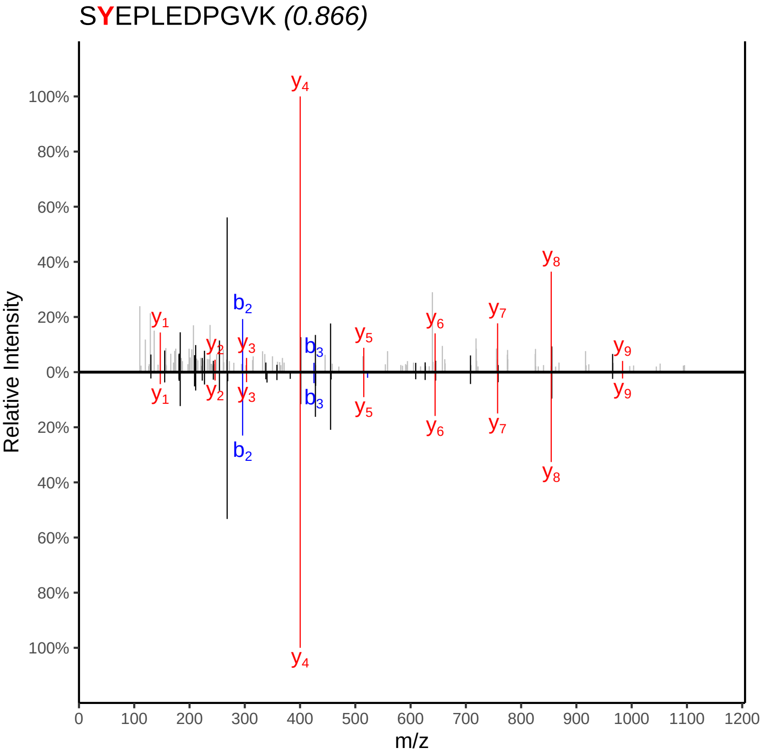

SYEPLEDPGVK (0.866)
Relative Intensity
m/z
100%
80%
60%
40%
20%
0%
0
100
200
300
400
500
600
700
800
900
1000
1100
1200
y_1
y_2
y_3
y_4
y_5
y_6
y_7
y_8
y_9
b_2
b_3

SPYQEFTDHLVK *(0.862)*

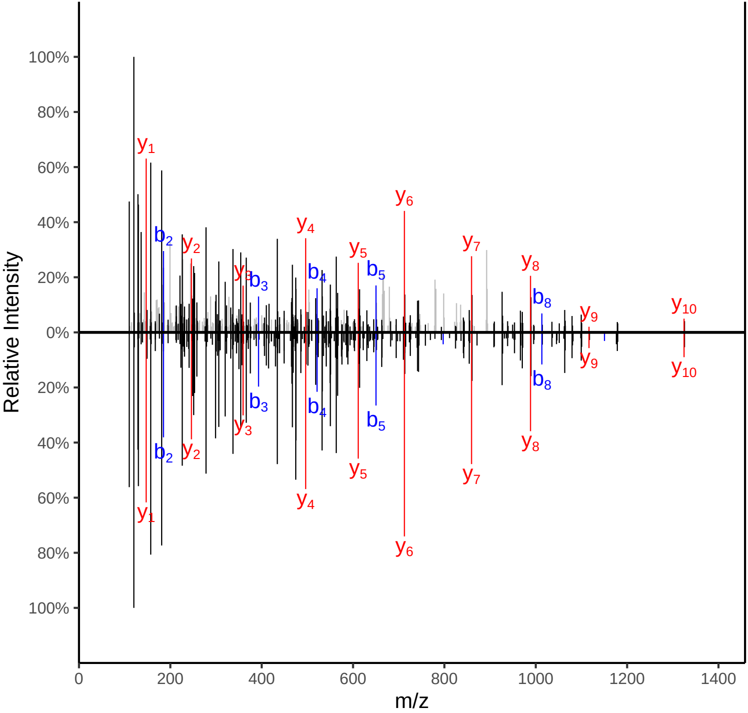

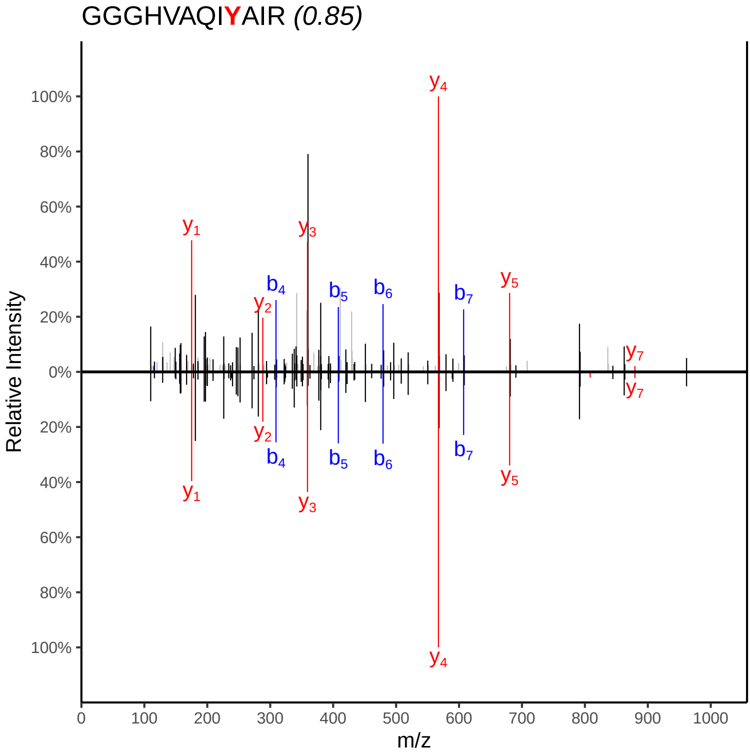
GGGHVAQIYAIR (0.85)
Relative Intensity
m/z
100%
80%
60%
40%
20%
0%
20%
40%
60%
80%
100%
0
100
200
300
400
500
600
700
800
900
1000
y1
y2
y3
y4
y5
y7
b4
b5
b6
b7

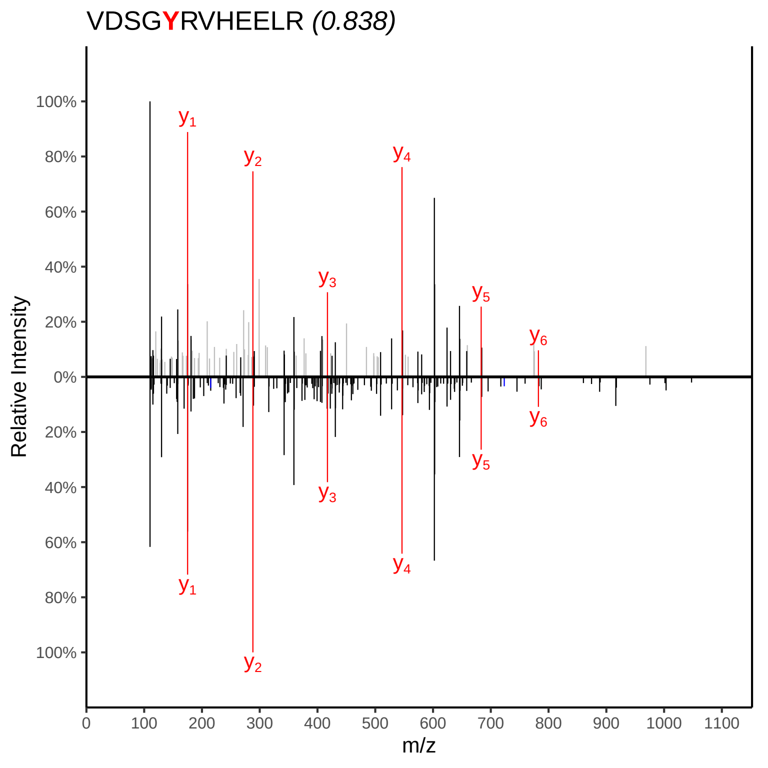

VDSGYRVHEELR (0.838)
Relative Intensity
m/z
100%
80%
60%
40%
20%
0%
20%
40%
60%
80%
100%
0
100
200
300
400
500
600
700
800
900
1000
1100
y_1
y_2
y_3
y_4
y_5
y_6

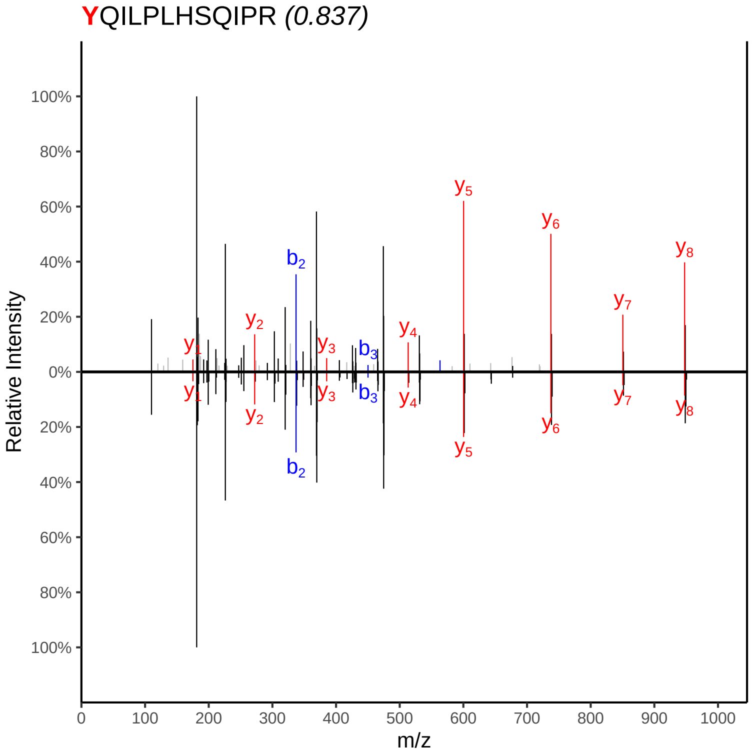

YQILPLHSQIPR (0.837)
Relative Intensity
m/z
100%
80%
60%
40%
20%
0%
20%
40%
60%
80%
100%
0
100
200
300
400
500
600
700
800
900
1000
y1
y2
y3
y4
y5
y6
y7
y8
b2
b3

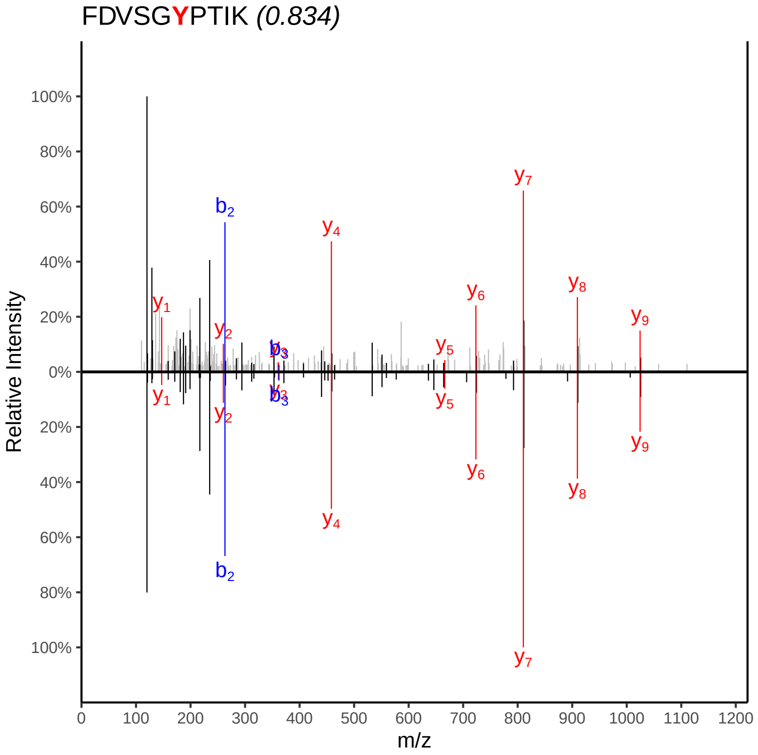

FDVSGYPTIK (0.834)
Relative Intensity
m/z
b2
b3
y1
y2
y3
y4
y5
y6
y7
y8
y9
100%
80%
60%
40%
20%
0%
0
100
200
300
400
500
600
700
800
900
1000
1100
1200

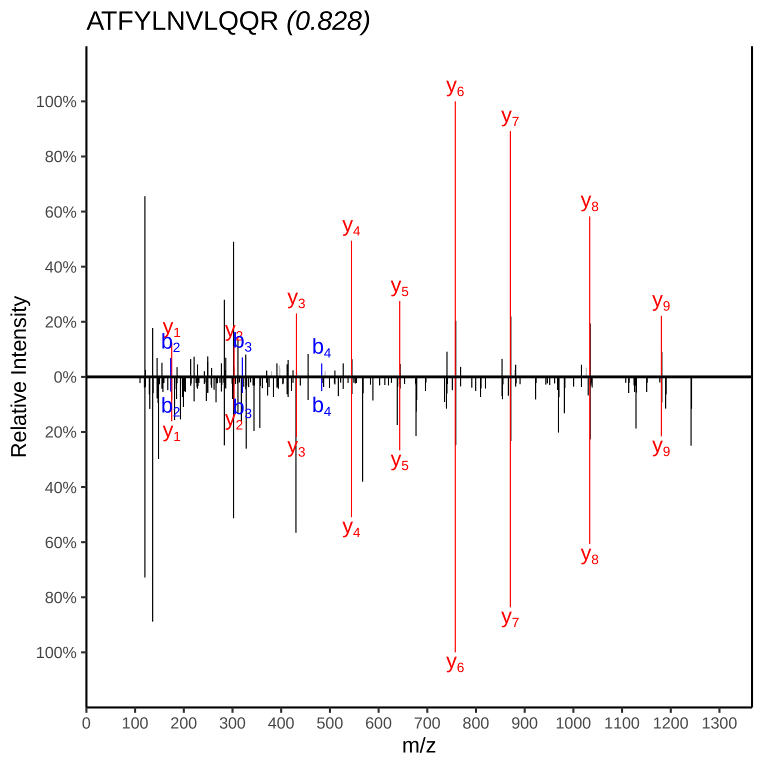

ATFYLNVLQQR (0.828)
Relative Intensity
m/z
100%
80%
60%
40%
20%
0%
0
100
200
300
400
500
600
700
800
900
1000
1100
1200
1300
y1
b2
y2
b3
y3
b4
y4
y5
y6
y7
y8
y9

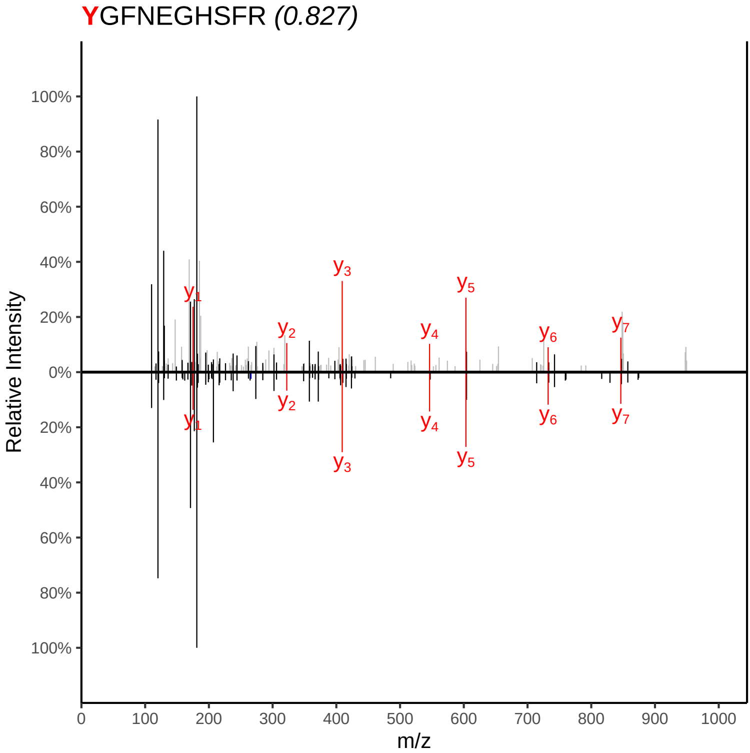

YGFNEGHSFR (0.827)
Relative Intensity
100%
80%
60%
40%
20%
0%
20%
40%
60%
80%
100%
y1
y2
y3
y4
y5
y6
y7
y1
y2
y3
y4
y5
y6
y7
0
100
200
300
400
500
600
700
800
900
1000
m/z

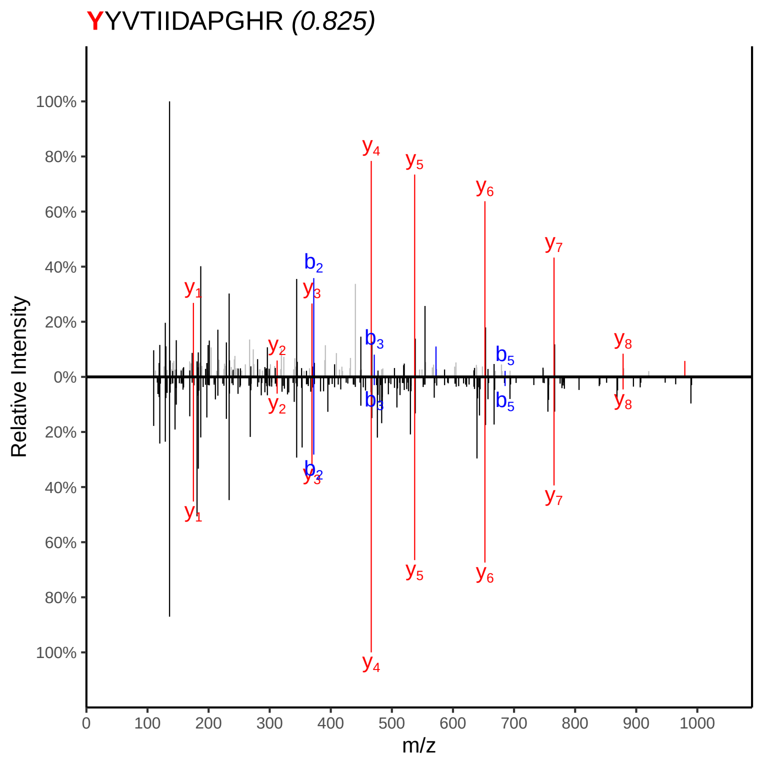

YYVTIIDAPGHR (0.825)
Relative Intensity
m/z
y1
y2
b2
y3
b3
y4
y5
y6
b5
y7
y8

TGHSLLHTLYGR *(0.807)*

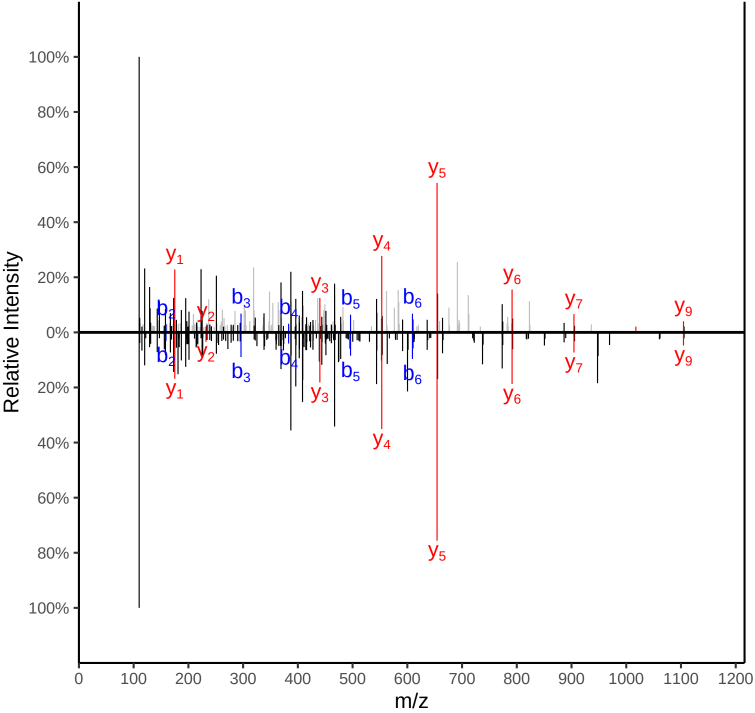

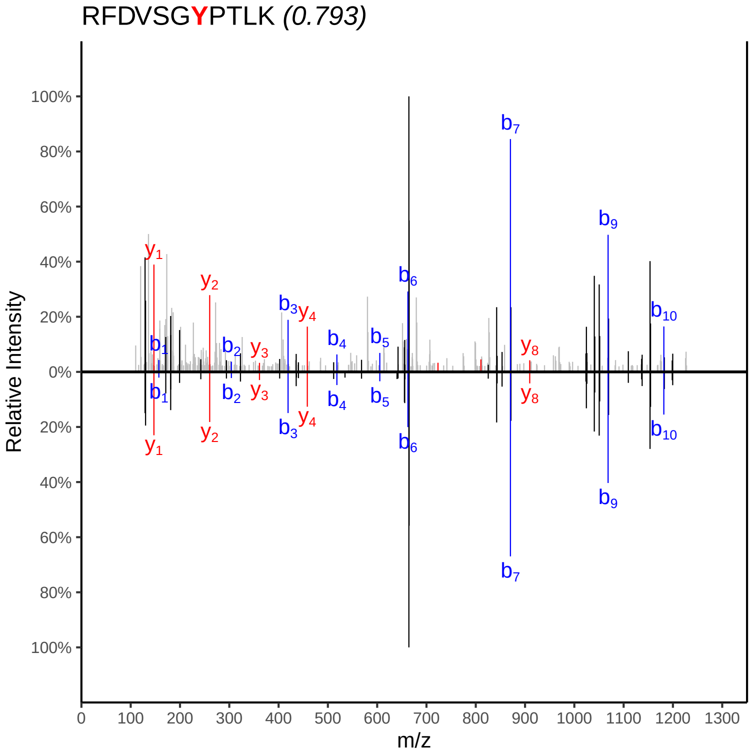

RFDVSGYPTLK (0.793)
Relative Intensity
m/z
100%
80%
60%
40%
20%
0%
y1
y2
y3
y4
y8
b1
b2
b3
b4
b5
b6
b7
b9
b10
0
100
200
300
400
500
600
700
800
900
1000
1100
1200
1300

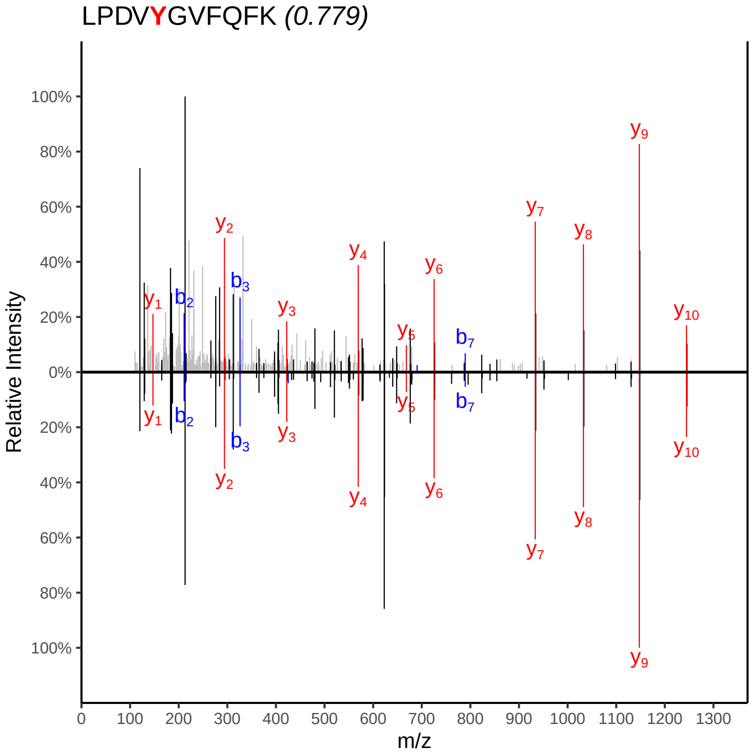

LPDVYGVFQFK (0.779)
Relative Intensity
m/z
100%
80%
60%
40%
20%
0%
0
100
200
300
400
500
600
700
800
900
1000
1100
1200
1300
y1
b2
y2
b3
y3
y4
y5
y6
b7
y7
y8
y9
y10

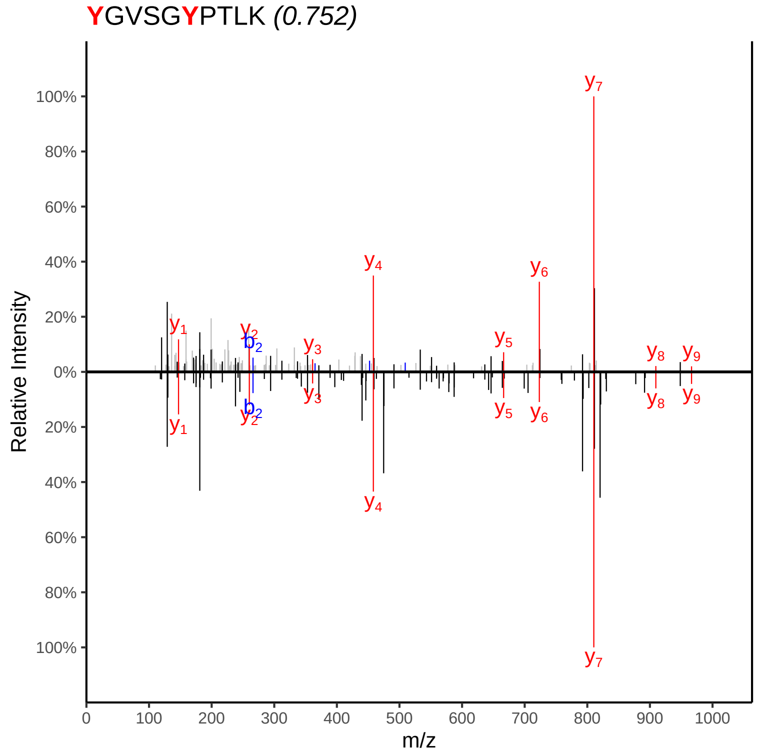
YGVSGYPTLK (0.752)
Relative Intensity
m/z
100%
80%
60%
40%
20%
0%
0
100
200
300
400
500
600
700
800
900
1000
y1
y2
b2
y3
y4
y5
y6
y7
y8
y9

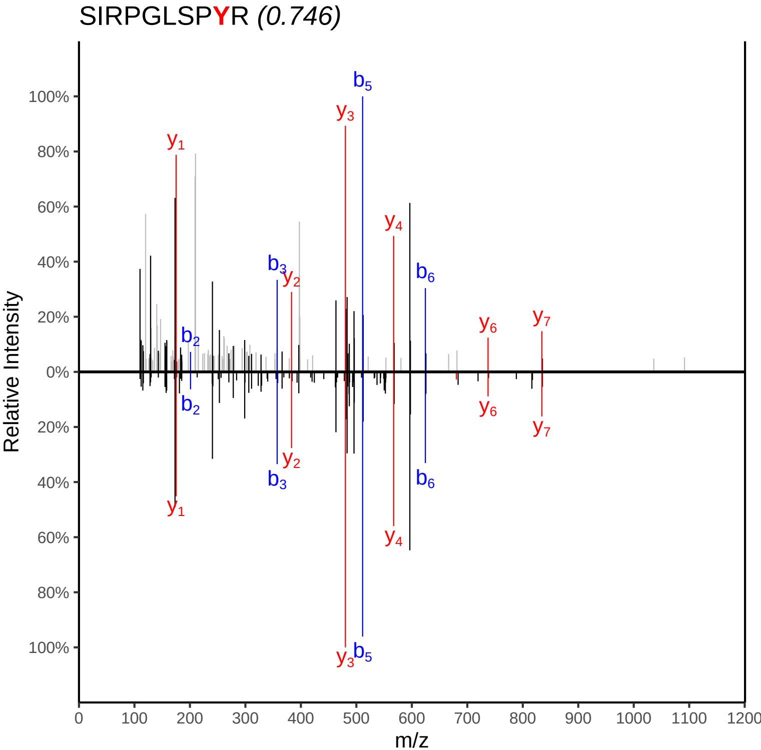

SIRPGLSPYR (0.746)
Relative Intensity
m/z
b2
b3
b5
b6
y1
y2
y3
y4
y6
y7
100%
80%
60%
40%
20%
0%
0
100
200
300
400
500
600
700
800
900
1000
1100
1200

TNYNDRYDEIR *(0.745)*

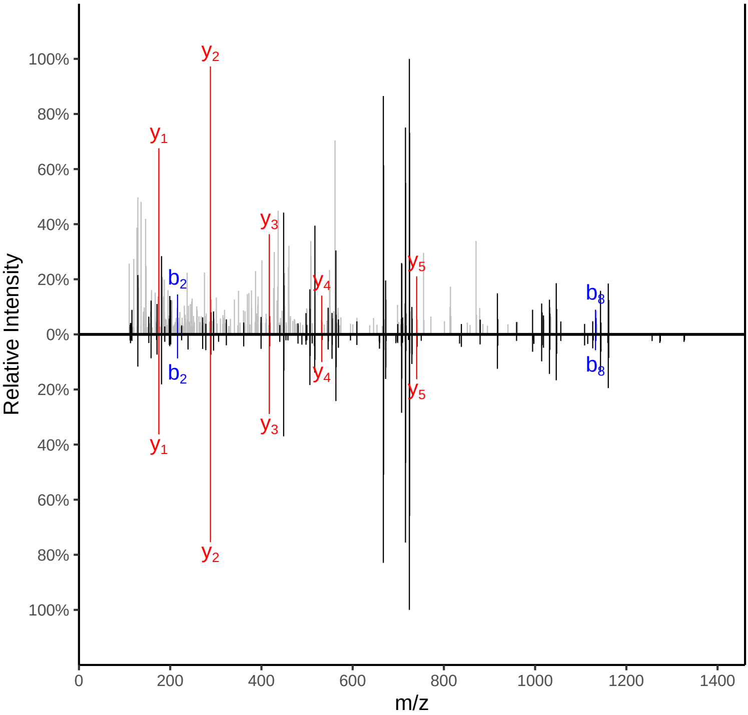

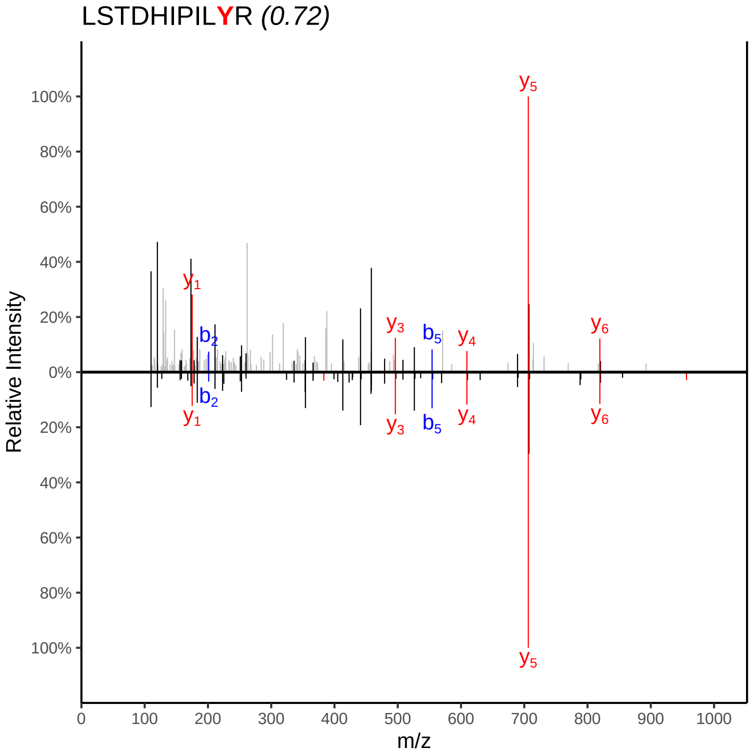

LSTDHIPILYR (0.72)
Relative Intensity
m/z
y1
b2
y3
b5
y4
y5
y6
100%
80%
60%
40%
20%
0%
0
100
200
300
400
500
600
700
800
900
1000

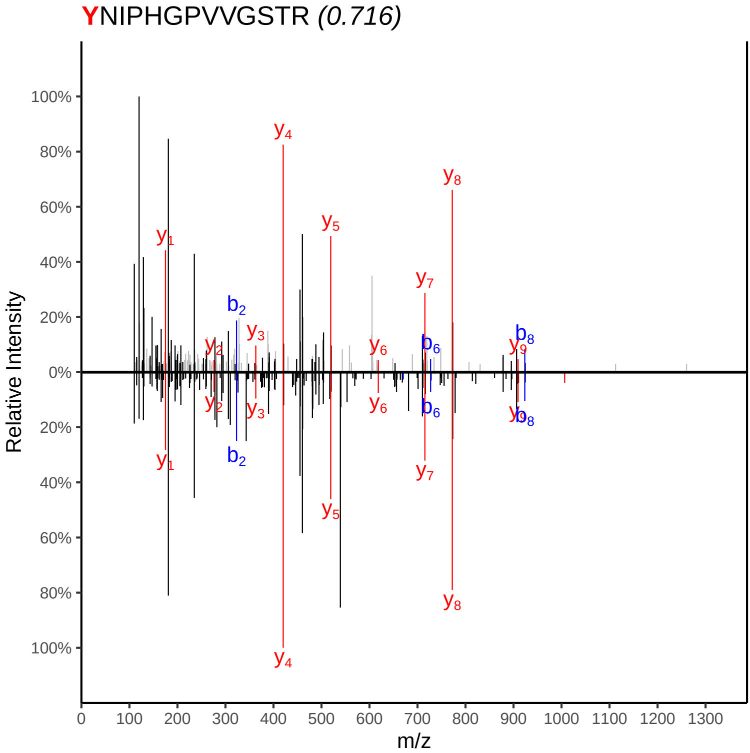

YNIPHGPVVGSTR (0.716)
Relative Intensity
m/z
100%
80%
60%
40%
20%
0%
0
100
200
300
400
500
600
700
800
900
1000
1100
1200
1300
y1
y2
y3
y4
y5
y6
y7
y8
y9
b2
b6
b8

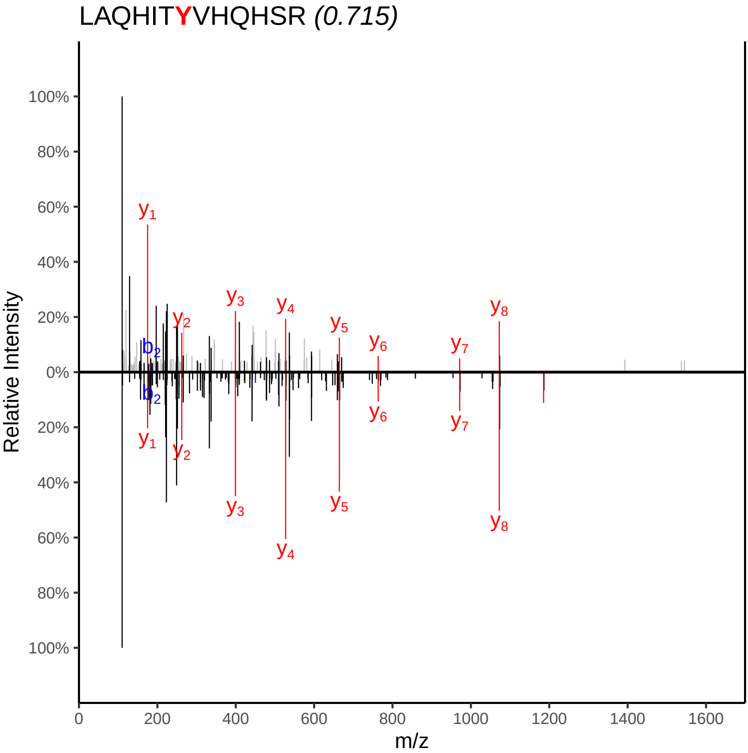
LAQHITYVHQHSR (0.715)
Relative Intensity
m/z
100%
80%
60%
40%
20%
0%
20%
40%
60%
80%
100%
0
200
400
600
800
1000
1200
1400
1600
y1
y2
y3
y4
y5
y6
y7
y8
b2

TTPSYVAFTDTER *(0.686)*

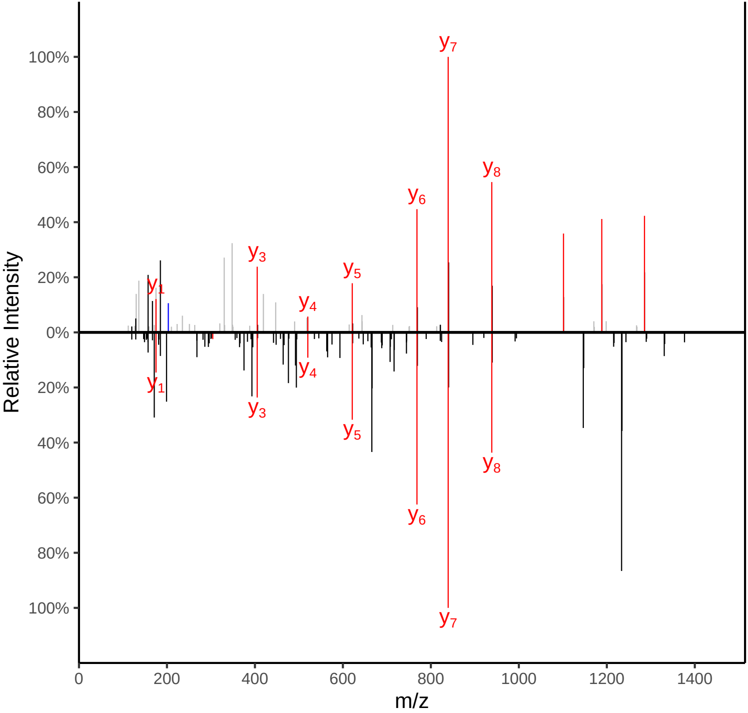

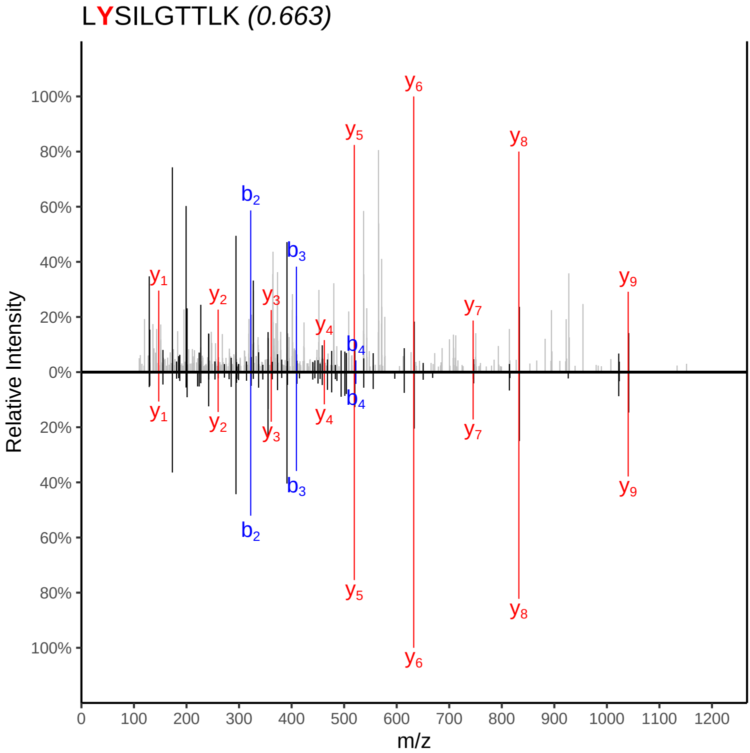
LYSILGTTLK (0.663)
Relative Intensity
m/z
100%
80%
60%
40%
20%
0%
0
100
200
300
400
500
600
700
800
900
1000
1100
1200
y1
y2
y3
y4
y5
y6
y7
y8
y9
b2
b3
b4

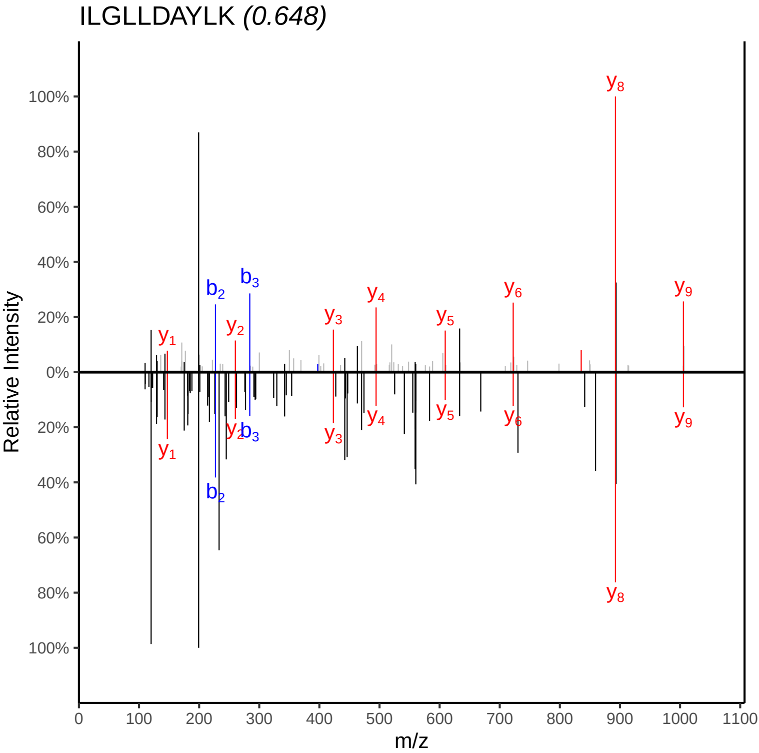

ILGLLDAYLK (0.648)
Relative Intensity
m/z
100%
80%
60%
40%
20%
0%
20%
40%
60%
80%
100%
0
100
200
300
400
500
600
700
800
900
1000
1100
y1
y2
b2
b3
y3
y4
y5
y6
y8
y9

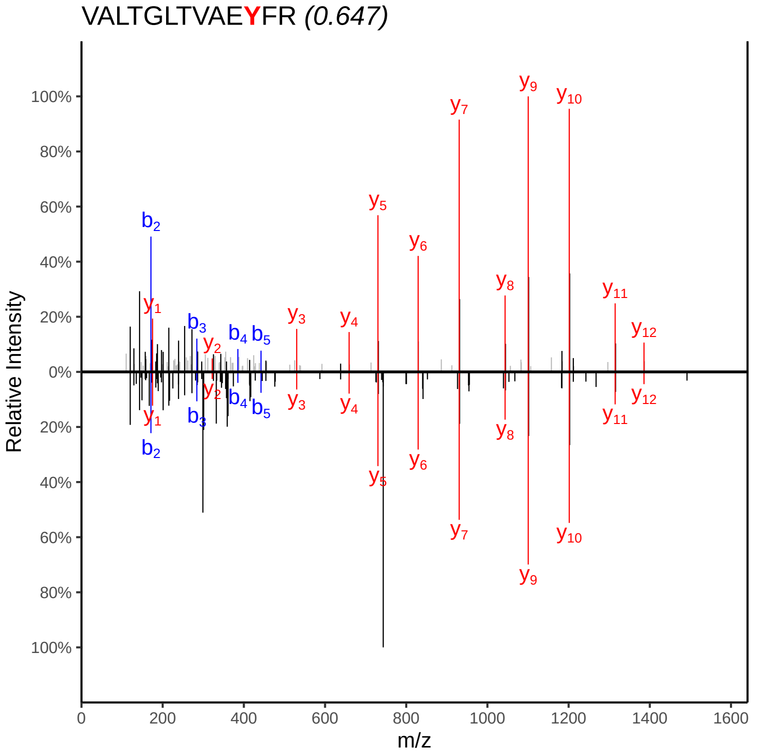

VALTGLTVAEYFR (0.647)
Relative Intensity
m/z
100%
80%
60%
40%
20%
0%
0
200
400
600
800
1000
1200
1400
1600
b2
b3
b4
b5
y1
y2
y3
y4
y5
y6
y7
y8
y9
y10
y11
y12

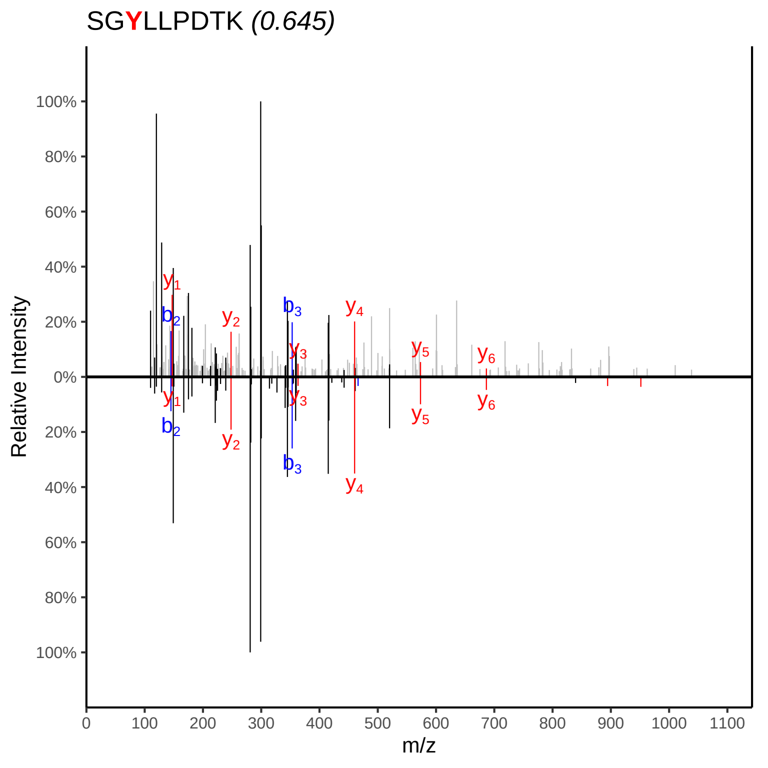
SGYLLPDTK (0.645)
Relative Intensity
100%
80%
60%
40%
20%
0%
20%
40%
60%
80%
100%
y1
b2
y2
b3
y3
y4
y5
y6
0
100
200
300
400
500
600
700
800
900
1000
1100
m/z

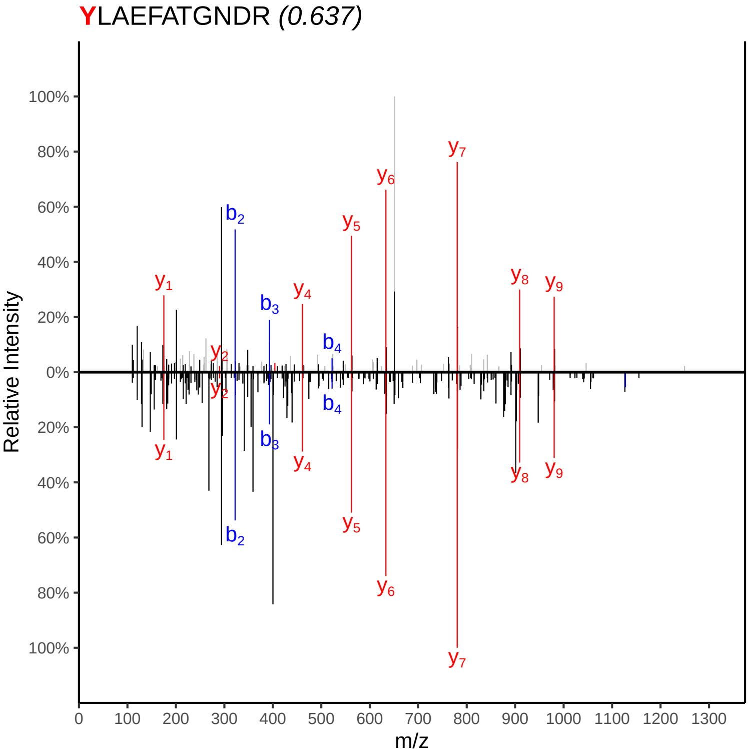
YLAEFATGNDR (0.637)
Relative Intensity
m/z
y1
y2
b2
b3
y4
b4
y5
y6
y7
y8
y9
100%
80%
60%
40%
20%
0%
0
100
200
300
400
500
600
700
800
900
1000
1100
1200
1300

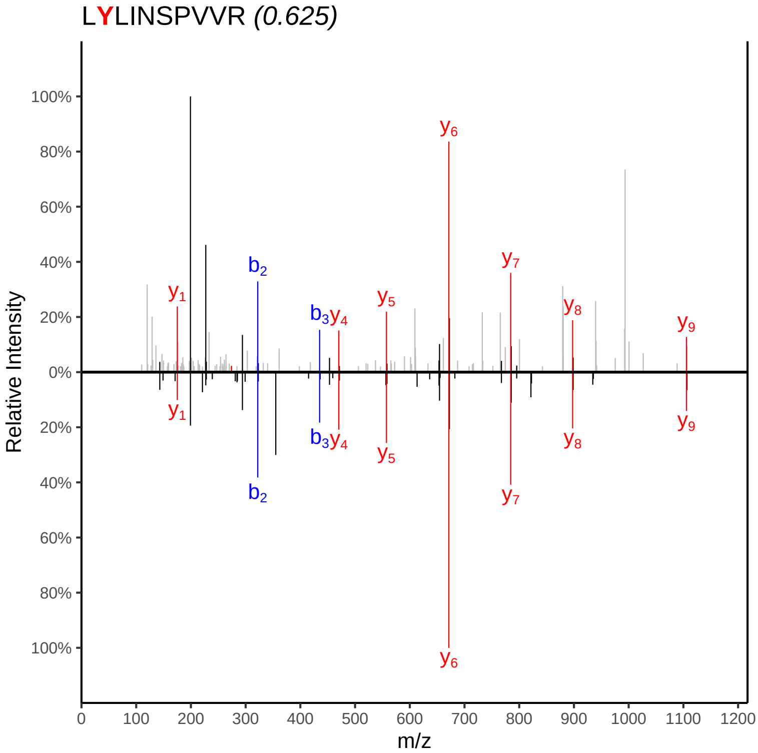
LYLINSPVVR (0.625)
Relative Intensity
m/z
100%
80%
60%
40%
20%
0%
0
100
200
300
400
500
600
700
800
900
1000
1100
1200
y1
b2
b3y4
y5
y6
y7
y8
y9

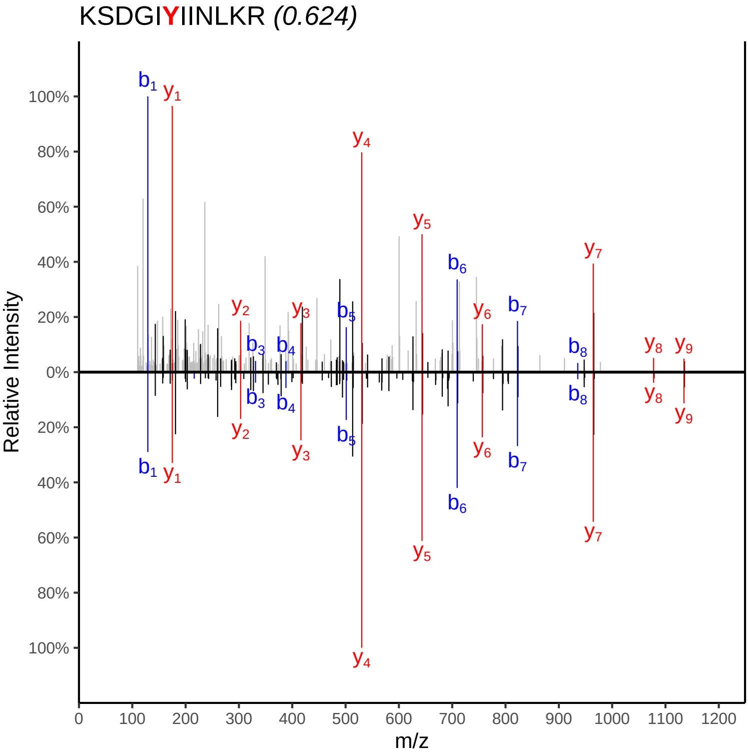
KSDGIYIINLKR (0.624)
Relative Intensity
m/z
b1 y1 y4 y5 b6 y7 y2 b3 b4 y3 b5 y6 b7 b8 y8 y9
100%
80%
60%
40%
20%
0%
0 100 200 300 400 500 600 700 800 900 1000 1100 1200

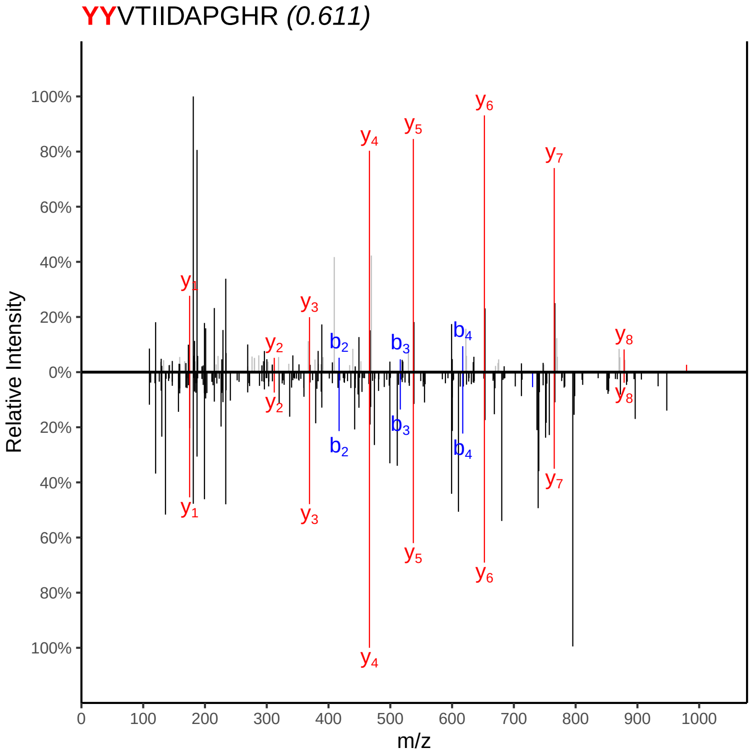
YYVTIIDAPGHR (0.611)
Relative Intensity
m/z
100%
80%
60%
40%
20%
0%
20%
40%
60%
80%
100%
0
100
200
300
400
500
600
700
800
900
1000
y1
y2
y3
b2
y4
b3
y5
b4
y6
y7
y8

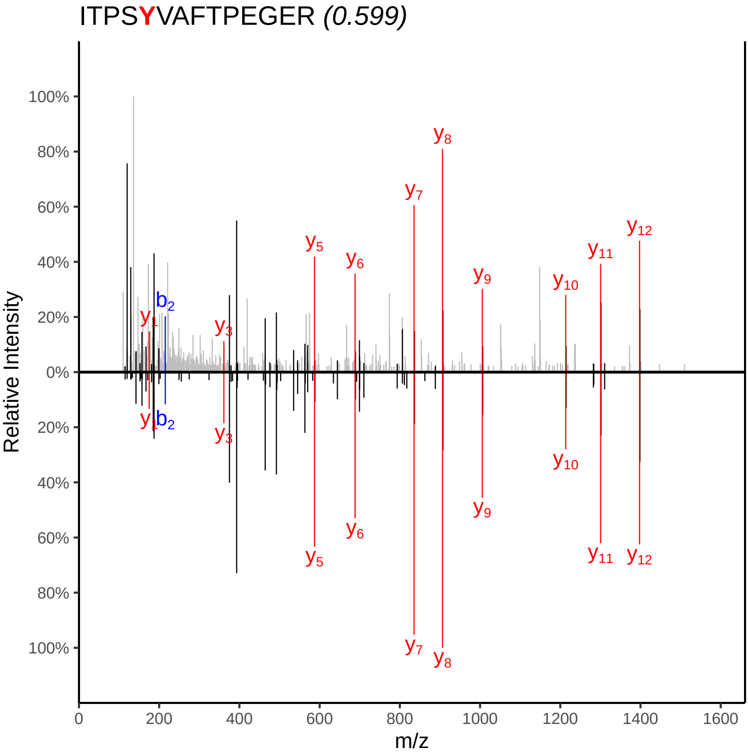
ITPSYVAFTPEGER (0.599)
Relative Intensity
100%
80%
60%
40%
20%
0%
20%
40%
60%
80%
100%
y1
b2
y3
y5
y6
y7
y8
y9
y10
y11
y12
0
200
400
600
800
1000
1200
1400
1600
m/z

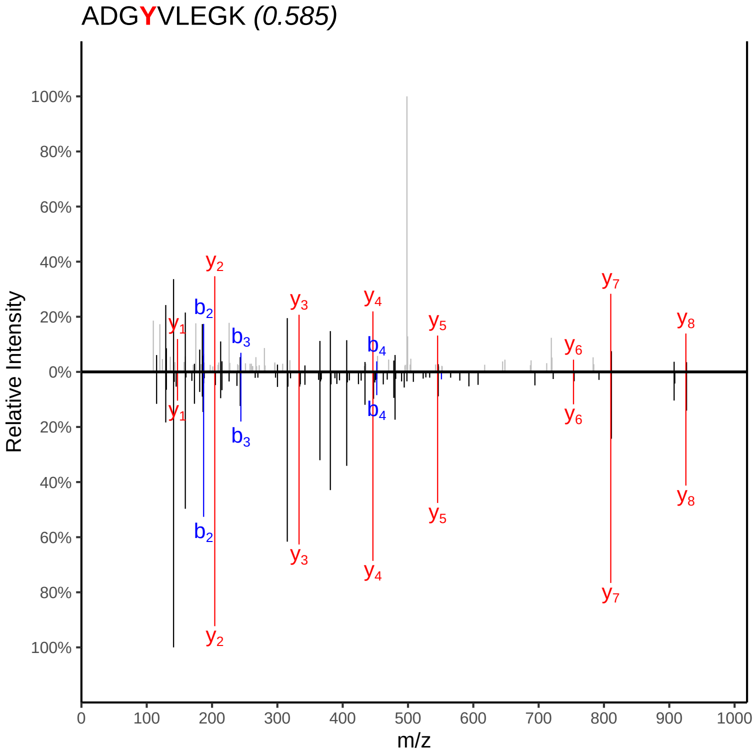

ADGYVLEGK (0.585)
Relative Intensity
m/z
100%
80%
60%
40%
20%
0%
0
100
200
300
400
500
600
700
800
900
1000
y1
y2
y3
y4
y5
y6
y7
y8
b2
b3
b4

LSQNNFALG**Y**K *(0.582)*

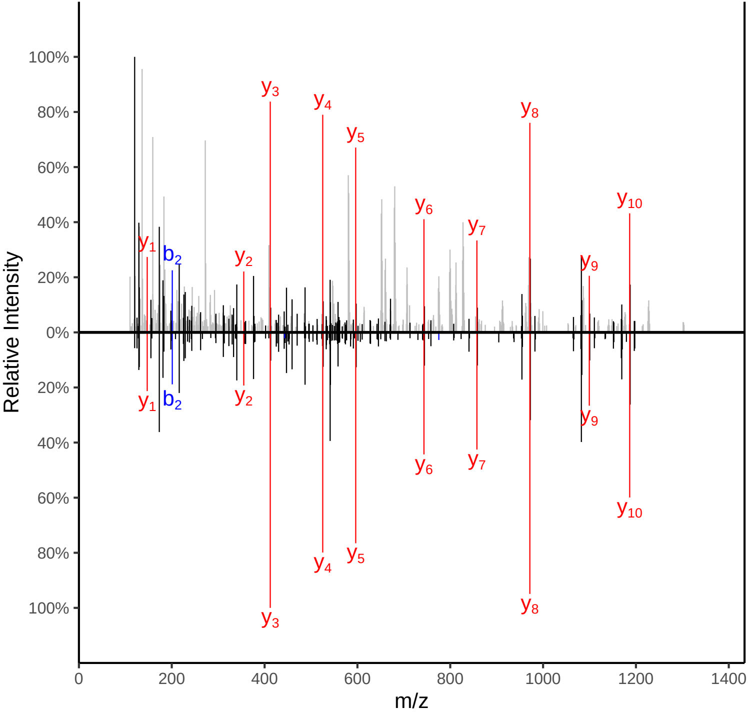

YPQLLPGIR *(0.567)*

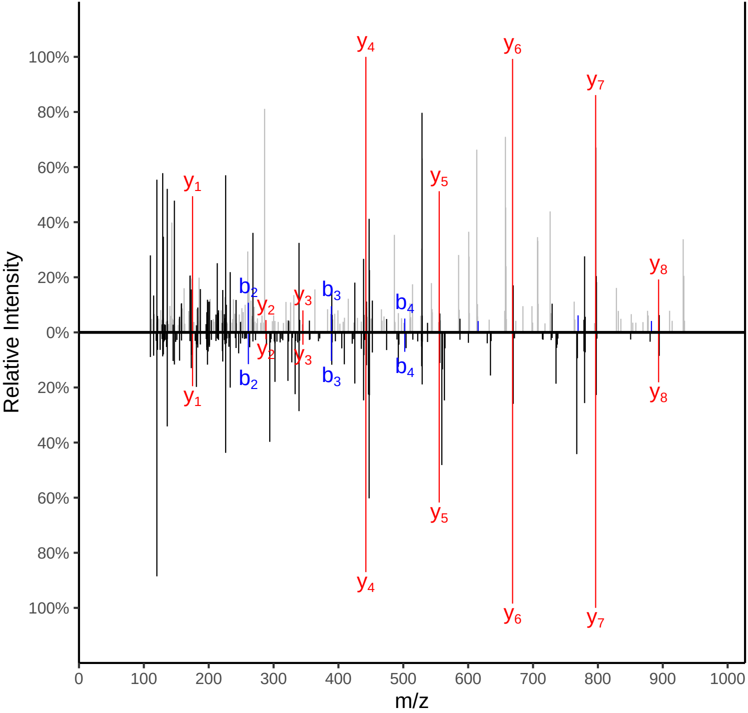

DAVTYTEHAKR *(0.54)*

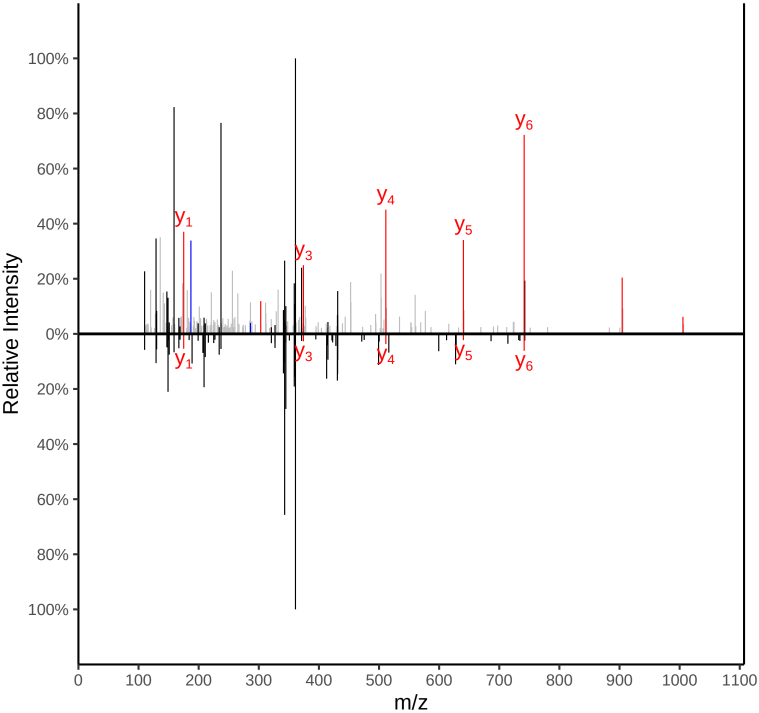

YQILPLHSQIPR *(0.49)*

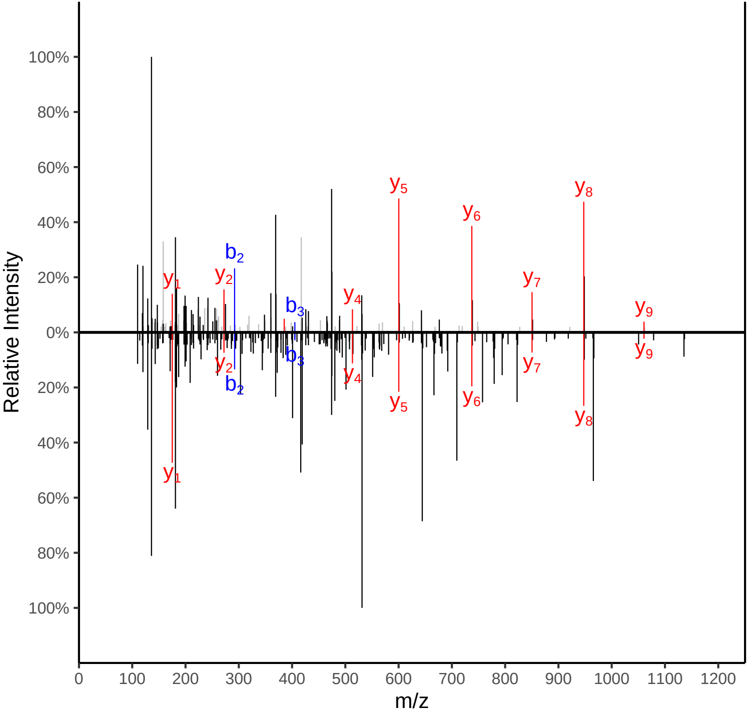

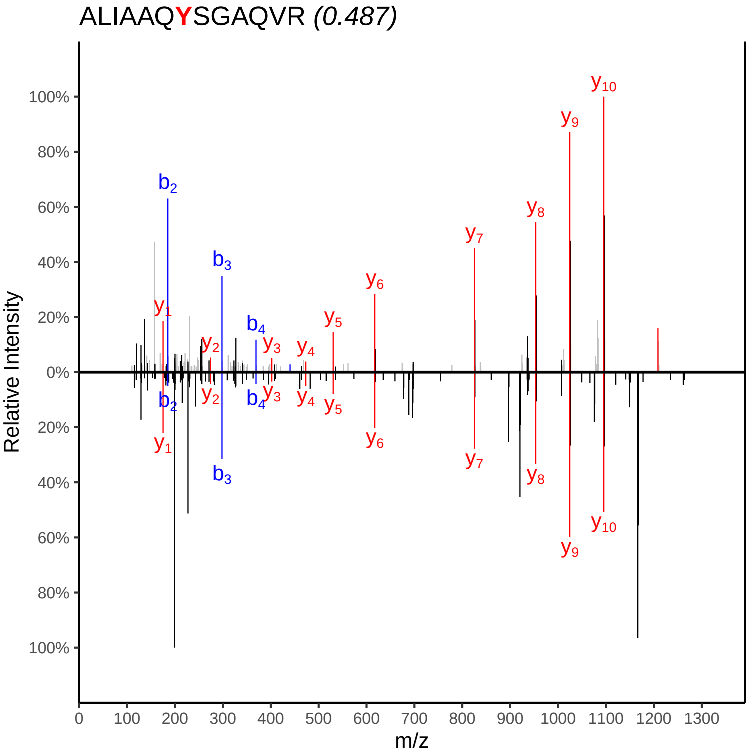

ALIAAQYSGAQVR (0.487)
Relative Intensity
m/z
100%
80%
60%
40%
20%
0%
20%
40%
60%
80%
100%
0
100
200
300
400
500
600
700
800
900
1000
1100
1200
1300
b2
b3
b4
y1
y2
y3
y4
y5
y6
y7
y8
y9
y10

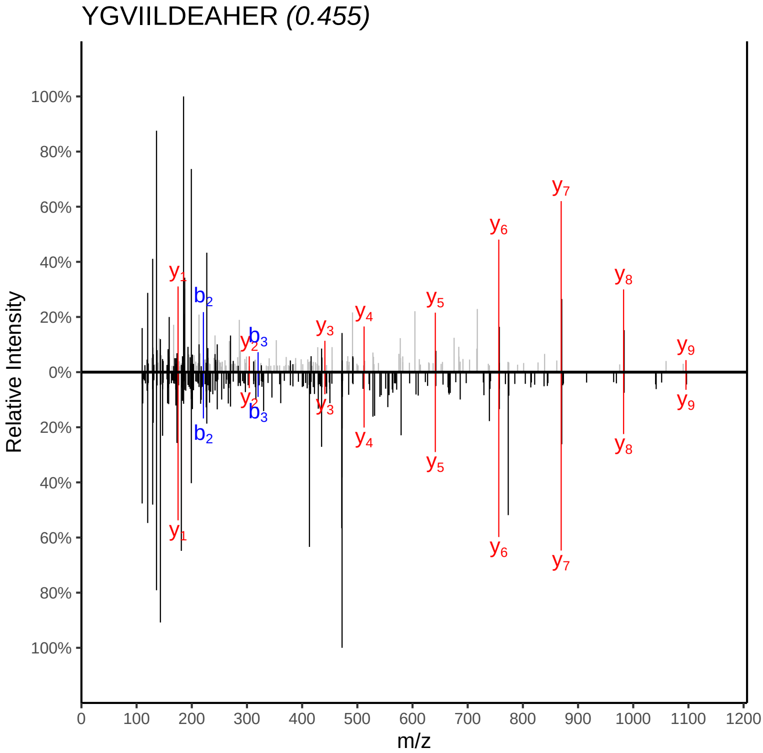
YGVIILDEAHER (0.455)
Relative Intensity
100%
80%
60%
40%
20%
0%
20%
40%
60%
80%
100%
m/z
0
100
200
300
400
500
600
700
800
900
1000
1100
1200
y1
b2
y2
b3
y3
y4
y5
y6
y7
y8
y9

YYVTIIDAPGHR *(0.446)*

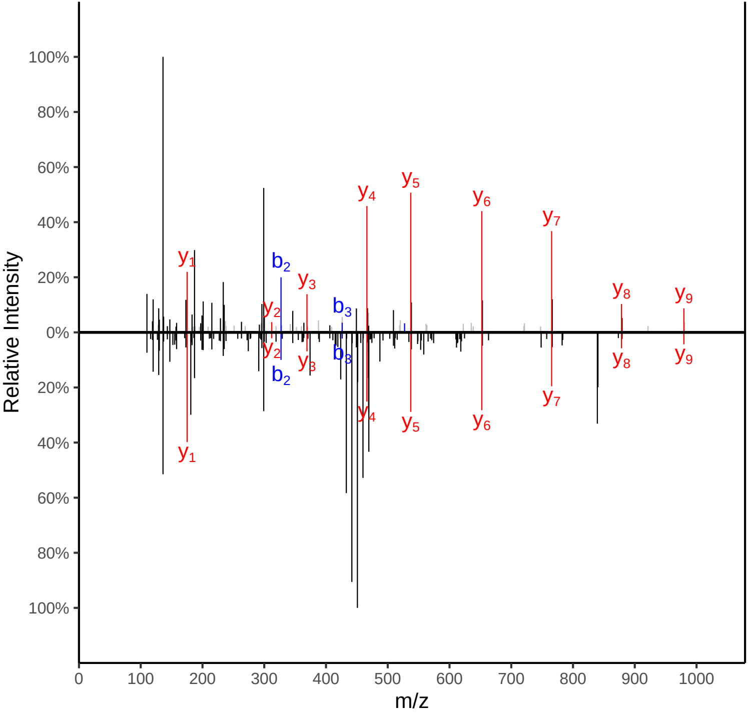

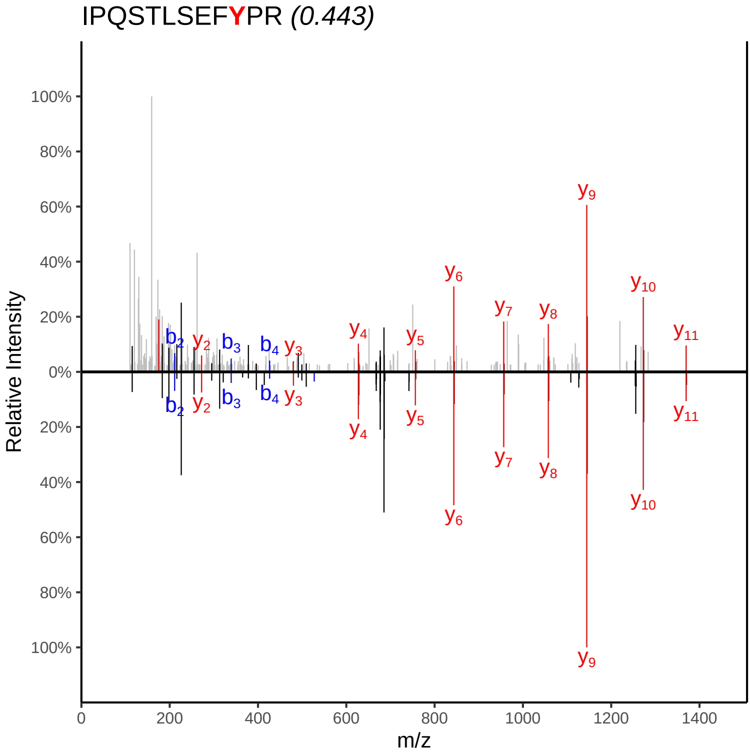

IPQSTLSEFYPR (0.443)
Relative Intensity
m/z
100%
80%
60%
40%
20%
0%
0
200
400
600
800
1000
1200
1400
b2
b3
b4
y2
y3
y4
y5
y6
y7
y8
y9
y10
y11

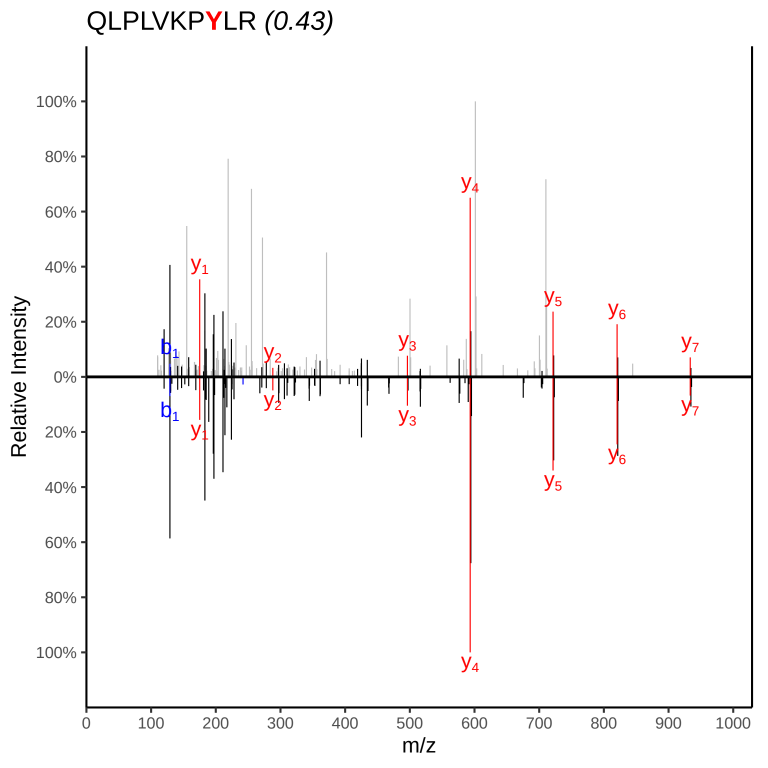
QLPLVKPYLR (0.43)
Relative Intensity
m/z
b1
y1
y2
y3
y4
y5
y6
y7
100%
80%
60%
40%
20%
0%
0
100
200
300
400
500
600
700
800
900
1000

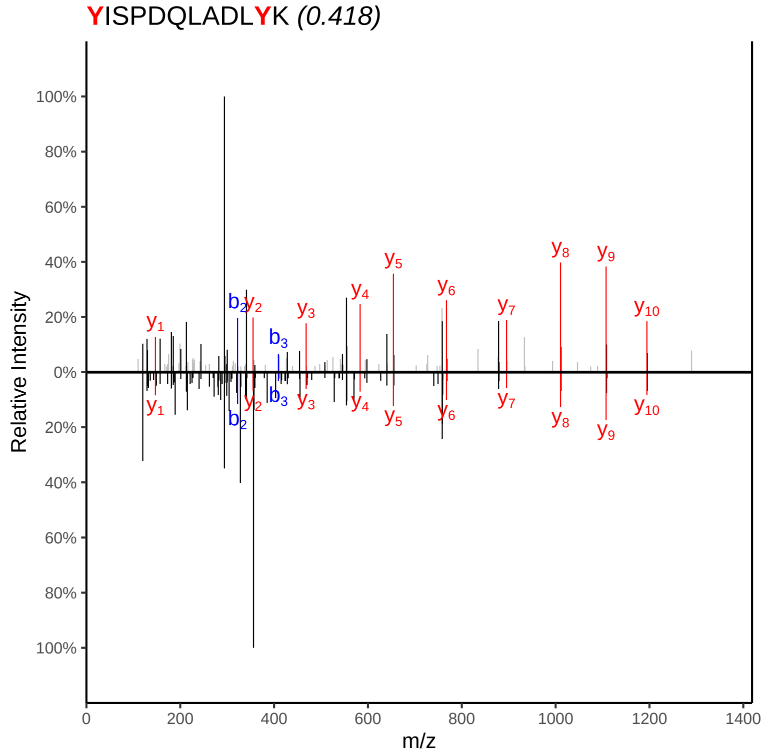

YISPDQLADLYK (0.418)
Relative Intensity
m/z
100%
80%
60%
40%
20%
0%
0
200
400
600
800
1000
1200
1400
y1
y2
y3
y4
y5
y6
y7
y8
y9
y10
b2
b3

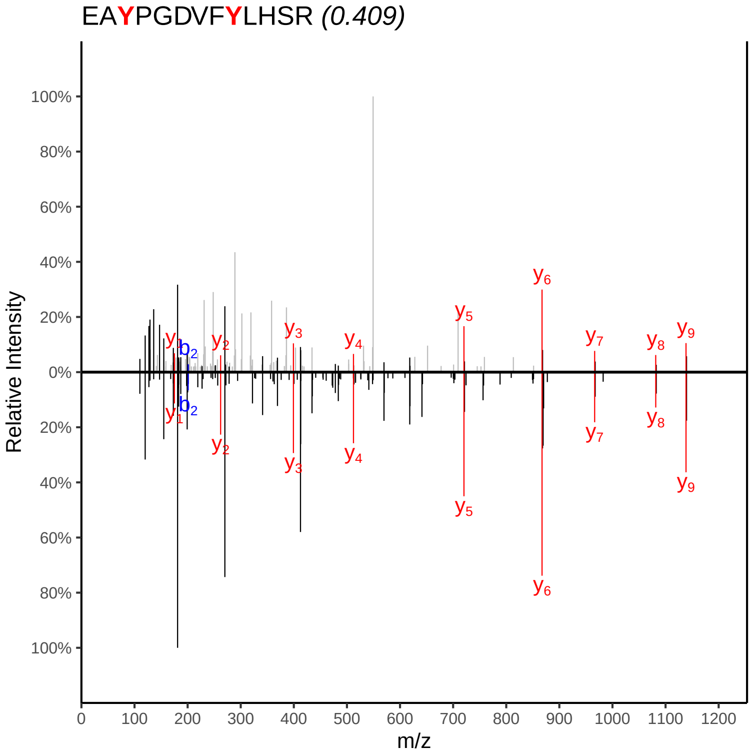
EAYPGDVFYLHSR (0.409)
Relative Intensity
m/z
100%
80%
60%
40%
20%
0%
y1
b2
y2
y3
y4
y5
y6
y7
y8
y9
0
100
200
300
400
500
600
700
800
900
1000
1100
1200

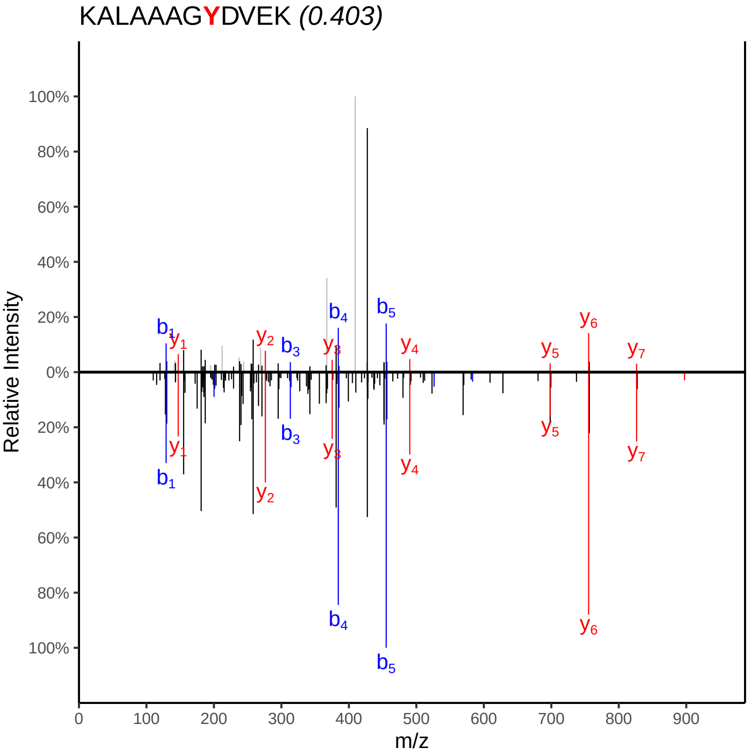

KALAAAGYDVEK (0.403)
Relative Intensity
m/z
100%
80%
60%
40%
20%
0%
0
100
200
300
400
500
600
700
800
900
b1
b3
b4
b5
y1
y2
y3
y4
y5
y6
y7

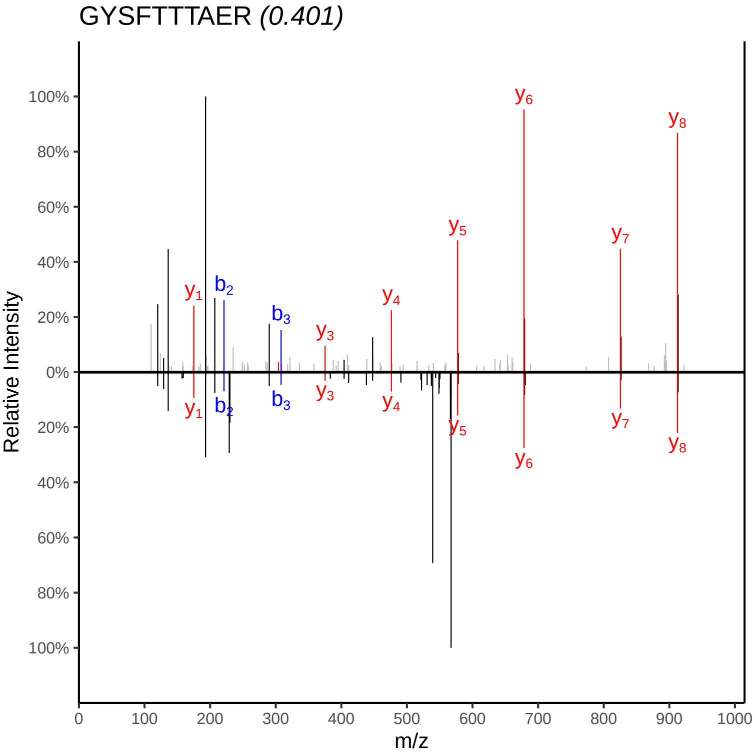

GYSFTTTAER (0.401)
Relative Intensity
m/z
100%
80%
60%
40%
20%
0%
20%
40%
60%
80%
100%
0
100
200
300
400
500
600
700
800
900
1000
y1
b2
b3
y3
y4
y5
y6
y7
y8

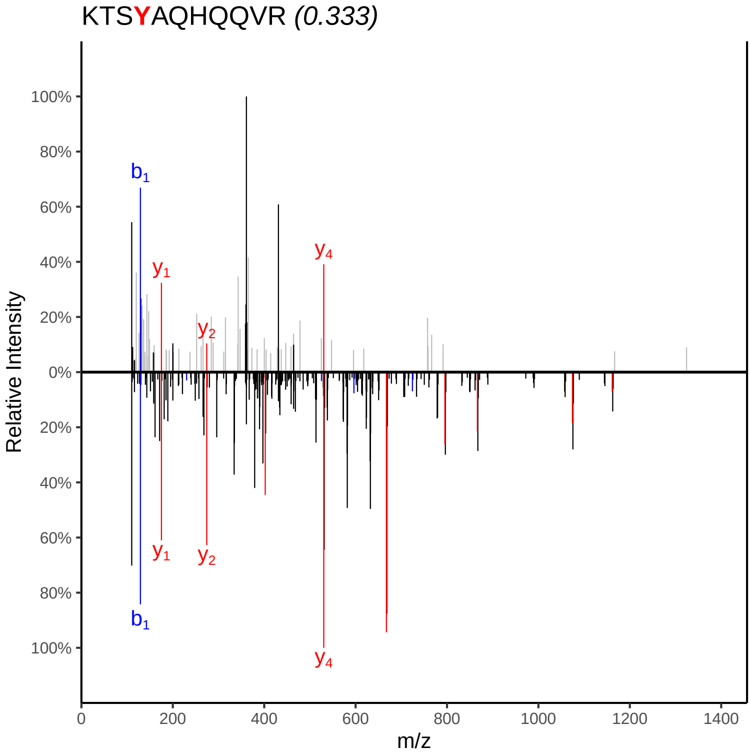
KTSYAQHQQVR (0.333)
Relative Intensity
m/z
100%
80%
60%
40%
20%
0%
20%
40%
60%
80%
100%
0
200
400
600
800
1000
1200
1400
b_1
y_1
y_2
y_4
y_1
y_2
b_1
y_4

DAVT**Y**TEHAKR *(0.323)*

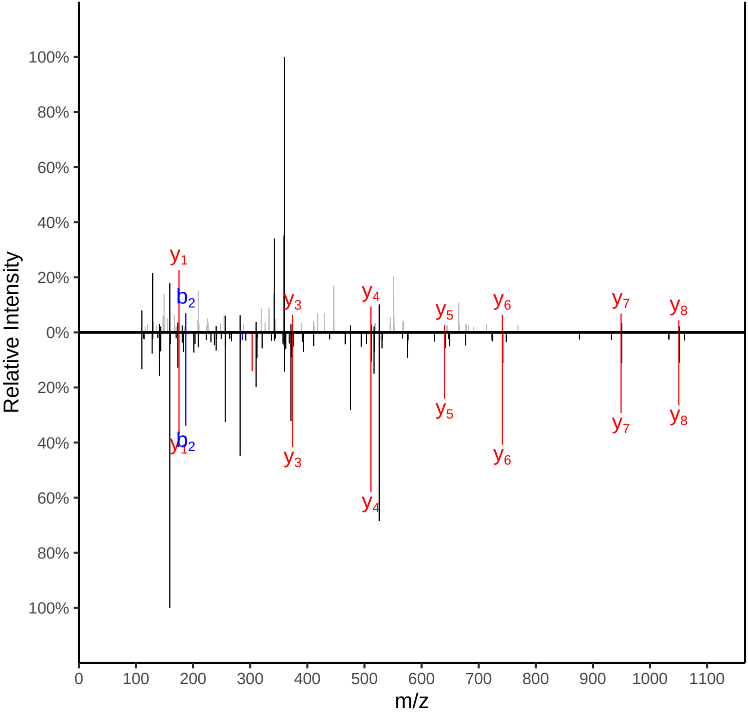

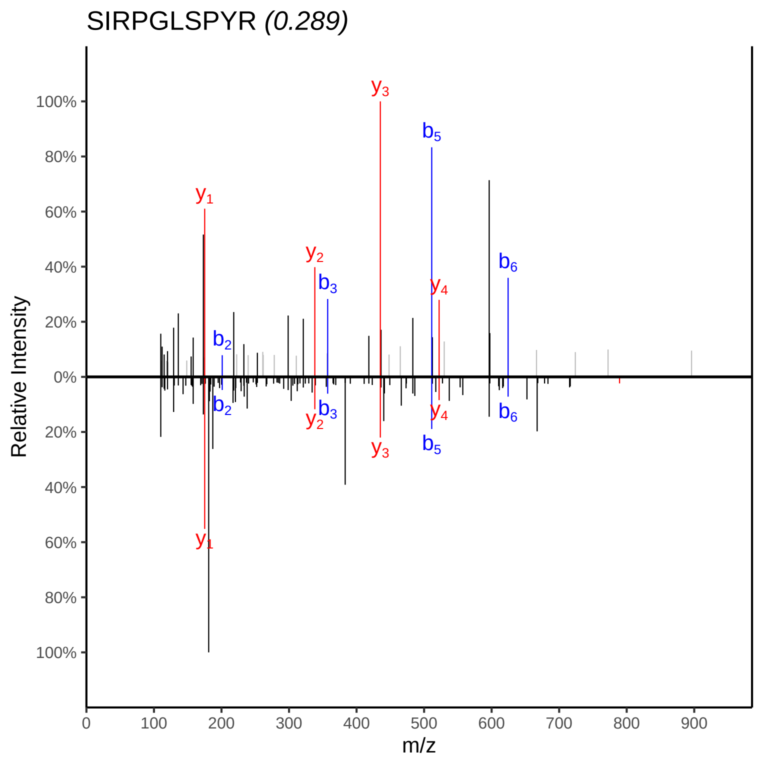

SIRPGLSPYR (0.289)
Relative Intensity
m/z
100%
80%
60%
40%
20%
0%
20%
40%
60%
80%
100%
0
100
200
300
400
500
600
700
800
900
y1
y2
y3
y4
b2
b3
b5
b6

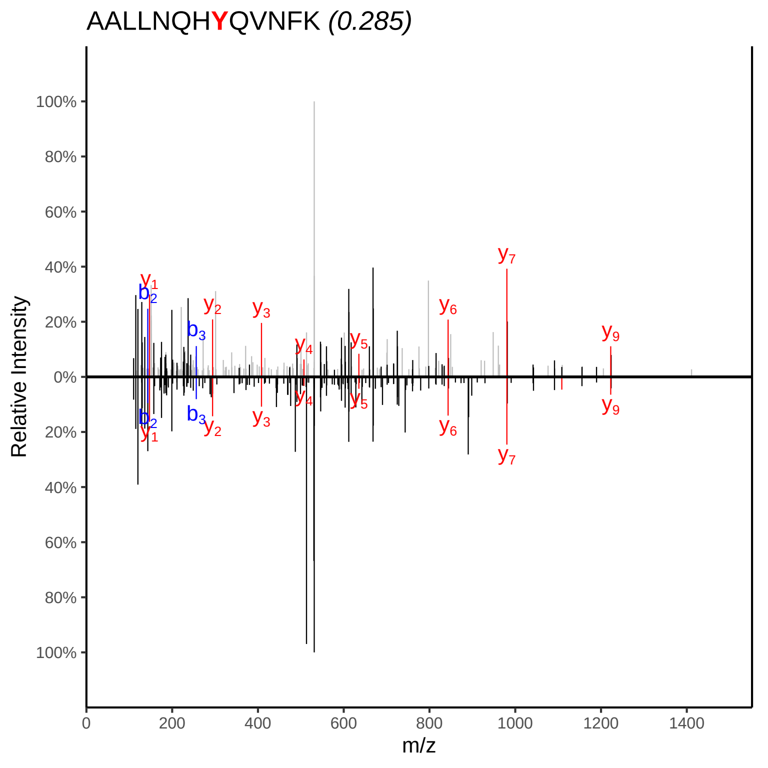

AALLNQHYQVNFK (0.285)
Relative Intensity
100%
80%
60%
40%
20%
0%
20%
40%
60%
80%
100%
m/z
0
200
400
600
800
1000
1200
1400
y1
b2
b3
y2
y3
y4
y5
y6
y7
y9

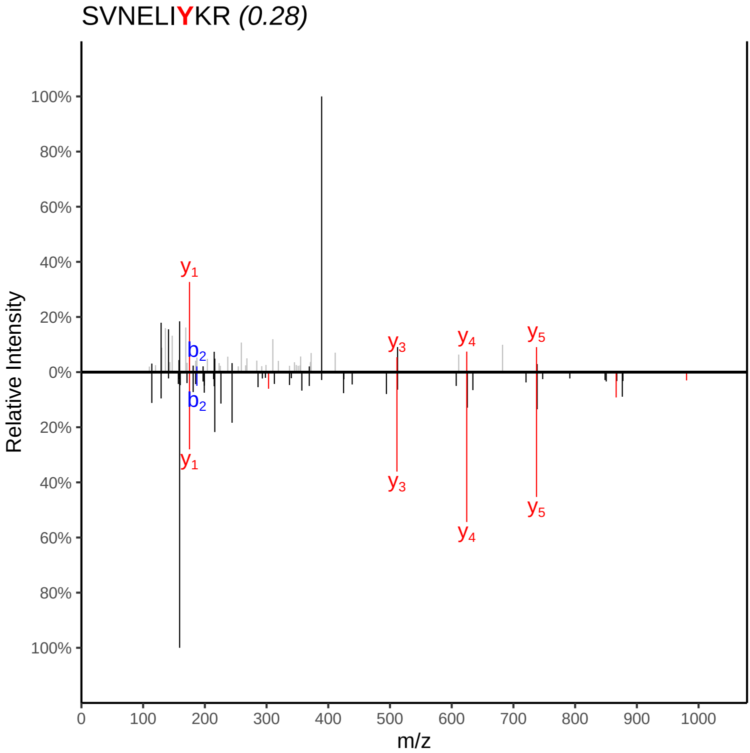

SVNELIYKR (0.28)
Relative Intensity
100%
80%
60%
40%
20%
0%
20%
40%
60%
80%
100%
y1
b2
y3
y4
y5
b2
y1
y3
y5
y4
0
100
200
300
400
500
600
700
800
900
1000
m/z

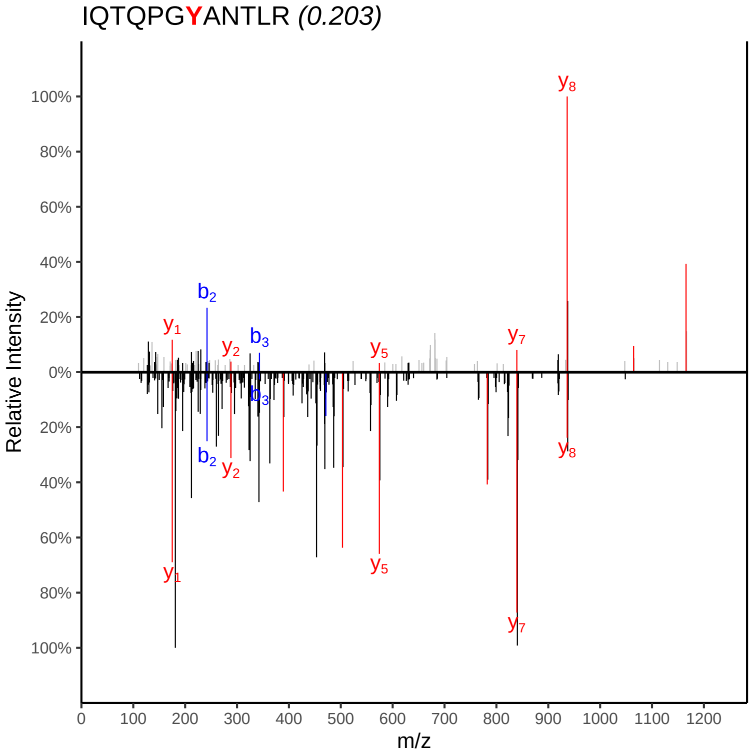

IQTQPGYANTLR (0.203)
Relative Intensity
m/z
100%
80%
60%
40%
20%
0%
20%
40%
60%
80%
100%
0
100
200
300
400
500
600
700
800
900
1000
1100
1200
y1
b2
y2
b3
y5
y7
y8
