# Supplementary material for: Exploration of Nitrotyrosine-Containing Proteins and Peptides by Antibody-Based Enrichment Strategies
Source: Mol Cell Proteomics. 2024 Feb 10;23(3):100733. doi: 10.1016/j.mcpro.2024.100733 (PMC10950883; doi:10.1016/j.mcpro.2024.100733)
Supplement: Supplementary Figure legends [file mmc2.docx]

**Supplementary Figure Legends**

**Supplementary Figure 1.** Nitrotyrosine-containing peptides identified in one, two or three replicates from (**A)** protein-based; or **(B)** peptide-based immunoenrichment experiments with the indicated antibodies.

**Supplementary Figure 2.** Histogram showing the distribution of spectral similarity between MS/MS spectra of synthetic nitrotyrosine peptides and experimentally identified nitrotyrosine peptides

**Supplementary Figure 3.** Mirror images obtained after performing spectral matches between MS/MS spectra of synthetic peptides and experimentally identified nitrotyrosine peptides. Cosine similarity score of each match is denoted along with annotated b and y ions.
