# Supplementary material for: Exploration of Nitrotyrosine-Containing Proteins and Peptides by Antibody-Based Enrichment Strategies
Source: Mol Cell Proteomics. 2024 Feb 10;23(3):100733. doi: 10.1016/j.mcpro.2024.100733 (PMC10950883; doi:10.1016/j.mcpro.2024.100733)
Supplement: Supplementary Table Legends [file mmc3.docx]

**Supplementary Table Legends**

**Supplementary Table 1**. (A) A list of nitrotyrosine peptides identified from protein-based immunoaffinity experiments (B) A list of nitrotyrosine peptides identified from peptide-based immunoaffinity experiments (C) A list of all nitrotyrosine peptides identified from either protein or peptide-based immunoaffinity experiments

**Supplementary Table 2**. A list of pathways, molecular functions and cellular components enriched for nitrotyrosine peptides identified from both protein and peptide-based immunoaffinity experiments
